# Reconstruction of avian ancestral karyotypes reveals differences in the evolutionary history of macro- and microchromosomes

**DOI:** 10.1186/s13059-018-1544-8

**Published:** 2018-10-05

**Authors:** Joana Damas, Jaebum Kim, Marta Farré, Darren K Griffin, Denis M Larkin

**Affiliations:** 1 Department of Comparative Biomedical Sciences, Royal Veterinary College, University of London, London, NW1 0TU UK; 2 Department of Biomedical Science and Engineering, Konkuk University, Seoul, 05029 South Korea; 3 School of Biosciences, University of Kent, Canterbury, CT2 7NY UK

**Keywords:** Ancestral karyotypes, Avian, Chromosome evolution, Evolutionary breakpoint regions, Homologous synteny blocks

## Abstract

**Background:**

Reconstruction of ancestral karyotypes is critical for our understanding of genome evolution, allowing for the identification of the gross changes that shaped extant genomes. The identification of such changes and their time of occurrence can shed light on the biology of each species, clade and their evolutionary history. However, this is impeded by both the fragmented nature of the majority of genome assemblies and the limitations of the available software to work with them. These limitations are particularly apparent in birds, with only 10 chromosome-level assemblies reported thus far. Algorithmic approaches applied to fragmented genome assemblies can nonetheless help define patterns of chromosomal change in defined taxonomic groups.

**Results:**

Here, we make use of the DESCHRAMBLER algorithm to perform the first large-scale study of ancestral chromosome structure and evolution in birds. This algorithm allows us to reconstruct the overall genome structure of 14 key nodes of avian evolution from the Avian ancestor to the ancestor of the Estrildidae, Thraupidae and Fringillidae families.

**Conclusions:**

Analysis of these reconstructions provides important insights into the variability of rearrangement rates during avian evolution and allows the detection of patterns related to the chromosome distribution of evolutionary breakpoint regions. Moreover, the inclusion of microchromosomes in our reconstructions allows us to provide novel insights into the evolution of these avian chromosomes, specifically.

**Electronic supplementary material:**

The online version of this article (10.1186/s13059-018-1544-8) contains supplementary material, which is available to authorized users.

## Background

Reconstructions of ancestral chromosome structures, utilising traditional cytogenetics or genetic map comparisons, offered the first insights into the evolutionary events that shaped extant animal gross genome organisation and the mechanisms that drive chromosome evolution. Indeed, cytogenetics (‘zoo-FISH’) information proved useful to describe basic ancestral patterns, e.g. in placental mammals [1] and birds [2] but has limited sensitivity. Differences in rearrangement rates and chromosome structures revealed from these reconstructions, nonetheless, pointed to variation in levels of genome reshuffling and prevailing types of chromosome changes in the evolution of distinct and related phylogenetic clades [3]. Notable examples include widespread interchromosomal rearrangements in mammals, illustrated by a high variation in chromosome numbers, from six in Indian muntjac (*Muntiacus muntjac* [4]) to 102 in Vizcacha rat (*Tympanoctomys barrerae* [5]). Contrastingly, in birds, interchromosomal rearrangements are very rare with around two thirds of all species studied so far having similar chromosome numbers and karyotypic patterns [2]. These observations raise questions regarding possible differences in the mechanisms that drive the evolution of these lineages.

The low resolution of cytogenetic methodologies inevitably leads to undetected intrachromosomal rearrangements and limited usefulness of karyotypic comparisons for the reconstruction of older ancestors (e.g. eutherian, avian and amniote ancestors). These restrictions can be overcome by the inclusion of higher-resolution genome sequence maps, which (a) expand the evolutionary depth of ancestral karyotype reconstructions and (b) increase the resolution and often accuracy at which genome rearrangements are identified. The first attempts to reconstruct ancestral karyotypes from sequence data were performed for mammals [6, 7]. These reconstructions allowed the identification of novel genome rearrangements and the detection of varied rates of change in different animal lineages. They also revealed that evolutionary breakpoint regions (EBRs) are often reused in evolution, that EBRs locate in gene-dense regions and that lineage-specific EBRs are usually associated with the location of segmental duplications in mammals [6–8]. These findings demonstrated the importance of genome sequence comparisons for the detection of the overall pattern of chromosomal events that shaped extant genomes and stimulated the development of several algorithms to perform the reconstruction of ancestral karyotypes based on genome sequence data. Most algorithms used for the inference of ancestral karyotypes, such as InferCARs [9] or ANGES [10], are optimised for chromosome-level genome assemblies (i.e. one scaffold per chromosome) and their suitability to deal with sub-chromosome (e.g. scaffold-level) genome assemblies is very limited [11]. While there have been many newly sequenced genomes released in the last few years, only a small number of them were assembled to chromosomes and were of use for ancestral karyotype reconstruction [11]. To overcome these limitations, Kim and colleagues developed DESCHRAMBLER [11]. This algorithm, in contrast to those aforementioned, is optimised to generate reconstructed ancestral chromosome fragments (RACFs) using information from both chromosome- and scaffold-level genome assemblies [11]. DESCHRAMBLER has been previously applied to the genomes of 19 mammalian species (12 chromosome-level and seven scaffold-level) to reconstruct the chromosome structure of seven eutherian ancestors. DESCHRAMBLER detected a significant number of intrachromosomal changes that cytogenetic studies could not identify. Moreover, it showed that, amongst the 10 orders studied, the primates had the largest number of chromosomes structurally identical to the Eutherian ancestor (orangutan exhibiting the largest) with chimps displaying more structural changes than humans [11].

Limited availability of chromosome-level assemblies for Aves has hitherto restricted the study of chromosome evolution in this class. Indeed, to date, the reconstruction of avian ancestral chromosome structures has been based on molecular cytogenetic comparisons [2] with sequence-based reconstructions limited to several macrochromosomes only and based on just six (four chromosome- and two scaffold-based) assemblies [12]. Thus, the extent to which individual chromosomes (particularly microchromosomes) have remained unchanged or rearranged intrachromosomally remains unknown for most avian lineages. Moreover, the sequence features affecting the stability and dynamics of avian chromosomes during evolution remained under-explored. Nonetheless, the current availability of ~ 60 avian genomes, of which ~ 30 have low enough assembly fragmentation to be suitable for DESCHRAMBLER ancestral genome reconstructions, has the potential to rectify this problem.

In this study, we report the use of DESCHRAMBLER [11] for the first large-scale study of ancestral chromosome structure and evolution in birds. The large number of avian genome assemblies included in this study and extended sampling of the avian phylogenetic tree allowed the reconstruction of the likely overall genome structure of 14 nodes in avian phylogeny, from the Avian ancestor to the ancestor of Passeriformes (the most species-rich avian order) through to the zebra finch. The analysis of these reconstructions provided detailed insights into the evolutionary history of the majority of Avian ancestor chromosomes, revealed differences in rates and evolutionary times of occurrence of structural changes in micro- and macrochromosomes and identified genomic features that are associated with remarkable evolutionary stability of several avian chromosomes.

## Results

### Reconstructed ancestral chromosome fragments

**Fig. 1:**
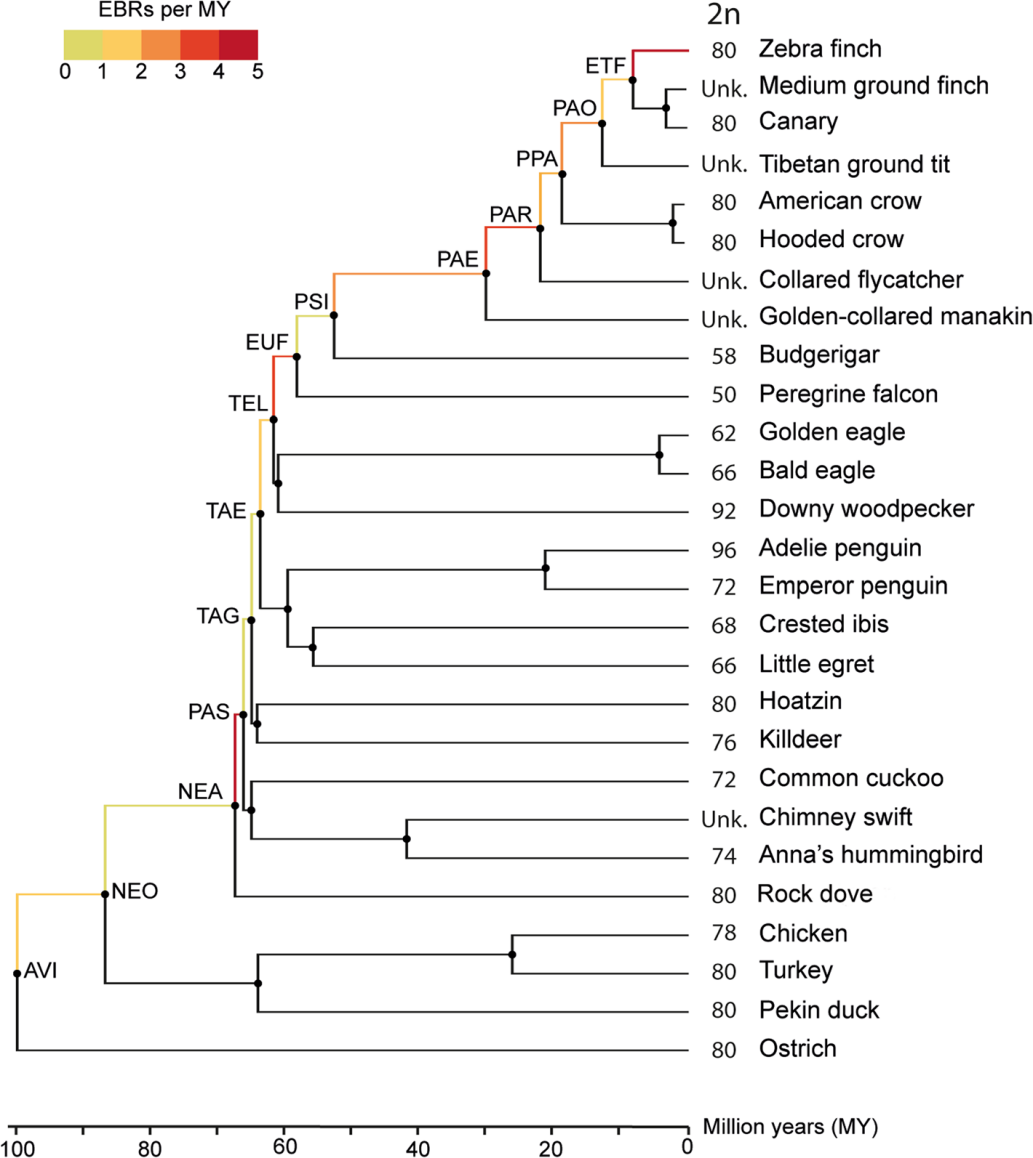
Phylogenetic tree of descendant species and reconstructed ancestors [13]. Branch colour represents rearrangement rates in RACFs (EBRs/MY). Acronyms on nodes represent ancestor names as shown in Table 1. AVI: Avian; NEO: Neognathae; NEA: Neoavian; PAS: Passerea; TAG: Telluraves & Aequornithia & Gruae; TAE: Telluraves & Aequornithia; TEL: Telluraves; EUF: Eufalconimorphae; PSI: Psittacopasserae; PAE: Passeriformes; PAR: Passeri; PPA: Passeroidea & Paroidea; PAO: Passeroidea; ETF: Estrildidae & Thraupidae & Fringillidae. Numbers next to species names indicate diploid number of chromosomes in karyotypes (if known)

To obtain a comprehensive list of the structural changes that shaped avian genomes since the Avian ancestor to the lineage leading to zebra finch, we used the DESCHRAMBLER algorithm [11] to predict ancestral chromosome structures. The ancestral chromosome reconstructions generated by DESCHAMBLER rely on the topology of phylogenetic trees [11]. Out of three phylogenetic trees tested (see the “Methods” section and Additional file 1: Supplementary Information), the TENT phylogenetic tree from Jarvis and colleagues [13] resulted in the lowest fragmentation of RACFs suggesting a higher agreement between the tree topology and our structural genomic data compared to the other tested trees (Table 1, Additional file 1: Table S3 and S4). Moreover, RACFs for ancestors with same ingroup species were highly similar regardless of the phylogenetic trees used (94–100% agreement between reconstructions; Additional file 1: Table S5 and S6). Similarly, “sister” clade ancestors (different due to inclusion/exclusion of a small number of additional species on different trees) also had similar reconstructions (82–100% similarity; Additional file 1: Table S7 and S8). The reconstructions described below were therefore obtained using the TENT tree for 14 avian ancestors (Fig. 1, Table 1).

**Table 1:** Statistics of the reconstructed ancestors (100 Kbp resolution)

Ancestor	Acronym	No. RACFs	Total length RACFs (Kbp)	Coverage (%)^a^	Longest RACF (Kbp)	Shortest RACF (Kbp)	No. SFs	Longest SF (Kbp)
Avian	AVI	79	790,916.41	77.51	90,786.44	100.10	3488	1554.45
Neognathae	NEO	54	806,245.38	79.01	92,678.53	108.57	3443	1554.45
Neoavian	NEA	56	831,511.32	81.48	96,145.84	111.70	3440	1554.45
Passerea	PAS	89	840,780.94	82.39	85,230.40	100.05	3464	1731.24
Telluraves & Aequornithia & Gruae	TAG	75	886,361.18	86.86	100,620.83	100.05	3235	2572.30
Telluraves & Aequornithia	TAE	68	916,263.77	89.79	104,425.83	100.05	2979	2572.30
Telluraves	TEL	53	957,749.67	93.86	109,264.85	109.58	2424	3779.46
Eufalconimorphae	EUF	46	981,131.47	96.15	112,969.25	109.58	1775	5889.26
Psittacopasserae	PSI	65	986,045.69	96.63	102,299.10	109.58	1698	7032.14
Passeriformes	PAE	64	996,905.97	97.69	154,087.81	100.05	1435	8844.43
Passeri	PAR	50	1,002,337.45	98.22	154,800.17	113.08	1031	17,617.67
Passeroidea & Paroidea	PPA	54	1,005,719.08	98.56	117,574.56	113.08	981	17,617.67
Passeroidea	PAO	49	1,009,242.62	98.90	118,597.80	314.94	792	31,490.79
Estrildidae & Thraupidae & Fringillidae	ETF	51	1,011,702.12	99.14	155,663.35	287.41	689	31,490.79

^a^Percentage of sequence coverage against the zebra finch genome (1,020,453,418 bp)

Ancestors’ chromosome structure reconstructions generated at 100 Kbp resolution for syntenic fragments (SFs) (see reference genome selection and SF resolution selection criteria in Additional file 1: Supplementary Information) resulted in RACFs for the Avian ancestor and going through Neognathae, Neoavian, landbirds and Passeriformes ancestors to Estrildidae & Thraupidae & Fringillidae ancestor (Fig. 1, Table 1). The number of RACFs ranged from 46 (Eufalconimorphae ancestor) to 89 (Passerea ancestor) covering 78–99% of the reference (zebra finch) genome (Table 1). A lower fragmentation (no. RACFs ≤ 56) was observed for those ancestors for which both sides of the speciation node contained chromosome- or scaffold-level assemblies with N50 > 9 Mbp (ETF, PAO, PPA, PAR, EUF, TEL, NEA and NEO ancestors; Table 1) except for Psittacopasserae (PSI) reconstruction which contained 65 RACFs.

### Avian ancestors’ chromosomes

**Fig. 2:**
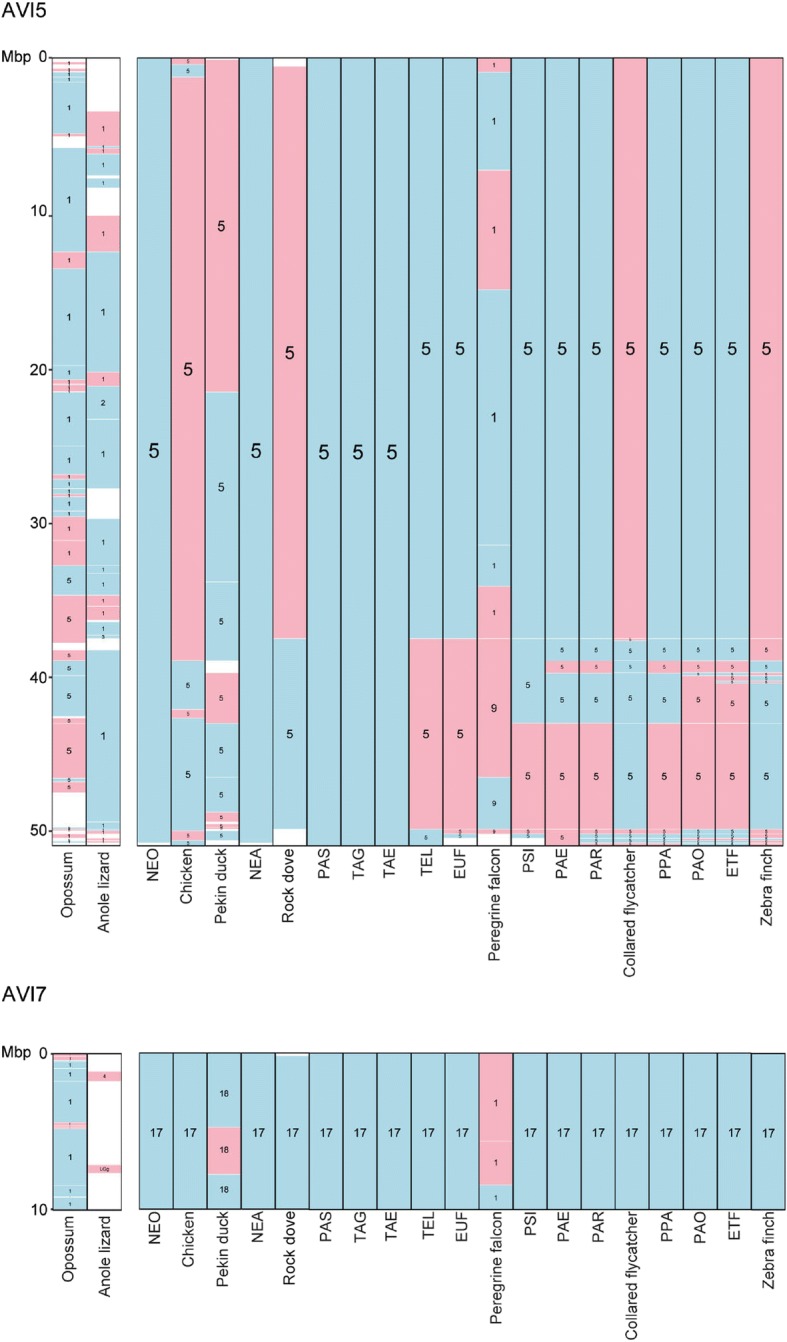
Avian ancestor chromosome 5 and 17 visualisations on the Evolution Highway comparative chromosome browser. Blue and pink blocks define syntenic fragments, in “+” and “-” orientation against the Avian ancestor chromosomes, respectively. Numbers within blocks depict chromosome numbers in each of the reconstructed ancestors and extant descendant and outgroup species assembled to chromosome level

Considering that avian karyotypes are characterised by a low number of interchromosomal changes, with exceptions limited to very few avian lineages, such as Falconiformes and Psittaciformes [2, 14–16], we ordered RACFs along chromosomes using both information from the outgroup genomes and other, more evolutionary recent ancestor RACFs. We therefore reconstructed an Avian ancestor karyotype comprising 27 autosomes that are homeologous to zebra finch chromosomes 1–28, 4A (except chromosomes 16 and 25) and Z. We named the Avian ancestor chromosomes accordingly to their zebra finch homeologues. The Neognathae and Neoavian ancestors’ karyotypes comprise 27 autosomes (1–28 and 4A; except 16 and 25), while the remaining ancestors’ karyotypes contain 28 autosomes (1–28, 1A and 4A; except 16 and 25). Due to the fragmented state of the RACFs found on the zebra finch Z chromosome, this chromosome is presented in three to seven fragments in most reconstructed ancestors. The comparative visualisation of all reconstructed ancestor karyotypes, descendant species and outgroups against the Avian ancestor chromosomes is available from the Evolution Highway (EH) comparative chromosome browser under the reference genome name “Avian:Ancestor:CHRS” [17] and Additional file 2: Figure S1; subset shown in Fig. 2).

### Evolutionary history of the Avian ancestor chromosomes

**Fig. 3:**
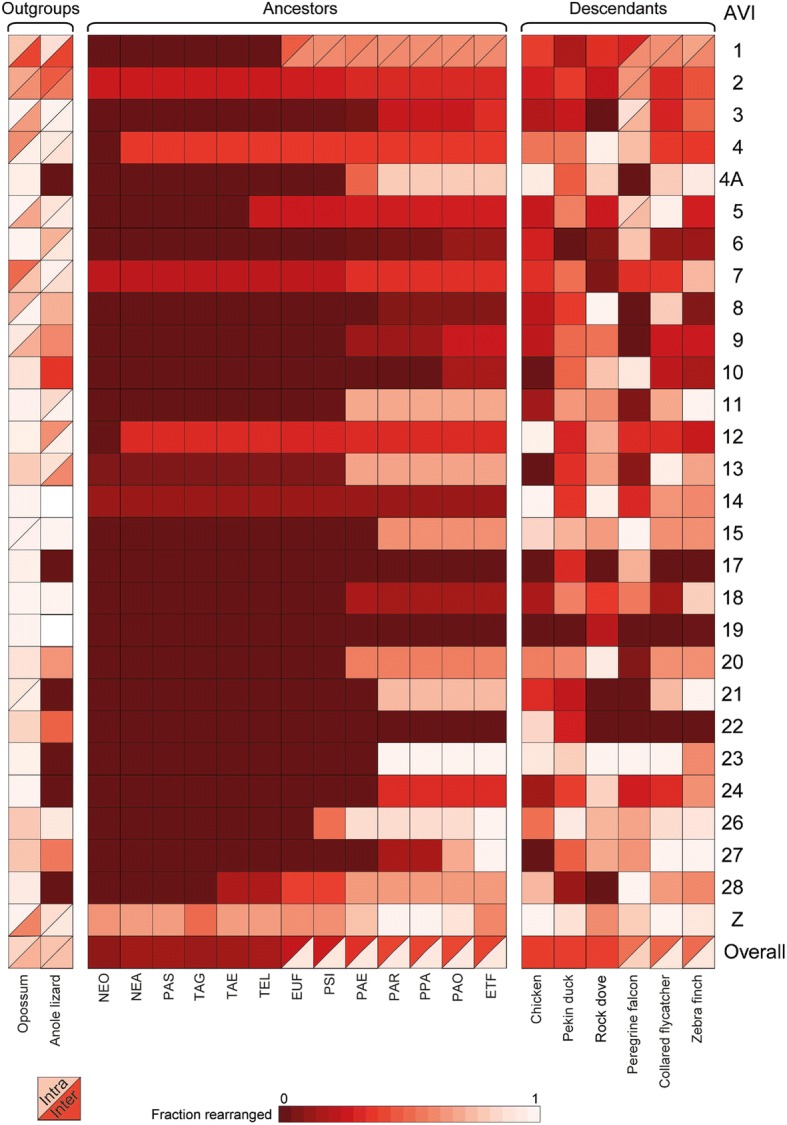
Summary visualisation of rearrangements of Avian ancestral chromosomes in chromosomes of reconstructed ancestors, and extant descendant and outgroup species. Solid red-brown squares indicate Avian chromosomes that were maintained as a single synteny block (either as a single chromosome or attached to another Avian ancestor chromosome), with shades of the colour indicating the fraction of the chromosome affected by intrachromosomal rearrangements (lightest shade is most affected). Split blocks demarcate Avian chromosomes that were affected by interchromosomal rearrangements. Upper triangles show the fraction of the chromosome affected by additional intrachromosomal rearrangements and lower triangles the fraction affected by interchromosomal changes. Acronyms for names of reconstructed ancestors correspond to their full names as shown in Table 1

Comparison of descendant ancestral karyotypes with the Avian ancestor revealed only one interchromosomal change, the fission of Avian ancestor chromosome (AVI) 1 in two chromosomes in the Eufalconimorphae ancestor (common ancestor of falcons, parrots and Passeriformes) also found in all its descendants (descendant chromosomes 1 and 1A; Fig. 3). All other changes were intrachromosomal, mostly simple inversions with a few complex rearrangements likely to result from a series of inversions affecting the same chromosome segments, e.g. on the AVI2 (38.00–75.00 Mbp [17] and Additional file 2: Figure S1).

The Neognathae ancestor karyotype was similar to the Avian ancestor one, with five chromosomes (AVI2, 7, 13, 14 and Z) affected by rearrangements (Fig. 3). The largest number of rearrangements was found in chromosome Z. The Neoavian ancestor had two more chromosomes rearranged (AVI4 and 12) and additional inversions in chromosome Z (Fig. 3). The Telluraves & Aequornithia & Gruae to Psittacopasserae lineage was characterised by a low number of changes detected only in AVI1, 5, 26 and 28. In contrast, the Passeriformes ancestor had multiple additional chromosomes affected (AVI4A, 9, 11, 18 and 20; Fig. 3). This elevated rate was maintained in the Passeri ancestor affecting chromosomes AVI3, 8, 15, 21, 23, 24 and 27 (Fig. 3). As a result, only three Avian ancestor chromosomes (AVI17, 19 and 22) were found intact in all reconstructed ancestors implying their maintenance for ~ 92 MY of avian evolution (Fig. 3).

**Fig. 4:**
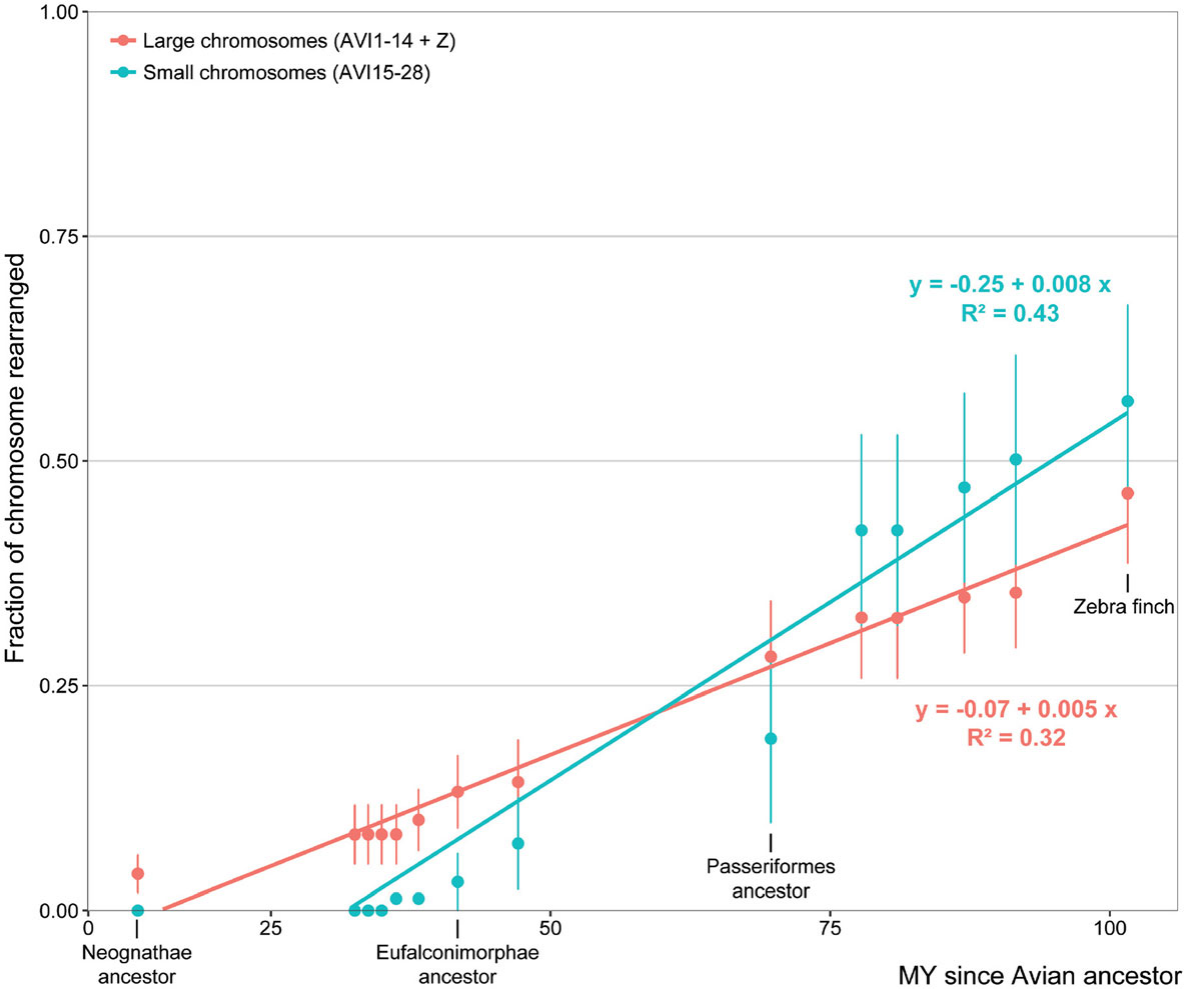
Association between the fraction of chromosome rearranged and evolutionary time for larger (AVI1–14 + Z) and smaller Avian ancestor chromosomes (AVI15–28). Blue and orange lines depict linear regressions for smaller and larger Avian ancestor chromosomes, respectively

We observed that larger chromosomes (AVI1–14 + Z) were changing in earlier ancestors (starting as early as in the Neognathae ancestor 89 MYA; Fig. 3) than smaller chromosomes (AVI15–28) (Fig. 3; Fig. 4), except for AVI28 that rearranged as early as in the Telluraves & Aequornithia ancestor (65 MYA; Fig. 3). AVI15–27 were all found intact up to the Psittacopasserae ancestor (55 MYA; Fig. 3) with > 70% of them first rearranged in the Passeri ancestral karyotype (24 MYA; Fig. 3). Seventeen Avian ancestor chromosomes (60%) were found on a single Anole lizard chromosome, of which six chromosomes (AVI4A, 17, 21, 23, 24 and 28) had no noticeable intrachromosomal changes, suggesting that these syntenies date back to the Diapsid ancestor (~ 280 MYA; Fig. 3) [18]. Amongst the extant genomes, chicken and peregrine falcon had the largest number of intact Avian ancestor chromosomes (maintained as synteny blocks that could then be fused with other chromosomes, e.g. in the peregrine falcon) with five chromosomes detected in chicken and six in peregrine falcon (Fig. 3). Contrariwise, Pekin duck had only two intact Avian ancestor chromosomes (Fig. 3).

In fact, our data suggest that small chromosomes (AVI15–28) tend to be affected by rearrangements at faster rates than larger chromosomes (AVI1–14 and Z) but in more recent ancestors (*p* value < 0.001; Fig. 4). This difference in the evolutionary history of smaller and larger chromosomes is detected independently of the tree topology as it was observed for every phylogenetic tree used to generate DESCHRAMBLER reconstructions (Additional file 1: Figure S2 and S3).

### Rates of chromosome rearrangements in avian genome evolution

To estimate and compare rates of chromosome rearrangement during avian evolution in the lineage leading to zebra finch, we calculated the number of evolutionary breakpoint regions (EBRs) for each branch of the avian phylogenetic tree (Fig. 1). To minimise the influence of potential errors introduced while merging RACFs into ancestor chromosomes, we only counted EBRs located within the original RACFs. We detected a total of 201 EBRs occurring during the ~ 100 MY of avian evolution from the Avian ancestor to zebra finch. The average rearrangement rate was estimated as 2.01 EBRs/MY. We observed that different avian lineages evolved at different rates. The Neoavian to Passerea, Telluraves to Eufalconimorphae, Passeriformes to Passeri, Estrildidae & Thraupidae & Fringillidae to zebra finch branches had rearrangement rates significantly higher than the average (> 3.5 EBRs/MY; FDR-corrected *p* value < 0.006; Fig. 1; Additional file 1: Table S9). The opposite trend was observed for Neognathae to Neoavian, Passerea to Telluraves & Aequornithia, Passeri to Passeroidea & Paroidea branches, which had genome rearrangement rates significantly lower than the average (< 1 EBR/MY; FDR-corrected *p* value < 0.03; Fig. 1; Additional file 1: Table S9). By definition, the rates of rearrangements were dependent on the branch lengths of the phylogenetic trees used in the reconstruction and therefore need to be considered with caution if the TENT tree branch lengths are doubted (Additional file 1: Table S10 and S11). However, a significantly increased rate of rearrangements after the Neoavian ancestor was supported by all the phylogenies. A low rate before the Telluraves ancestor and a high rate after the Passeriformes ancestor were supported by both the TENT [13] and Prum [19] topologies.

The genome rearrangements in man and mouse (GRIMM) webserver [20] allowed us to detect types and number of chromosomal rearrangements that have occurred between the Avian, the least fragmented Eufalconimorphae ancestor (no. RACFs = 46), the Passeriformes ancestor, the zebra finch and the chicken. As mentioned previously, only one interchromosomal rearrangement was observed, which corresponds to the fission of the AVI1 to form Eufalconimorphae ancestor chromosomes (EUF) 1 and 1A. The remaining rearrangements were chromosomal inversions. Consistent with the counts of EBRs, we observed an increased rate of chromosomal inversions (number of inversions per MY) for the ancestors phylogenetically closer to the zebra finch. From the Avian to the Eufalconimorphae ancestor, the rate of inversions was 0.77 inversions/MY; from Eufalconimorphae to Passeriformes, it increased to 1.64 inversions/MY; and from Passeriformes to zebra finch, we observed the highest rate of 2.58 inversions/MY. The number of inversions detected in our reconstruction in the five largest Avian ancestor chromosomes (AVI1–5; *N* = 59) and their zebra finch homeologues was consistent with the number reported by Romanov and colleagues (*N* = 54) [12]. In contrast, the number of inversions detected between AVI1-5 and chicken chromosomes was twofold higher using our reconstructions (*N* = 53) than that reported by Romanov and colleagues (*N* = 22) [12].

### EBR distribution in Avian ancestor chromosomes

It has been proposed that rearrangements in avian microchromosomes are rare and these chromosomes represent highly conserved blocks of synteny [12]. To test if this hypothesis holds for the lineage leading to zebra finch, we estimated distributions of EBRs in the Avian ancestor chromosomes detected from all reconstructed ancestors and the zebra finch genome.

We first compared EBR densities between chromosomes. We observed that microchromosomes AVI26, 27 and 28 had three-fold higher EBR density (> 1.4 EBR/Mbp; Table 2) than the genome-wide average (0.48 EBR/Mbp; FDR-corrected *p* value = 1.73E-07; Table 2). Distinctively, AVI2, 3, 6, 8, 9 and 10 (all macrochromosomes) had EBR densities up to eightfold lower than average (FDR-corrected *p* value < 0.05; Table 2). Consistent with our chromosome evolution analysis (Fig. 3), the Avian ancestor microchromosomes 17 and 22 contained no EBRs suggesting that they were maintained intact during the ~ 100 MY of avian evolution up to the zebra finch. Avian ancestor chromosome 19 also found intact in all reconstructed ancestors contained a single 107.5 Kbp inversion in the zebra finch genome. A similar pattern was detected when we compared the difference in the number of observed and expected EBRs (if the EBRs would be distributed uniformly across the Avian ancestor chromosomes; see Additional file 1: Supplementary Information).

**Table 2:** EBR distribution and fraction within genes, CNEs and TEs for each Avian ancestor chromosome

Avian chr.	Length (Mbp)	Chromosome fraction within			EBRs per Mbp	Average EBR distance (Mbp)
		Genes	TEs	CNEs		
1	151.05	0.51*	0.08	0.11	0.20	4.72*
2	126.80	0.46*	0.08	0.11	0.18*	6.04*
3	89.44	0.51*	0.07	0.11	0.14*	5.59*
4	56.83	0.46*	0.06*	0.09*	0.28	3.34
4A	16.17	0.44*	0.06*	0.12	0.37	2.31
5	51.05	0.54*	0.06*	0.11	0.24	3.93
6	30.27	0.55	0.05*	0.11	0.13*	6.06*
7	32.92	0.54*	0.04*	0.13*	0.24	3.66
8	21.98	0.50*	0.04*	0.14*	0.09*	7.33*
9	22.93	0.46*	0.05*	0.10	0.13*	5.73*
10	16.99	0.60	0.04*	0.16*	0.06*	8.55*
11	17.39	0.42*	0.05*	0.17*	0.46	1.93*
12	17.26	0.63	0.05*	0.14*	0.23	3.45
13	13.84	0.63	0.05*	0.14*	0.29	2.77
14	11.98	0.62	0.06*	0.11	0.34	2.40
15	11.73	0.60	0.06*	0.12	0.26	2.93
17	9.66	0.60	0.08	0.13*	0.00*	NA
18	8.79	0.64	0.07	0.11	0.57	1.46*
19	8.90	0.66*	0.07	0.12	0.22	2.97
20	10.91	0.59	0.09	0.14*	0.28	3.64
21	4.15	0.82*	0.06*	0.11	0.72	1.04*
22	2.03	0.64	0.14*	0.10	0.00*	NA
23	2.97	0.71*	0.18*	0.09	0.67	0.99*
24	4.57	0.66*	0.15*	0.01*	0.66	1.14*
26	2.91	0.69*	0.15*	0.08*	1.72*	0.48*
27	2.10	1.00*	0.13*	0.05*	1.43*	0.52*
28	2.63	0.74*	0.14*	0.06*	3.05*	0.38*
Z	42.67	0.67*	0.17*	0.05*	0.61	1.64*
Average	–	0.60	0.08	0.11	0.48	3.27

*Statistical significance compared to the average across all chromosomes (FDR-corrected *p* value < 0.05)

We also tested differences in EBR density by averaging the distance between EBRs and between the last/first EBR end/start of the chromosomes. We observed that AVI26, 27 and 28 had average distances between EBRs significantly lower than the genome-wide average (FDR-corrected *p* value = 4.16E−05; Table 2), in agreement with their higher than average EBR density per Mbp. We also observed that the chromosomes with a lower EBR density per Mbp had a higher than average distance between EBRs (FDR corrected *p* value = 4.59E−06; Table 2).

### EBR distribution and association with DNA sequence features

Several DNA sequence features were previously found associated with positions of EBRs implying that EBRs are “hotspots” of chromosome evolution, gene birth and death and changes in gene regulation [7, 9, 21, 22]. A distinct feature of avian genomes is a strong negative association of EBRs with DNA conserved non-coding elements (CNEs) with EBRs being found in CNE-sparse regions [23]. Most previous studies of EBRs, however, have focused on pairwise comparisons of extant genome assemblies. These studies potentially suffer from misidentification of EBRs some of which could be assembly artefacts. To investigate what DNA features would be associated with the distribution of EBRs in reconstructed Avian ancestor chromosomes, we tested the association between avian CNEs, zebra finch transposable elements (TEs) and chicken gene content (due to a more complete annotation than exists for zebra finch) with EBR densities on Avian ancestor chromosomes.

**Fig. 5:**
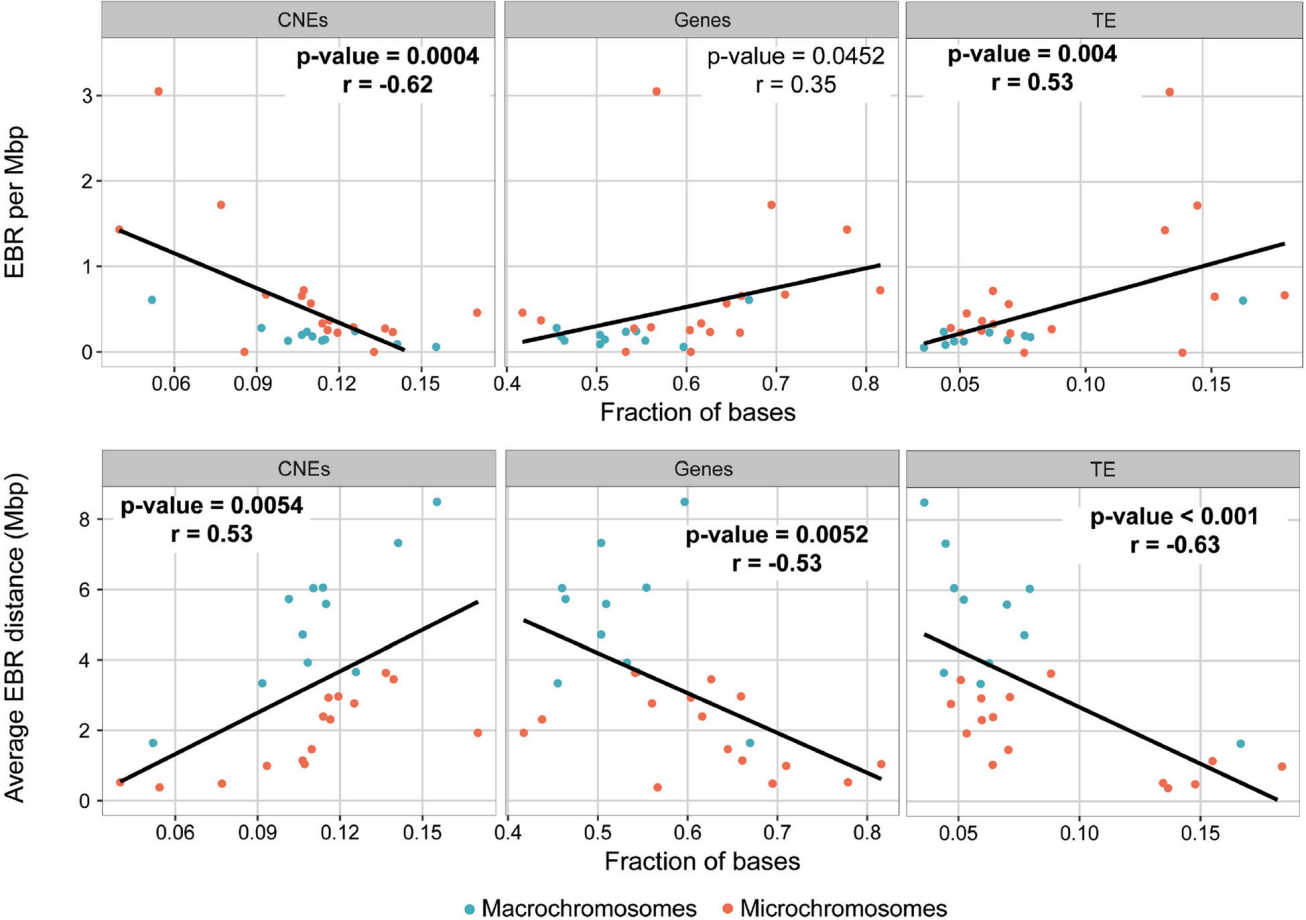
Correlation between the fraction of bases within CNEs, TEs, genes and measurements of EBRs distribution (EBRs per Mbp and average EBR distance) for Avian ancestor chromosomes. The black line shows linear correlation, and *r* and *p* values show the Pearson correlation. Bold font depicts significant correlations. Blue and orange circles depict macro- (length ≥ 20 Mbp in the zebra finch genome) and microchromosomes (length < 20 Mbp), respectively

We observed a moderately strong negative correlation between the fraction of bases within CNEs for each Avian ancestor chromosome and the corresponding EBR density (EBR/Mbp) (*p* value < 0.01; *r* = − 0.62; Fig. 5). The opposite trend was found for the average distance between EBRs on the chromosome, which presented a direct association with the fraction of bases in CNEs on Avian ancestor chromosomes (*p* value = 0.005; *r* = 0.53; Fig. 5). The same correlation pattern was observed for the difference between the observed and expected number of EBRs (Additional file 1: Figure S4). We also noticed a negative moderate correlation between average EBR distance on Avian ancestor chromosomes with the fraction of bases within genes on the same chromosomes (*p* value = 0.005; *r* = − 0.53; Fig. 5). We observed a moderately positive correlation between TE content and EBR density (EBR/Mbp) (*p* value < 0.01; *r* = 0.53; Fig. 5) and a moderately strong negative correlation when considering the average distance between EBRs on chromosomes (*p* value < 0.001; *r* = − 0.63; Fig. 5).

### Are AVI17, 19 and 22 ancestral homologous synteny blocks?

Farré and colleagues demonstrated that multispecies homologous synteny blocks (msHSBs) maintained for millions of years of avian evolution without significant rearrangements are enriched for CNEs and genes, while lineage-specific EBRs flanking msHSBs are enriched for lineage-specific TEs [21]. Remarkable conservation of AVI17, 19 and 22 in all reconstructed avian ancestors and most extant avian genomes raises a question if these chromosomes behave as individual msHSBs maintained as microchromosomes. To test this hypothesis, we compared avian CNE, TE and gene fractions in these chromosomes with those of long msHSBs (> 1.5 Mbp) identified across all the reconstructed ancestral chromosomes and extant species. Intact chromosomes present significantly different densities of all the tested features when compared with either long msHSBs, the rest of the genome not found in long msHSBs or the average across the genome (*p* values < 2E−06). Nonetheless, we observed that the density of base pairs from CNEs in the intact chromosomes combined (0.10; Table 3) is more similar to that of the long msHSBs (0.09; Table 3) than other regions of the genome. We, however, observed that combined intact chromosomes exhibit the highest base pair TE density (0.064 versus 0.054 in msHSBs; Table 3) and the highest base pair gene density (0.55 versus 0.44 in msHSBs; Table 3).

**Table 3:** Fraction of bases within genes, TEs and CNEs in Avian ancestor intact chromosomes, msHSBs > 1.5 Mbp and the rest of the genome

	Average fraction of bases within		
	Genes	TEs	CNEs
Intact chromosomes	0.5482	0.0644	0.1009
msHSB > 1.5 Mbp	0.4365	0.0536	0.0900
Rest of the genome	0.4367	0.0554	0.0884
Genome	0.4395	0.0551	0.0893

### Gene ontology enrichment analysis of AVI17, 19 and 22

**Fig. 6:**
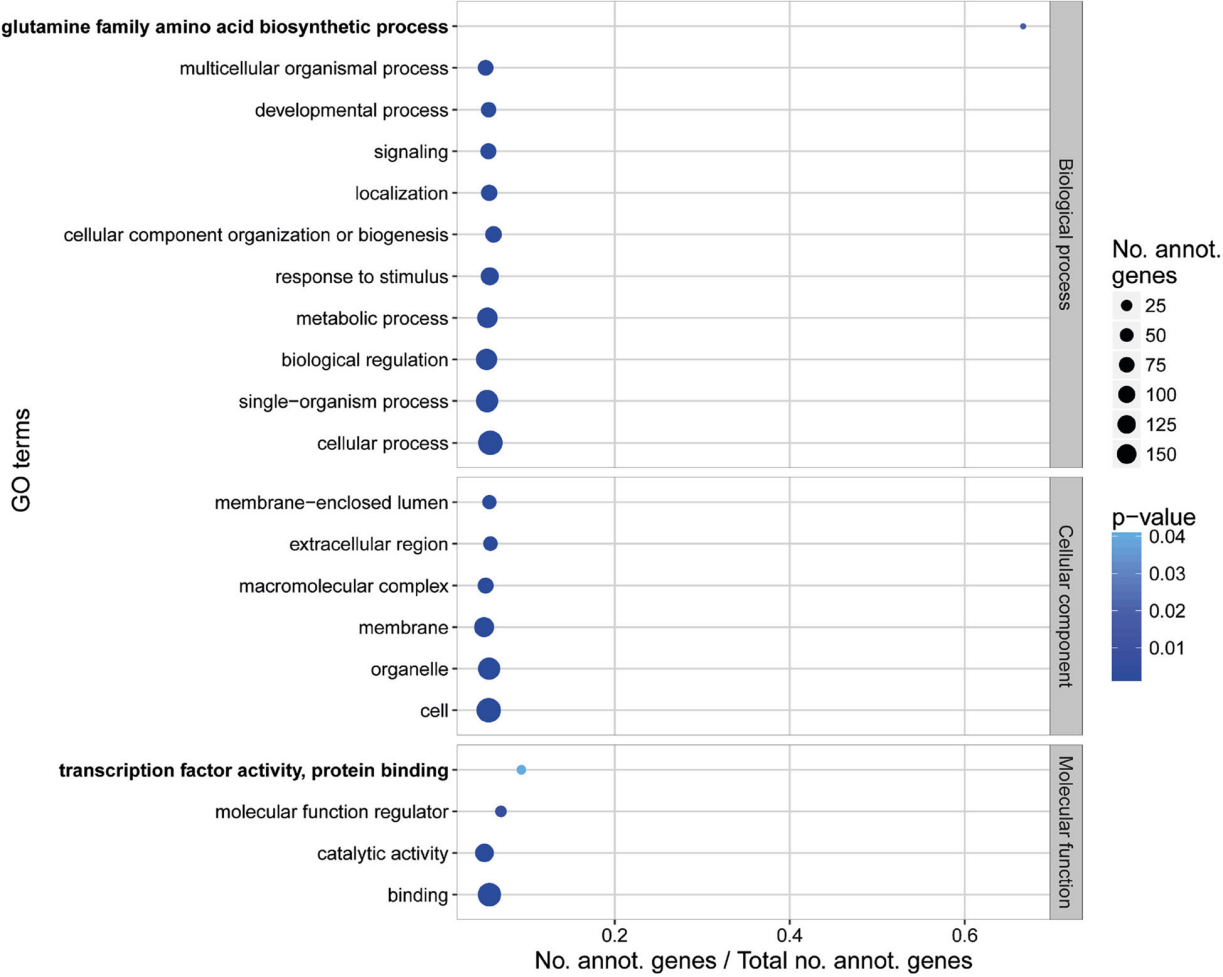
GO terms enriched in Avian ancestor chromosomes 17, 19 and 22 (*p* value < 0.05; FDR < 5%). Bubble size depicts the number of genes annotated in each GO term. Bubble shade represents the *p* value with darker shades for lower *p* values. The *x*-axis shows the ratio of genes annotated for each GO term in the analysed list versus the background list. GO terms unique for intact chromosomes are depicted in bold

Previous studies have demonstrated that HSBs maintained in amniote [24] and avian evolution [21] are enriched for categories of genes related to development and ancestral phenotypes. To test if there are functional categories overrepresented in intact Avian ancestor chromosomes we performed gene ontology (GO) enrichment analysis in AVI17, 19 and 22 and each of the non-intact Avian ancestor chromosomes. We observed an enrichment for genes related to, amongst others, *developmental process*, *cellular component organisation and biogenesis* and *molecular function regulator* in both intact and non-intact Avian ancestor chromosomes. Interestingly, in addition to GO terms enriched also in other non-intact chromosomes, including the highly rearranged michrochromosomes AVI26, 27 and 28 (Additional file 1: Figure S5), genes annotated with the GO terms *glutamine family amino acid biosynthetic process* and *transcription factor activity, protein binding* were found enriched in the intact chromosomes only (Fig. 6).

## Discussion

Using a combination of chromosome- and scaffold-level genome assemblies, we reconstructed, for the first time, the most likely chromosome structure of avian ancestors at 14 key phylogenetic nodes in the Passeriformes lineage leading to the zebra finch. None of these ancestors was reconstructed previously to the level of detail and coverage of the reference genome presented in this work. The reconstructed karyotypes allowed tracing differences in patterns of structural evolution between larger and smaller avian chromosomes for ~ 100 MY of evolution, as well as the identification of structurally stable microchromosomes and features of avian genomes that are associated with the conservation of chromosomes in evolution.

Contrary to previous studies, in which the proposed ancestral Avian karyotype included only macrochromosomes [2, 12], all the 14 reconstructions presented herein include microchromosomes orthologous to zebra finch chromosomes 11 to 28 (except 16 and 25). Our results demonstrate that smaller chromosomes (AVI15–27) become affected by rearrangements later in evolution than larger chromosomes, which started changing structurally as early as in the Neognathae ancestor (89 MYA). The reasons behind this phenomenon remain unclear; however, the data suggest that selection against the fixation of rearrangements could be responsible for the maintenance of the structural integrity of these chromosomes up to the Psittacopasserae (55 MYA) or even Passeriformes (32 MYA) ancestors. Due to their higher recombination rates (twice as high as in macrochromosomes and five times higher than in mammalian chromosomes), the requirement of at least one chiasma in each chromosome pair for correct segregation during meiosis [25, 26], and a positive association between the recombination rates and rearrangements in birds [27], microchromosomes are expected to have (and most of them demonstrate in extant genomes) higher rearrangement rates than larger chromosomes. Previous studies confirmed that microchromosomes were present as individual chromosomes as early as in the tetrapods’ ancestor [18, 25] and were not parts of larger chromosomes, therefore, could not escape the need for a chiasma in meiosis. The likely reason, therefore, why microchromosomes stayed intact for ~ 100 MY years of avian evolution could be the purifying selection against fixation of rearrangements affecting these chromosomes. Another possibility is that transposable elements (TEs) or other repetitive sequences could have been expanded in the Psittacopasserae lineage starting about 47 MYA providing more oportunities for non-allelic recombination in microchromosomes. This option implies that in the earlier ancestror genomes (> 47 MYA) repetitive elements were distributed differently between smaller and larger chromosomes (the later started rearranging in the Neognanthae ancestor). Our results demonstrate that zebra finch TE density translated to the Avian ancestor chromosomes indeed correlates with EBRs distribution along these chromosomes in the lineage leading to zebra finch. Therefore, it is possible that densities of TEs and CNEs acted in opposite directions: TEs increased oppportunities for aberrations in germ cells, while the presence of CNEs could have caused selection to disregard these changes. Further support to this hypothesis is provided by the fact that evolutionary stable AVI17, 19 and 22 (maintained intact in all reconstructed ancestors and majority of extant genomes) combined possess a significantly higher fraction of both TEs and genes than the rest of the genome suggesting that under no selection scenario, they should have accumulated internal rearrangements at the same rate as other chromosomes. Our data imply that the selection pressure has decreased about 47 MYA with 50% of microchromosomes rearranged in the Psittacopasserae ancestor and additional ones in the Passeri ancestor coinciding with the burst of diversification of Passeriformes [28] and reported TE expansion in passerine ancestors [29]. It is tempting to speculate that changes in these chromosomes could have an important role in the generation of phenotypic diversity of extant Passeriformes by forming new regulatory networks (e.g. due to changes of positions of CNEs in chromosomes). An analogous burst of chromosomal rearrangements in mammals was observed in gibbons (also accompanied with activity of TEs), but it involved interchromosomal changes in a single species [30] while in birds the karyotypes were maintained in the majority of clades.

Multiple studies demonstrated that evolutionary breakpoint regions are found in chromosomal intervals enriched for TEs and genes, but sparse in CNEs that often play gene-regulatory or structural roles in the cell [7, 9, 21–23]. The fact that the density of descendant ancestral genome and zebra finch EBRs was inversely correlated with the density of avian CNEs on the Avian ancestor chromosomes, suggests that a higher CNE fraction found in some chromosomes could account for the evolutionary stability of these chromosomes, likely by preventing fixation of germ cell chromosomal aberrations. This finding is supported by our earlier discoveries that CNEs located near genes with development-related roles in avian and other reptilian phenotypes are likely to contribute to the formation of avian multispecies homologous synteny blocks, because they contained novel regulatory elements (e.g. transcription factor binding sites [21]) and that avian EBRs are found in CNE-sparse genome intervals [23]. Our results, however, suggest that this mechanism could be extended to complete chromosomes, rather than individual homologous synteny blocks or EBRs within chromosomes. The difference in rearrangement rates between larger and smaller chromosomes, the fact that all three evolutionary stable Avian ancestor chromosomes found in this study were microchromosomes, and their sequence feature content, suggest that these chromosomes might behave as large homologous synteny blocks and their further breakage/rearrangements would likely have significant biological effects. Also, the fact that the TE density in Avian ancestor chromosomes correlates with their EBR density supports the role of TEs in the genome evolution of birds by promoting DNA breakage and/or joining [31, 32]. The location in these evolutionary stable chromosomes of many genes essential for the correct development of embryos as indicated by the GO analysis is supporting this hypothesis as well, as changes in the organisation of these genomic regions could disturb gene regulation and have deleterious functional effects, leading to their removal by purifying selection. Rearrangements found in these three chromosomes in a small number of extant genomes are therefore surprising suggesting that either these changes might had important biological implications or are assembly artefacts to be fixed in future genome assembly improvement experiments.

High variability of genome-wide chromosomal rearrangement rates between avian phylogenetic clades reported here agrees with the data reported by Zhang and colleagues [33] with discrepancies found for the Passeriformes to Passeri and the Avian to Neognathae branches. This might be related to the higher number of chromosome-level assemblies and overall higher continuity of genomes present in our dataset, which facilitated the detection of rearrangements unidentified in the previous work or to a large number of EBRs that did not pass Zhang and colleagues’ conservative filtering threshold [33]. We also cannot exclude the possibility that the merging of reconstructed fragments to chromosomes could make the Avian ancestor chromosomes, in some cases, more structurally similar to its descendant genomes than they should be.

The number of inversions detected between the five largest Avian ancestor chromosomes (1 to 5) and their zebra finch homeologues is highly consistent between the current analysis and that of our previous studies [12]. However, the opposite is observed for the chicken where our current reconstruction allows the detection of twice as many inversions compared to what we found previously [12]. This inconsistency might be caused by an underrepresentation of avian clades in our earlier work, leading to a bias of our reconstructions to Galloanserae genome structures.

Despite the utility of the predicted ancestor genome structures to better understand avian chromosome evolution, they are not free of limitations. Due to the use of only one reference genome to define syntenic fragments, it is possible that some ancestral sequences that are not present in the reference genome, zebra finch in our case, were omitted from the reconstructions. Moreover, the predominance of scaffold-level assemblies in the descendant species results in fragmentation of predicted ancestral chromosomes. Indeed, we observed a lower number of RACFs for those ancestors to which both sides of the speciation node contained chromosome-level assemblies or scaffold-level assemblies with N50 > 9 Mbp, which reinforces the importance of having high continuity genome assemblies to facilitate the study of chromosome evolution. Another limitation is that the phylogenetic relationship of some avian clades is not well resolved as suggested by the disparities between avian phylogenetic trees proposed in different studies [13, 19, 34]. Our reconstructions, however, imply that the TENT tree topology from Jarvis and colleagues [13] was the most consistent with the underlying genomic data resulting in the least fragmented ancestral genome reconstructions compared to the other two tested trees. High agreement in RACF structures for well-established ancestral nodes shared between different trees suggests that the DESCHRAMBLER reconstructions are highly stable. Exclusion or inclusion of a small number of additional species had a small effect on the reconstruction of ancestors from ‘sister’ nodes. On the other hand, differences in estimations of branch lengths and evolutionary time of nodes between trees influenced the rates of rearrangements on individual phylogenetic nodes but had no effect on the patterns of evolutionary history of individual Avian ancestor chromosomes.

## Conclusions

In this work, the reconstruction of the Avian ancestor and 13 additional avian ancestral karyotypes offered valuable novel insights into the history and patterns of chromosome rearrangements in the avian lineage. Our finding of significant differences in evolutionary histories of micro- and macrochromosomes in the ancestral Passeriformes lineage suggests that purifying selection was likely to act supporting the structural integrity of the majority of microchromosomes for ~ 50 MY of avian evolution. Why this pattern has changed ~ 47 MY ago with only three microchromosomes maintained intact in the most recent ancestor is still unknown. It could however be hypothesised that this event relates to the high density and diversity of retrotransposons observed in extant Passerine birds which activity was previously proposed to be linked to genome instability and species diversification in birds [29, 35].

The next questions to ask are as follows: (i) if this phenomenon is limited to Passeriformes or if it is a general signature of avian evolution and (ii) if it can be related to active speciation within Passeriformes? Moreover, the ancestral karyotype reconstructions presented herein provide an excellent resource for tracing structural changes in all avian lineages and to study their influence on the biology of the extant ~ 10,000 species.

To work towards answering these questions we need more high-quality (ideally chromosome-level) assemblies for all avian species. New sequencing and mapping technologies make this feasible. However, even the best assemblies are not free from errors. Reconstruction of ancestral genomes might assist the identification of potential problematic assembly regions and the production of more accurate genome assemblies. These, in turn, would increase the accuracy of the subsequent ancestral genome reconstructions, until one would eventually have access to a comprehensive catalogue of the events that shaped extant avian genomes and reveal their implications on biology of the species.

## Methods

### Avian and outgroup genome assemblies

The chicken (*Gallus gallus*; ICGSC Gallus_gallus 4.0 [36]), zebra finch (*Taeniopygia guttata*; WUGSC 3.2.4 [37]) and turkey (*Meleagris gallopavo*; TGC Turkey_2.01 [38]) chromosome assemblies were downloaded from the UCSC Genome Browser [39]. The collared flycatcher (*Ficedula albicollis*; FicAlb1.5 [40]), peregrine falcon (*Falco peregrinus*) and rock pigeon (*Columba livia*) [23] chromosome assemblies were downloaded from NCBI. The Pekin duck (*Anas platyrhynchos*; BGI_duck_1.0 [41]) assembly was downloaded from NCBI and upgraded to chromosome level using radiation hybrid map obtained from Dr. Thomas Faraut (INRA). The hooded crow (*Corvus cornix*; Hooded_crow_genome [42]), canary (*Serinus canaria*; SCA1 [43]), Tibetan ground tit (*Pseudopodoces humilis*; PseHum1.0 [44]), golden eagle (*Aquila chrysaetos*; Aquila_chrysaetos-1.0.2 [45]) and bald eagle (*Haliaeetus leucocephalus*; Haliaeetus_leucocephalus-4.0) scaffold assemblies were obtained from NCBI. All remaining scaffold-based assemblies were downloaded from the GigaScience Database [46, 47]. Chromosome assemblies of outgroup genomes: anole lizard (*Anolis carolinensis*; AnoCar2.0 [48]) and opossum (*Monodelphis domestica*; MonDom5 [49]), the scaffolds assemblies of the Chinese alligator (*Alligator sinensis*; ASM45574v1 [50]) and the painted turtle (*Chrysemys picta*; Chrysemys_picta_bellii-3.0.1 [51]) were obtained from NCBI. General assembly statistics for each genome used are presented in Additional file 1: Table S1. Divergence times and topologies were obtained from the total evidence nucleotide tree reported by Jarvis and colleagues (2014) [13]. Clade nomenclature was based on Jarvis and colleagues [13], Suh and colleagues [52], Yuri and colleagues [53] and taxonomy from Flux webpage [54].

### Pairwise alignments

We selected zebra finch as the reference genome for the reconstruction of avian ancestors as one of the best avian genome assemblies currently available, and because DESCHRAMBLER [11] requires the reference to be a descendant species for all reconstructed ancestors. Moreover, zebra finch is a representative of Passeriformes, the avian clade with the highest number of extant species, and which species also exhibit a high phenotypic diversity. Pairwise alignments using zebra finch chromosome assembly as the reference and all other genomes as targets were generated with LastZ (version 1.02.00 [55]) using the following parameters: *C = 0 E = 30 H = 2000 K = 3000 L = 2200 O = 400*. The pairwise alignments were converted into the UCSC “chain” and “net” alignment formats with axtChain (parameters: *-minScore = 1000 -verbose = 0 -linearGap = loose* for anole lizard and opossum, and *-minScore = 1000 -verbose = 0 -linearGap = medium* for all other species) followed by chainAntiRepeat, chainSort, chainPreNet, chainNet and netSyntenic, all with default parameters [56].

### Reconstructed ancestral chromosome fragments

First, DESCHRAMBLER [11] was used to reconstruct RACFs of the Neognathae ancestor with a subset of species, as indicated in Additional file 1: Table S1. This experiment was performed at 100, 300 and 500 Kbp SFs resolution. After the selection of the best SF resolution for avian ancestral chromosomes reconstruction (100 Kbp; see resolution selection criteria in Additional file 1: Supplementary Information), DESCHRAMBLER was run with the full set of species to generate RACFs for all ancestors leading to zebra finch lineage, starting with the Avian ancestor.

After the resolution of the analysis was established, we performed test DESCHRAMBLER reconstructions using three phylogenetic trees. As the topology at the base of Neoaves is not completely resolved, we chose (1) a TENT tree from Jarvis and colleagues as a tree with the largest number of loci used [13], (2) a tree from Reddy and colleagues with a large taxon sampling and including both coding and non-cording loci in the analysis [34] and (3) a tree from Prum and colleagues due to a large taxon sampling [19]. These trees disagree in placement of some species on the phylogenetic nodes. DESCHRAMBLER produced the least fragmented reconstructions using the Jarvis and colleagues (2014) tree. RACFs reconstructed using different trees were compared and found to be highly consistent (see Additional file 1: Supplementary Information) with all trees resulting in a similar number of EBRs. With the least fragmented reconstructions, the TENT tree results were chosen to be presented in the main text of the paper with other reconstructions being used to support the results or to indicate differences.

### Detection of EBRs and chromosome rearrangements

We detected EBRs relative to the Avian ancestor in all other ancestors’ RACFs and the zebra finch using a previously published methodology [21]. Breakpoint rates (EBRs/MY) for each branch leading to zebra finch were calculated dividing the number of detected EBRs by the length of the branch (in MY as in Jarvis and colleagues (2014) [13]). Differences in breakpoint rates compared to the average of all branches were tested as previously described [11]. Differences in cumulative fractions of smaller and larger Avian ancestor chromosomes rearranged in reconstructed ancestors were tested by analysis of covariance in R (version 3.4.2 [57]).

We used the genome rearrangements in man and mouse (GRIMM) webserver [20] to predict the minimum number and the type of chromosomal rearrangements distinguishing the Avian ancestor chromosomes structure from those of the Eufalconimorphae and Passeriformes ancestors, the zebra finch and chicken genomes.

### Avian ancestors’ chromosomes

The number of RACFs reconstructed by DESCHRAMBLER was higher than the number of Avian ancestor chromosomes previously proposed based on FISH experiments [2]. This fragmentation is mostly due to the predominance of scaffold-level assemblies for the descendant species, resulting in a reduction of adjacency support. To reduce the fragmentation of the reconstructed avian ancestors’ genomes, we ordered RACFs by connecting RACFs which adjacency was supported by outgroup genomes or other, phylogenetically close and less fragmented (at the same position) ancestors. Specifically, for the Avian ancestor, we first merged those RACFs which adjacencies were supported (spanned) by an outgroup chromosome or scaffold. For the remaining Avian RACFs adjacencies with no support from outgroup genomes and the other reconstructed ancestors, we merged RACFs which adjacency was supported by other ancestor RACF, assuming that no rearrangement occurred between the target and the descendant ancestor in between RACFs. For each RACF adjacency, we used the support from the spanning RACF belonging to ancestors successively more distant. That is, we used first the support from the closest ancestor (e.g. Neognathae for the Avian ancestor) and successively more recent ancestors on the avian phylogenetic tree.

### Fraction of rearranged avian ancestor chromosomes

For each Avian ancestor chromosomes, we calculated its fraction involved in intrachromosomal rearrangements on the other ancestors and extant descendant and outgroup species. We first established the ancestral state chromosome orientation by detecting which orientation would imply the least number of rearrangements. Then, we calculated its fraction involved in rearrangements by dividing the non-ancestral orientation by the cumulative length of the blocks mapped into that chromosome.

The fraction of the Avian ancestor chromosomes affected by interchromosomal rearrangements was calculated by dividing the cumulative length of the blocks of each target chromosome by the total length of the target blocks mapped into the Avian ancestor chromosome. The represented fraction corresponds to the lowest obtained value.

### EBR rates and DNA sequence feature associations on Avian ancestor chromosomes and HSBs

We measured EBR density and distribution for the Avian ancestor chromosomes using the number of EBRs identified between the Avian ancestor and zebra finch. These measurements were obtained as the number of EBRs per Mbp and the average distance between EBRs. Differences between chromosomes for each of the analysed features were tested as previously reported [11].

Avian conserved non-coding elements (CNEs) were obtained from Farré and colleagues (2016) [21]. Chicken gene (version of 27/04/2014) and zebra finch repetitive sequence (version of 08/05/2014) annotations were downloaded from the UCSC genome browser [58]. We calculated the density of each of these features (CNEs, genes and TEs) for each Avian ancestor chromosome. The association between each sequence feature and chromosome-specific EBR density and distribution was tested using the Pearson’s correlation coefficient.

Avian ancestor chromosomes were divided into 1 Kbp non-overlapping windows. All intervals were assigned to either msHSBs, detected across the reconstructed ancestral chromosome and extant species genome assemblies, intact chromosomes (AVI17, 19 and 22) or intervals found in the rest of the genome. The CNE, TE and gene density were calculated for each window type using bedtools (version 2.20-1 [59]). Differences between each of these two sets for each analysed feature were tested as previously reported [23].

### Gene ontology enrichment analysis

The basic version of gene ontology (GO) annotations (version 8 April 2017) was downloaded from the GO Consortium website [60]. Sequence coordinates and Ensembl identifiers for chicken genes were obtained from Ensembl Biomart (version 74 [61]). All chicken genes located in regions included in the Avian ancestor chromosomes were used as the background list. To evaluate gene functional enrichment in the Avian ancestor chromosomes that were maintained intact during avian evolution, we assigned genes from the background list to these chromosomes. We used the GO::TermFinder Perl module [62] to detect GO terms overrepresented in our gene sets. We considered as significantly enriched the terms with *p* value < 0.05 and false discovery rate (FDR) < 5%.

## Additional files

Additional file 1: Supplementary information, tables and figures. (PDF 781 kb)

Additional file 2: Evolution highway visualisations of Avian ancestor chromosomes. (PDF 2360 kb)
