# Supplementary material for: Reconstruction of avian ancestral karyotypes reveals differences in the evolutionary history of macro- and microchromosomes
Source: Genome Biol. 2018 Oct 5;19:155. doi: 10.1186/s13059-018-1544-8 (PMC6173868; doi:10.1186/s13059-018-1544-8)
Supplement: Supplementary file 1 — Supplementary information, tables and figures. (PDF 781 kb) [file 13059_2018_1544_MOESM1_ESM.pdf]

Table of Contents

Supplementary Information

Selection of reference and descendant genomes …………………………………………... 3

Selection of resolution for syntenic fragment detection and RACF reconstructions ………… 3

EBR distribution in Avian ancestor chromosomes ……………………………………..…. 4

Supplementary Tables

Table S1: Statistics for the genome assemblies of descendant and outgroup species ……..… 6

Table S2: Statistics for the Neognathae ancestor reconstructions at different resolutions of SF detection …………………………...…………………………………………………….... 7

Table S3: Statistics of the reconstructed ancestors following Prum et al. (2015) topology (100 Kbp resolution) …………………………………...…………………………………….. 8

Table S4: Statistics of the reconstructed ancestors following Reddy et al. (2017) topology (100 Kbp resolution) ……………………………………………………………………….... 9

Table S5: Comparison between Jarvis et al. (2014) and Prum et al. (2015) ancestors with the same ingroup species ………………………….…………………………………………. 10

Table S6: Comparison between Jarvis et al. (2014) and Reddy et al. (2017) ancestors with the same ingroup species ………………………………………………………………... 10

Table S7: Comparison between Jarvis et al. (2014) and Prum et al. (2015) “sister” clades ancestors ………………………………………………………………………………. 11

Table S8: Comparison between Jarvis et al. (2014) and Reddy et al. (2017) “sister” clades ancestors ……………………...……………………...……………………………………... 11

Table S9: Number of EBRs and rearrangement rates for the reconstructed ancestral genomes following Jarvis et al. (2014) topology …………………….…………………………………. 12

Table S10: Number of EBRs and rearrangement rates for the reconstructed ancestral genomes following Prum et al. (2015) topology ……………………………….……………………….. 13

Table S11: Number of EBRs and rearrangement rates for the reconstructed ancestral genomes following Reddy et al. (2017) topology ……………………………………..…………… 14

Table S12: EBR distribution (observed-expected number of EBRs) for Avian ancestor chromosomes ……………………………..…………………………………………….... 15

Supplementary Figures

Figure S2: Association between the fraction of chromosome rearranged and evolutionary time for larger (AVI1-14 + Z) and smaller Avian ancestor chromosomes (AVI15-28) for Prum et al. (2015) topology ……………………………………..……………………… 16

Figure S3: Association between the fraction of chromosome rearranged and evolutionary time for larger (AVI1-14 + Z) and smaller Avian ancestor chromosomes (AVI15-28) for Reddy et al. (2017) topology ………………………..……………………………………...… 17

Figure S4: Correlation between the fraction of bases within CNEs, TEs, genes, and measurements of EBRs distribution (observed-expected number of EBRs) for Avian ancestor chromosomes ……………………………..…………………………………………….... 18

Figure S5: GO terms enriched on Avian ancestor chromosomes 26, 27 and 28 (p-value < 0.05; FDR < 5%) …………………..………………………………………………………… 19

Supplementary Information

Selection of reference and descendant genomes

The set of genomes used in ancestors' chromosome reconstructions included 27 avian genomes (seven chromosome- and 20 scaffold-level assemblies) and four outgroup genomes (two chromosome- and two scaffold-level assemblies: three non-avian reptiles and one mammal; Table S1). These genomes were selected according to their assembly continuity (N50 > 2 Mbp) and alignment coverage of the reference genome (zebra finch genome coverage >96%). On one hand, assembly continuity is a critical selection parameter because: a) the use of highly fragmented descendant genomes will reduce the support to predicted ancestral adjacencies and result in fragmented ancestors' karyotype reconstructions, and b) increased genome continuity will increase the chances of detection of evolutionary breakpoint regions (EBRs) flanking genome rearrangements, as EBRs located in between scaffolds in the extant species assemblies will be missed or lead to misassignment of detected EBRs on the phylogenetic tree. On the other hand, high reference genome alignment coverage assures a more complete reconstruction of ancestral karyotypes. The final set of avian species included in our reconstructions represent 15 out of 37 avian orders, a comprehensive sampling of the avian phylogenetic tree, and allowed the reconstruction of 14 ancestor chromosome structures starting with the Avian ancestor and leading to the zebra finch.

Selection of resolution for syntenic fragment detection and RACF reconstructions

We first performed the reconstruction of the Neognathae ancestor chromosome structure at three (100, 300 and 500 Kbp) syntenic fragment (SF) resolutions, to set the minimum length of SF to be included in the reconstructions. We selected the Neognathae ancestor for this experiment

as both ingroup and avian outgroup genome alignments covered >94% of the zebra finch genome. To be included in the RACFs an SF needs to be present in at least one of the outgroup genomes therefore genomes with high coverage alignments of the reference genome would result in more complete RACFs. Also, this test aimed at the establishment of the optimal resolution for avian ancestral genome reconstructions. We found that the number of RACFs obtained at 100 Kbp SF resolution was the lowest (N=62) and the reference genome coverage was the highest (79%; Table S2). At 300 Kbp resolution, RACFs covered ~46% of reference genome and the number of reconstructed RACFs was higher (N=80; Table S2). Lastly, at 500 Kbp resolution, there were 64 RACFs reconstructed but they covered just ~31% of the reference genome (Table S2). To minimise the fragmentation of the reconstructed ancestral genomes and, at the same time, maximise the coverage of the reference genome the 100 Kbp SF resolution was used for the reconstructions.

EBR distribution in Avian ancestor chromosomes

To test if EBRs were distributed uniformly across the ancestral avian chromosomes, we calculated the difference between the number of observed and expected EBRs for each Avian ancestor chromosomes. The expected number of EBRs per chromosome was calculated by multiplying the length of the chromosome (in Mbp) by the genome-wide rate of EBRs. The latter was calculated by dividing the total number of detected EBRs by the total length of the reconstructed Avian ancestor genome.

We observed that all Avian ancestor chromosomes with an EBR density significantly lower than average also possessed fewer EBRs than would be expected if the EBRs were distributed uniformly across the genome (FDR-corrected p-value <0.02; Table S12). We noted the same trend

for the chromosomes with an EBR density higher than average, that is, these chromosomes contained a significantly higher number of EBRs than would be expected from a uniform EBR distribution along the genome (FDR corrected p-value <0.03; Table S12).

Supplementary Tables

Table S1: Statistics for the genome assemblies of descendant and outgroup species.

Species	Common name	Assembly type	No. [a]	N50 (Mbp)	Total length (Gbp)	On test recons. [b]
Taeniopygia guttata	Zebra finch	Chromosome	31	-	1.02	Yes
Geospiza fortis	Medium ground finch	Scaffold	1,168	5.28	1.04	Yes
Serinus canaria	Canary	Scaffold	887	25.15	1.05	No
Pseudopodoces humilis	Tibetan ground tit	Scaffold	661	16.34	1.04	No
Corvus brachyrhynchos	American crow	Scaffold	1,156	7.08	1.08	Yes
Corvus cornix	Hooded crow	Scaffold	366	16.36	1.05	No
Ficedula albicollis	Collared flycatcher	Chromosome	30	-	1.04	Yes
Manacus vitellinus	Golden-collared manakin	Scaffold	954	2.86	1.05	Yes
Melopsittacus undulatus	Budgerigar	Scaffold	1,138	11.41	1.08	Yes
Falco peregrinus	Peregrine falcon	Chromosome	19	-	1.03	No
Aquila chrysaetos	Golden eagle	Scaffold	470	9.23	1.19	No
Haliaeetus leucocephalus	Bald eagle	Scaffold	435	9.15	1.18	No
Picoides pubescens	Downy woodpecker	Scaffold	1,944	2.12	1.15	Yes
Pygoscelis adeliae	Adélie penguin	Scaffold	819	5.23	1.21	Yes
Aptenodytes forsteri	Emperor penguin	Scaffold	682	5.08	1.25	Yes
Nipponia nippon	Crested ibis	Scaffold	1,479	5.35	1.20	Yes
Egretta garzetta	Little egret	Scaffold	1,195	3.11	1.20	Yes
Opisthocomus hoazin	Hoatzin	Scaffold	1,620	2.94	1.20	Yes
Charadrius vociferus	Killdeer	Scaffold	1,598	3.68	1.21	Yes
Cuculus canorus	Common cuckoo	Scaffold	900	2.99	1.15	Yes
Chaetura pelagica	Chimney swift	Scaffold	1,172	3.88	1.10	Yes
Calypte anna	Anna's hummingbird	Scaffold	887	4.30	1.05	Yes
Columba livia	Rock pigeon	Chromosome	29	-	0.91	No
Gallus gallus	Chicken	Chromosome	30	-	1.00	Yes
Meleagris gallopavo	Turkey	Chromosome	32	-	1.04	Yes
Anas platyrhynchos	Pekin duck	Chromosome	29	-	0.94	Yes
Struthio camelus	Ostrich	Scaffold	1,179	3.64	1.22	Yes
Alligator sinensis	Chinese alligator	Scaffold	2,452	2.19	2.26	Yes
Chrysemys picta	Painted turtle	Scaffold	3,168	7.23	2.29	Yes
Anolis carolinensis	Anole lizard	Chromosome [c]	12	-	1.08	No
Monodelphis domestica	Opossum	Chromosome	9	-	3.50	No

[a] Number of scaffolds or chromosomes in the genome assembly.

[b] Species included in Neognathae test reconstructions.

[c] Chromosomes and linkage groups in Anole lizard.

Table S2: Statistics for the Neognathae ancestor reconstructions at different resolutions of SF detection.

Resolution (Kbp)	No. RACFs	Total length RACFs (Kbp)	Coverage (%) [a]	Longest RACF (Kbp)	Shortest RACF (Kbp)	No. SFs	Longest SF (Kbp)	Shortest SF (Kbp)
100	62	802,050.27	78.60	92,542.59	108.99	3,539	1,654.18	100.01
300	80	471,160.79	46.17	47,135.00	310.97	1,018	1,700.52	300.01
500	64	319,886.64	31.35	33,091.13	508.92	456	1,700.52	501.68

[a] Percentage of sequence coverage against the zebra finch genome (1,020,453,418 bp).

Table S3: Statistics of the reconstructed ancestors following Prum et al. (2015) topology (100 Kbp resolution).

Ancestor	No. RACFs	Total length RACFs (Kbp)	Coverage (%) [a]	Longest RACF (Kbp)	Shortest RACF (Kbp)	No. SFs	Longest SF (Kbp)
**Avian**	91	791.75	77.59	90,790.86	100.10	3,494	1,554.45
**Neognathae**	60	809.30	79.31	92,682.95	109.58	3,463	1,554.45
**Neoavian**	71	835.14	81.84	96,038.89	100.01	3,463	1,554.45
**Telluraves & Aequornithia & Gruae & Cuculidae & Columbidae**	69	862.85	84.56	98,299.14	100.05	3,353	1,656.44
**Telluraves & Aequornithia & Gruae**	87	885.74	86.80	100,596.55	100.05	3,231	2,572.30
**Telluraves & Opisthocomidae**	68	940.44	92.16	99,355.40	109.58	2,693	3,128.63
**Telluraves**	58	957.78	93.86	109,237.25	109.58	2,426	3,779.46
**Eufalconimorphae & Picidae**	54	961.65	94.24	109,477.06	100.05	2,345	4,452.05
**Eufalconimorphae**	47	979.08	95.95	102,107.61	132.11	1,764	5,889.26
**Psittacopasserae**	62	985.96	96.62	102,291.88	129.62	1,698	7,032.14
**Passeriformes**	66	996.81	97.68	99,739.08	100.05	1,435	8,844.43
**Estrildidae & Thraupidae & Muscicapidae & Pipridae**	51	1,008.92	98.87	155,275.15	131.27	1,075	9,367.47
**Estrildidae & Thraupidae & Muscicapidae**	47	1,010.62	99.04	155,677.52	119.33	572	30,160.46
**Estrildidae & Thraupidae**	50	1,010.95	99.07	117,393.74	270.80	488	31,490.79

[a] Percentage of sequence coverage against the zebra finch genome (1,020,453,418 bp).

Table S4: Statistics of the reconstructed ancestors following Reddy et al. (2017) topology (100 Kbp resolution).

Ancestor	No. RACFs	Total length RACFs (Kbp)	Coverage (%) [a]	Longest RACF (Kbp)	Shortest RACF (Kbp)	No. SFs	Longest SF (Kbp)
**Avian**	80	790.90	77.50	90,900.21	108.57	3,489	1,554.45
**Neognathae**	56	806.09	78.99	92,896.56	103.58	3,443	1,554.45
**Neoavian**	57	831.31	81.46	96,363.51	111.70	3,440	1,554.45
**Passerea**	92	840.46	82.36	84,257.11	100.05	3,463	1,731.24
**Telluraves & Apodiformes & Gruae**	91	902.25	88.42	103,792.14	100.05	3,071	2,953.80
**Telluraves & Apodiformes & Opisthocomidae**	78	915.12	89.68	104,861.15	100.05	2,956	3,168.63
**Telluraves & Apodiformes**	59	933.16	91.45	148,115.42	100.05	2,754	3,779.46
**Telluraves**	52	957.29	93.81	109,264.48	109.58	2,421	3,779.46
**Eufalconimorphae**	46	981.03	96.14	112,969.25	109.58	1,774	5,889.26
**Psittacopasserae**	66	986.07	96.63	102,302.91	109.58	1,698	7,032.14
**Passeriformes**	75	996.90	97.69	97,178.76	100.05	1,435	8,844.43
**Passeri**	50	1,002.36	98.23	154,803.99	113.08	1,031	17,617.67
**Estrildidae & Thraupidae & Fringilidae & Muscicapidae & Paridae**	48	1,002.49	98.24	155,369.66	203.61	841	30,160.46
**Estrildidae & Thraupidae & Fringilidae & Muscicapidae**	42	1,007.39	98.72	155,587.03	119.33	754	30,160.46
**Estrildidae & Thraupidae & Fringilidae**	51	1,011.70	99.14	155,663.35	287.41	689	31,490.79

[a] Percentage of sequence coverage against the zebra finch genome (1,020,453,418 bp).

Table S5: Comparison between Jarvis et al. (2014) and Prum et al. (2015) ancestors with the same ingroup species.

	Jarvis adjacencies in Prum				Prum adjacencies in Jarvis			
**Ancestor name**	**No. adjacencies**	**Maintained**	**Extra**	**Inconsistent**	**No. adjacencies**	**Maintained**	**Extra**	**Inconsistent**
Psittacopasserae	142	138(97%)	3(2%)	1(1%)	140	136(97%)	3(2%)	1(1%)
Eufalconimorphae	161	154(96%)	6(4%)	1(0%)	154	153(99%)	0(0%)	1(1%)
Telluraves	171	166(97%)	4(2%)	1(1%)	170	166(98%)	2(1%)	2(1%)
Telluraves & Aequornithia & Gruae	158	149(94%)	7(5%)	2(1%)	154	153(99%)	1(1%)	0(0%)
Neoavian	162	152(94%)	8(5%)	2(1%)	157	153(97%)	3(2%)	1(1%)
Neognathae	163	159(98%)	3(2%)	1(0%)	161	159(99%)	2(1%)	0(0%)
Avian	163	159(98%)	3(2%)	1(0%)	163	158(97%)	3(2%)	2(1%)

Table S6: Comparison between Jarvis et al. (2014) and Reddy et al. (2017) ancestors with the same ingroup species.

	Jarvis adjacencies in Reddy				Reddy adjacencies in Jarvis			
**Ancestor name**	**No. adjacencies**	**Maintained**	**Extra**	**Inconsistent**	**No. adjacencies**	**Maintained**	**Extra**	**Inconsistent**
Estrildidae & Thraupidae & Fringilidae	62	61 (98%)	0 (0%)	1 (2%)	61	61 (100%)	0 (0%)	0 (0%)
Passeri	95	91 (96%)	4 (4%)	0 (0%)	96	91 (95%)	5 (5%)	0 (0%)
Passeriformes	115	111 (96%)	3 (3%)	1 (1%)	112	111 (99%)	1 (1%)	0 (0%)
Psittacopasserae	142	142 (100%)	0 (0%)	0 (0%)	143	142 (99%)	1 (1%)	0 (0%)
Eufalconimorphae	161	160 (99%)	1 (1%)	0 (0%)	159	158 (99%)	1 (1%)	0 (0%)
Telluraves	171	169 (99%)	0 (0%)	2 (1%)	171	169 (98%)	1 (1%)	1 (1%)
Passerea	154	148 (96%)	5 (3%)	1 (1%)	153	150 (98%)	3 (2%)	0 (0%)
Neoavian	162	162 (100%)	0 (0%)	0 (0%)	163	161 (99%)	2 (0%)	0 (0%)
Neognathae	163	162 (99%)	1 (1%)	0 (0%)	162	162 (100%)	0 (0%)	0 (0%)
Avian	163	163 (100%)	0 (0%)	0 (0%)	164	163 (99%)	1 (1%)	0 (0%)

Table S7: Comparison between Jarvis et al. (2014) and Prum et al. (2015) “sister” clades ancestors.

	Jarvis adjacencies in Prum				Prum adjacencies in Jarvis			
**Ancestor name**	**No. adjacencies**	**Maintained**	**Extra**	**Inconsistent**	**No. adjacencies**	**Maintained**	**Extra**	**Inconsistent**
Jarvis (Estrildidae & Thraupidae & Fringillidae) versus Prum (Estrildidae & Thraupidae)	62	51 (82%)	8 (13%)	3 (5%)	53	49 (92%)	3 (6%)	1 (2%)
Jarvis (Passeri) versus Prum (Passeriformes)	91	78 (86%)	10 (14%)	3 (3%)	97	80 (82%)	12 (12%)	5 (5%)

Table S8: Comparison between Jarvis et al. (2014) and Reddy et al. (2017) “sister” clades ancestors.

	Jarvis adjacencies in Reddy				Reddy adjacencies in Jarvis			
**Ancestor name**	**No. adjacencies**	**Maintained**	**Extra**	**Inconsistent**	**No. adjacencies**	**Maintained**	**Extra**	**Inconsistent**
Jarvis (Passeroidea) versus Reddy (Estrildidae & Thraupidae & Fringilidae & Muscicapidae & Paridae)	81	78 (96%)	1 (1%)	2 (3%)	89	80 (90%)	7 (8%)	2 (2%)
Jarvis (Passeroidea & Paroidea) versus Reddy (Passeri)	91	91 (100%)	0 (0%)	0 (0%)	96	91 (95%)	5 (5%)	0 (0%)

Table S9: Number of EBRs and rearrangement rates for the reconstructed ancestral genomes following Jarvis et al. (2014) topology.

**Branch**			**Branch length (MY)**	**No. EBRs**	**EBRs per MY**	**FDR corrected p-value [a]**
Avian	→	Neognathae	13.1	19	1.45	0.277
Neognathae	→	Neoavian	19.4	8	0.41	0.006
Neoavian	→	Passerea	1.2	6	5.00	1.61E-04
Passerea	→	Telluraves & Aequornithia & Gruae	1.2	1	0.83	0.031
Telluraves & Aequornithia & Gruae	→	Telluraves & Aequornithia	1.3	0	0.00	0.002
Telluraves & Aequornithia	→	Telluraves	2.0	2	1.00	0.057
Telluraves	→	Eufalconimorphae	3.5	14	3.71	0.005
Eufalconimorphae	→	Psittacopasserae	5.4	6	1.11	0.081
Psittacopasserae	→	Passeriformes	22.6	47	2.08	0.882
Passeriformes	→	Passeri	8.1	29	3.58	0.006
Passeri	→	Passeroidea & Paroidea	3.2	1	0.31	0.005
Passeroidea & Paroidea	→	Passeroidea	6.0	14	2.33	0.561
Passeroidea	→	Estrildidae & Thraupidae & Fringilidae	4.6	8	1.74	0.581
Estrildidae & Thraupidae & Fringilidae	→	Zebra finch	9.9	46	4.65	2.84E-04

[a] Compared to the average across all branches (2.01 EBRs/MY).

Table S10: Number of EBRs and rearrangement rates for the reconstructed ancestral genomes following Prum et al. (2015) topology.

**Branch**			**Branch length (MY)**	**No. EBRs**	**EBRs per MY**	**FDR corrected p-value [a]**
Avian	→	Neognathae	1	12	12.00	2.02E-04
Neognathae	→	Neoavian	4	13	3.25	0.250
Neoavian	→	Telluraves & Aequornithia & Gruae & Cuculidae & Columbidae	1	10	10.00	0.003
Telluraves & Aequornithia & Gruae & Cuculidae & Columbidae	→	Telluraves & Aequornithia & Gruae	1	13	13.00	1.05E-04
Telluraves & Aequornithia & Gruae	→	Telluraves & Opisthocomidae	1	7	7.00	0.130
Telluraves & Opisthocomidae	→	Telluraves	1	1	1.00	0.008
Telluraves	→	Eufalconimorphae & Picidae	1	1	1.00	0.008
Eufalconimorphae & Picidae	→	Eufalconimorphae	3	3	1.00	0.008
Eufalconimorphae	→	Psittacopasserae	3	11	3.67	0.396
Psittacopasserae	→	Passeriformes	9	42	4.67	0.910
Passeriformes	→	Estrildidae & Thraupidae & Muscicapidae & Pipridae	20	7	0.35	0.006
Estrildidae & Thraupidae & Muscicapidae & Pipridae	→	Estrildidae & Thraupidae & Muscicapidae	17	11	0.65	0.007
Estrildidae & Thraupidae & Muscicapidae	→	Estrildidae & Thraupidae	3	15	5.00	0.910
Estrildidae & Thraupidae	→	Zebra finch	7	39	5.57	0.640

[a] Compared to the average across all branches (4.87 EBRs/MY).

Table S11: Number of EBRs and rearrangement rates for the reconstructed ancestral genomes following Reddy et al. (2017) topology.

Branch			Branch length (MY)	No. EBRs	EBRs per MY	FDR corrected p-value [a]
Avian	→	Neognathae	13	14	1.08	0.223
Neognathae	→	Neoavian	20	11	0.55	0.174
Neoavian	→	Passerea	1	25	25.00	5.25E-08
Passerea	→	Telluraves & Apodiformes & Gruae	2	18	9.00	0.056
Telluraves & Apodiformes & Gruae	→	Telluraves & Apodiformes & Opisthocomidae	1	6	6.00	0.301
Telluraves & Apodiformes & Opisthocomidae	→	Telluraves & Apodiformes	1	0	0.00	0.115
Telluraves & Apodiformes	→	Telluraves	1	1	1.00	0.223
Telluraves	→	Eufalconimorphae	4	7	1.75	0.301
Eufalconimorphae	→	Psittacopasserae	4	9	2.25	0.395
Psittacopasserae	→	Passeriformes	23	32	1.39	0.270
Passeriformes	→	Passeri	8	19	2.38	0.395
Passeri	→	Estrildidae & Thraupidae & Fringilidae & Muscicapidae & Paridae	3	5	1.67	0.301
Estrildidae & Thraupidae & Fringilidae & Muscicapidae & Paridae	→	Estrildidae & Thraupidae & Fringilidae & Muscicapidae	6	0	0.00	0.115
Estrildidae & Thraupidae & Fringilidae & Muscicapidae	→	Estrildidae & Thraupidae & Fringilidae	5	12	2.40	0.395
Estrildidae & Thraupidae & Fringilidae	→	Zebra finch	10	42	4.20	0.862

[a] Compared to the average across all branches (3.91 EBRs/MY)

Table S12: EBR distribution (observed/expected number of EBRs) for Avian ancestor chromosomes.

Avian chr.	Length (Mbp)	Obs – Exp no. EBRs
**1**	151.05	- 7 *
**2**	126.80	- 8 *
**3**	89.44	- 9 *
**4**	56.83	2
**4A**	16.17	2
**5**	51.05	0
**6**	30.27	- 3 *
**7**	32.92	0
**8**	21.98	- 3 *
**9**	22.93	- 2 *
**10**	16.99	- 3 *
**11**	17.39	4 *
**12**	17.26	0
**13**	13.84	1
**14**	11.98	2
**15**	11.73	1
**17**	9.66	- 2 *
**18**	8.79	3 *
**19**	8.90	0
**20**	10.91	1
**21**	4.15	2
**22**	2.03	0
**23**	2.97	2
**24**	4.57	2
**26**	2.91	5 *
**27**	2.10	3 *
**28**	2.63	8 *
**Z**	42.67	16 *
**Average**	-	- 1

Supplementary Figures

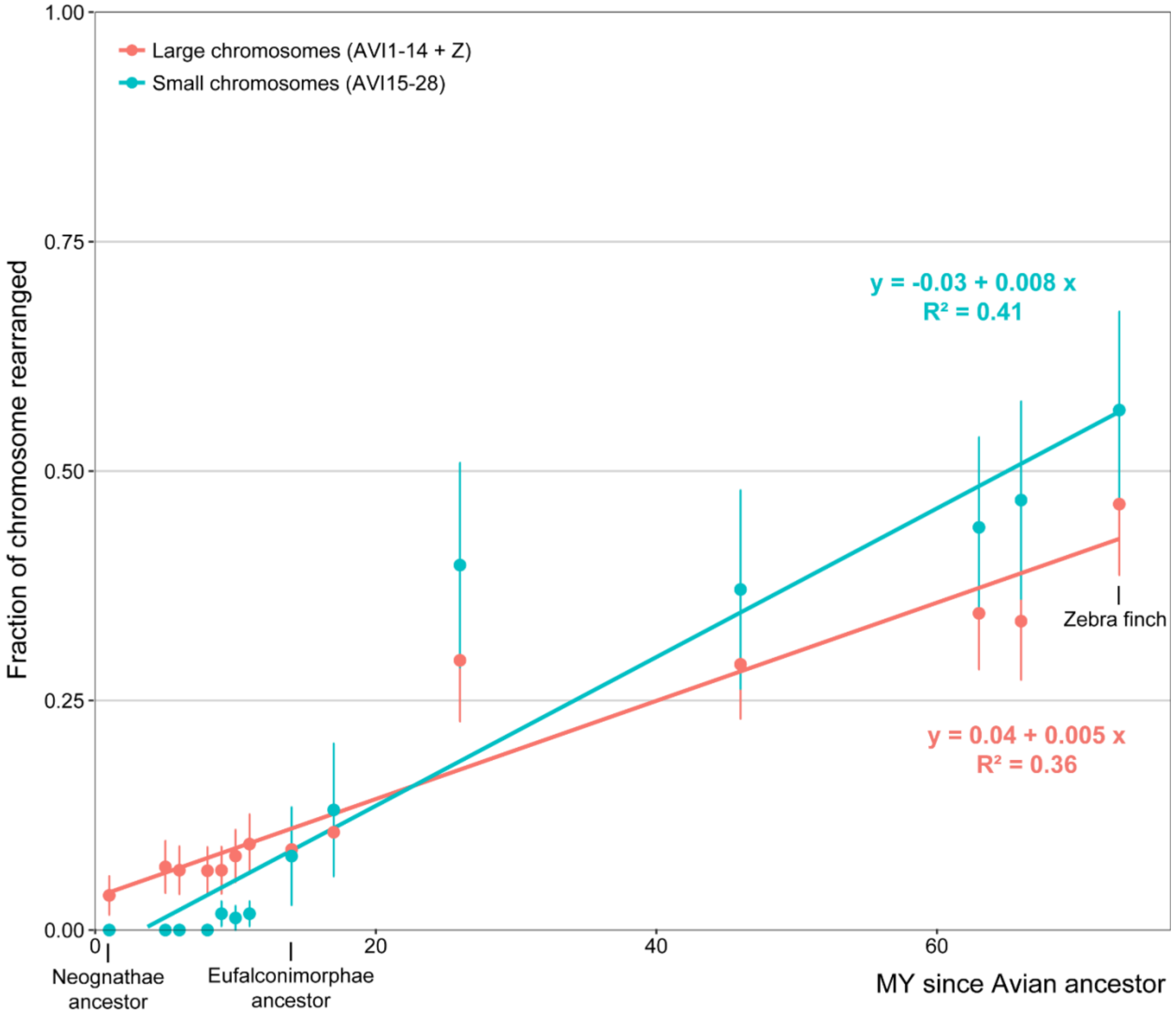

Figure S2: Association between the fraction of chromosome rearranged and evolutionary time for larger (AVI1-14 + Z) and smaller Avian ancestor chromosomes (AVI15-28) for Prum et al. (2015) topology. Blue and orange lines depict linear regressions for smaller and larger Avian ancestor chromosomes, respectively.

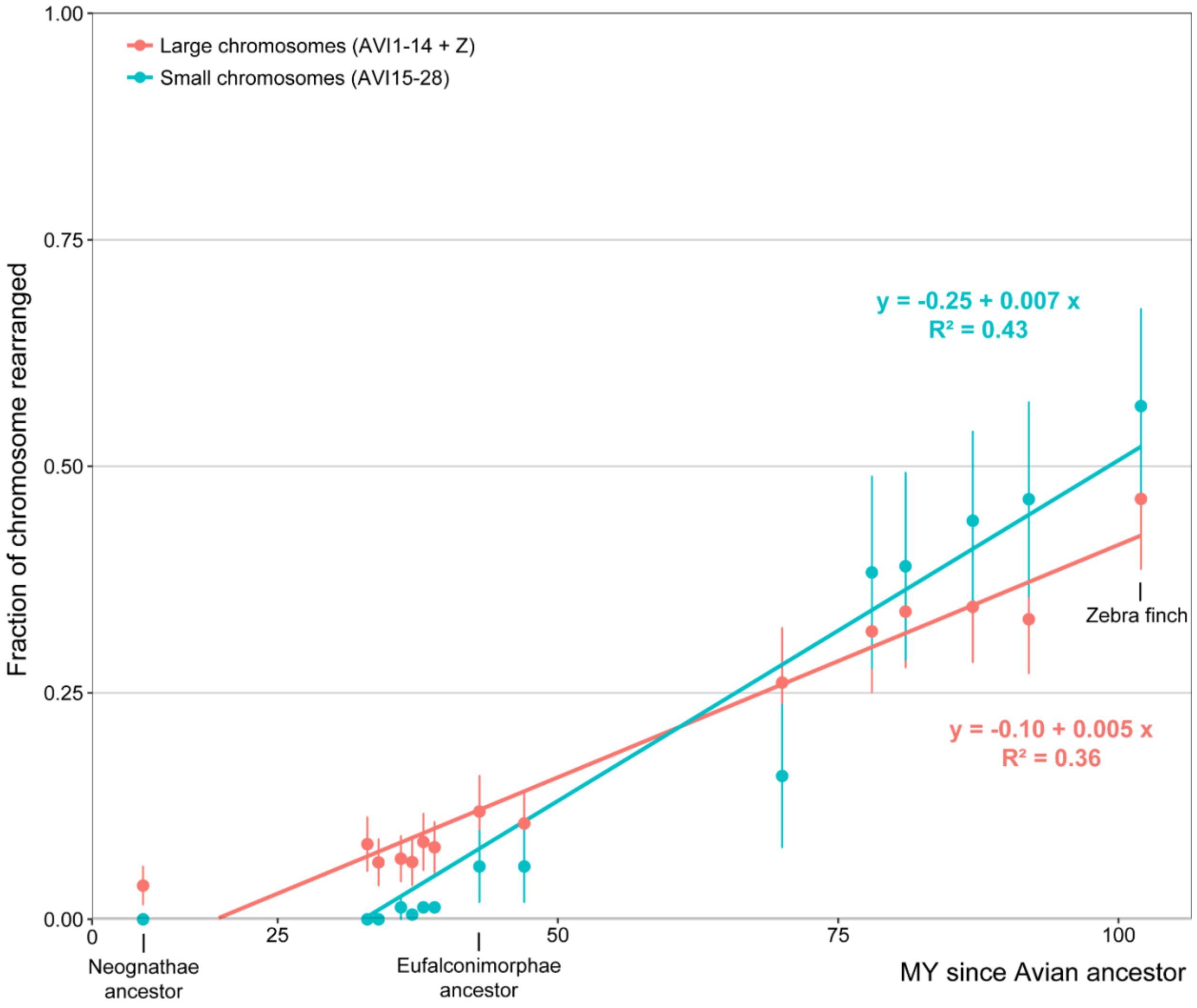

Figure S3: Association between the fraction of chromosome rearranged and evolutionary time for larger (AVI1-14 + Z) and smaller Avian ancestor chromosomes (AVI15-28) for Reddy et al. (2017) topology. Blue and orange lines depict linear regressions for smaller and larger Avian ancestor chromosomes, respectively.

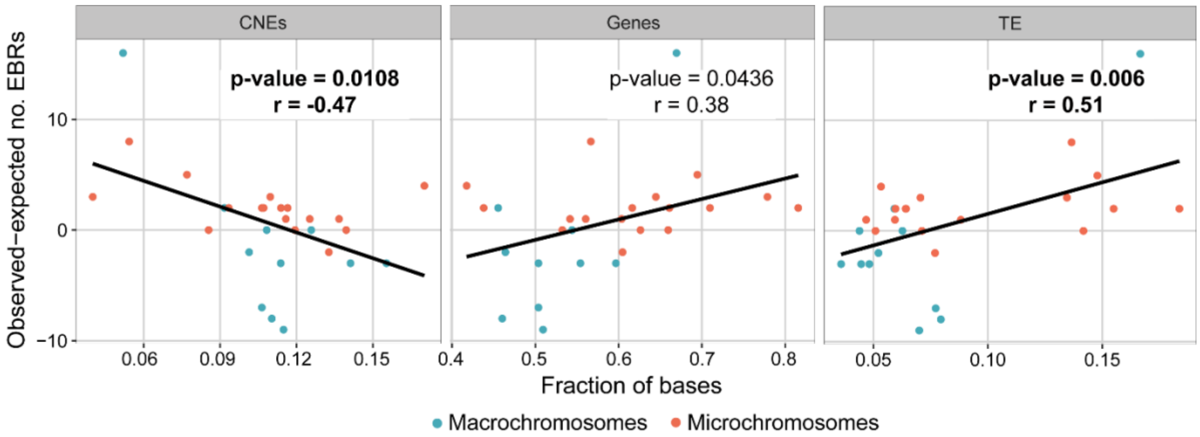

Figure S4: Correlation between the fraction of bases within CNEs, TEs, genes and measurements of EBRs distribution (observed/expected number of EBRs) for Avian ancestor chromosomes. The black line shows linear correlation, and r and p-values show the Pearson correlation. Bold depicts significant correlations. Blue and orange circles depict macro- (length ≥ 20Mbp) and microchromosomes (length < 20Mbp in the zebra finch genome), respectively.

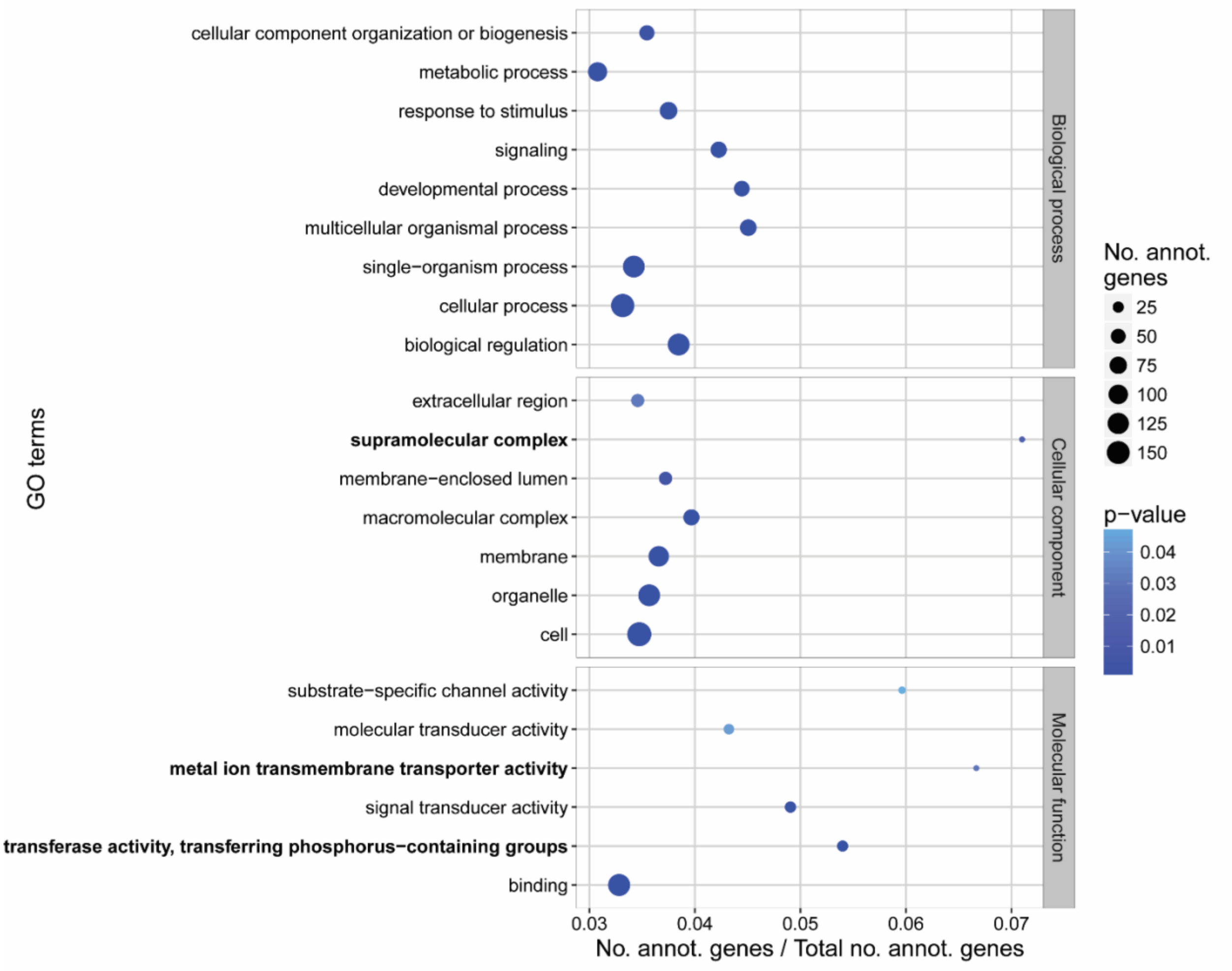

Figure S5: GO terms enriched on Avian ancestor chromosomes 26, 27 and 28 (p-value < 0.05; FDR < 5%). Bubble size depicts the number of genes annotated in each GO term. Bubble shade represents the p-value with darker shades for lower p-values. The x-axis shows the ratio of genes annotated for each GO term in the analysed list versus the background list. GO terms unique for highly rearranged chromosomes are depicted in bold.
