# Supplementary material for: Reconstruction of avian ancestral karyotypes reveals differences in the evolutionary history of macro- and microchromosomes
Source: Genome Biol. 2018 Oct 5;19:155. doi: 10.1186/s13059-018-1544-8 (PMC6173868; doi:10.1186/s13059-018-1544-8)
Supplement: Supplementary file 2 — Evolution highway visualisations of Avian ancestor chromosomes. (PDF 2360 kb) [file 13059_2018_1544_MOESM2_ESM.pdf]

Figure S1: Avian ancestor chromosome visualizations on the Evolution Highway comparative chromosome browser. Blue and pink blocks define syntenic fragments, in “+” and “-” orientation against the Avian ancestor chromosomes, respectively. Number within blocks depict chromosome numbers in each of the reconstructed ancestors, and chromosome or scaffold number in each extant descendant and outgroup species.

AVI1

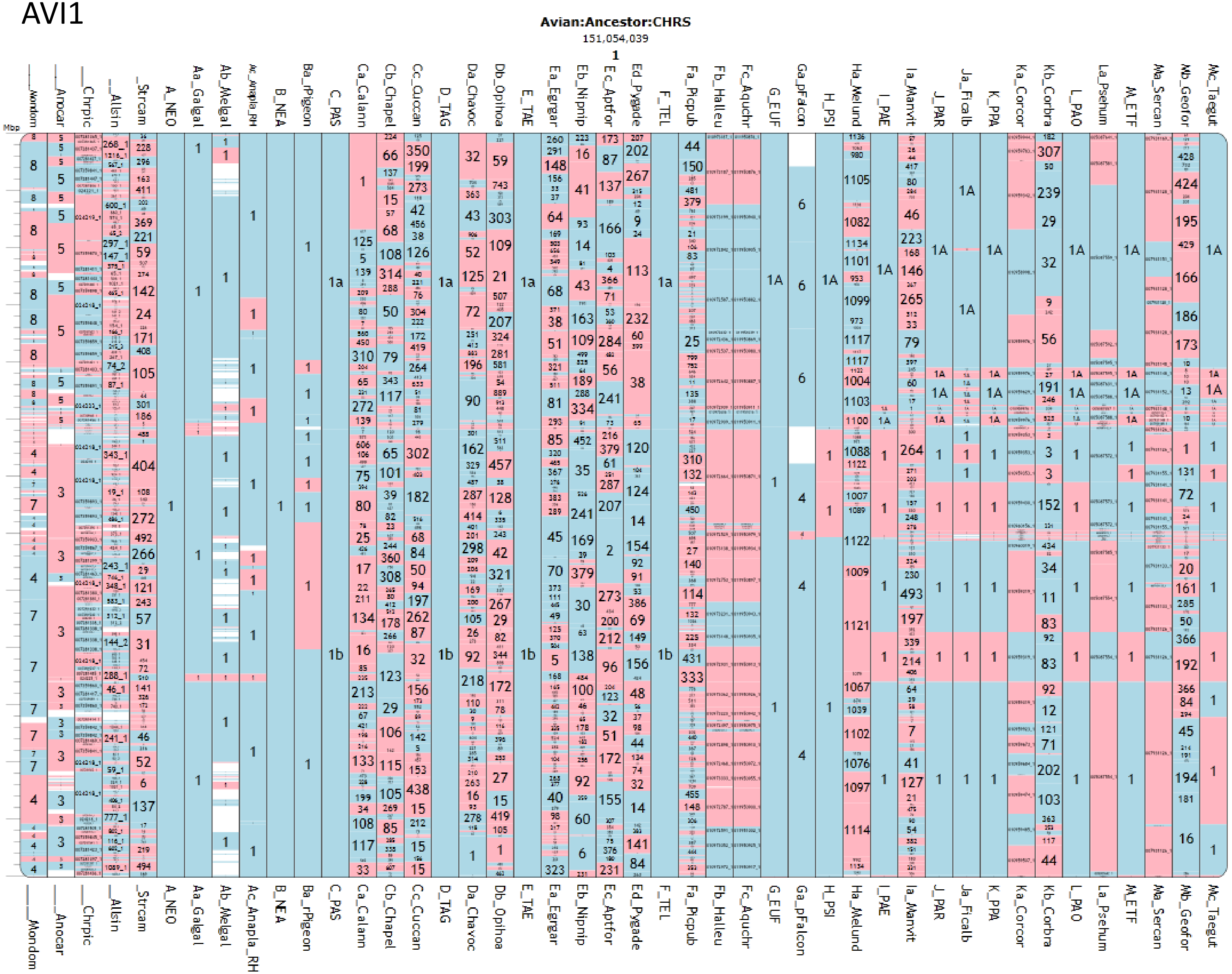
Avian:Ancestor:CHRS
151,054,039
1
Mbp
__Mondom
__Anocar
__Chrpic
__Allsin
_Strcam
A_NEO
Aa_Galgal
Ab_Melgal
Ac_Anapla_RH
B_NEA
Ba_rPigeon
C_PAS
Ca_Calann
Cb_Chapel
Cc_Cuccan
D_TAG
Da_Chavoc
Db_Opihoa
E_TAE
Ea_Egrgar
Eb_Nipnip
Ec_Aptfor
Ed_Pygade
F_TEL
Fa_Picpub
Fb_Halleu
Fc_Aquchr
G_EUF
Ga_pFalcon
H_PSI
Ha_Melund
I_PAE
Ia_Manvit
J_PAR
Ja_Ficalb
K_PPA
Ka_Corcor
Kb_Corbra
L_PAO
La_Psehum
M_ETF
Ma_Sercan
Mb_Geofor
Mc_Taegut

AVI2

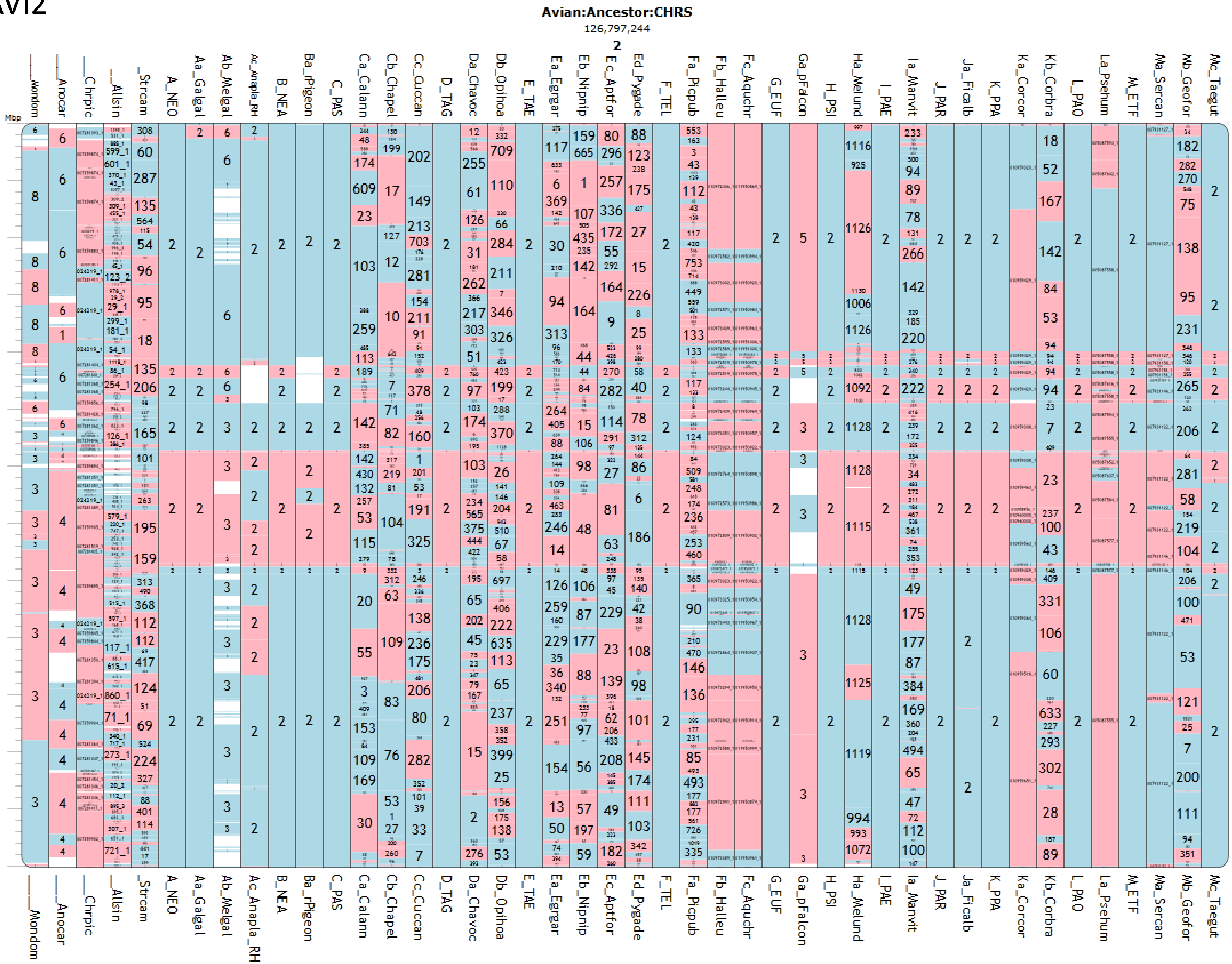

Avian:Ancestor:CHRS
126,797,244
2
Mbp
___Mondom
___Anocar
___Chrpic
__Allsin
_Strcam
A_NEO
Aa_Galgal
Ab_Melgal
Ac_Anapla_RH
B_NEA
Ba_rPigeon
C_PAS
Ca_Calann
Cb_Chapel
Cc_Cuccan
D_TAG
Da_Chavoc
Db_Opihoa
E_TAE
Ea_Egrgar
Eb_Nipnip
Ec_Aptfor
Ed_Pygade
F_TEL
Fa_Picpub
Fb_Halleu
Fc_Aquchr
G_EUF
Ga_pFalcon
H_PSI
Ha_Melund
I_PAE
Ia_Manvit
J_PAR
Ja_Ficalb
K_PPA
Ka_Corcor
Kb_Corbra
L_PAO
La_Psehum
M_ETF
Ma_Sercan
Mb_Geofor
Mc_Taegut

AVI3

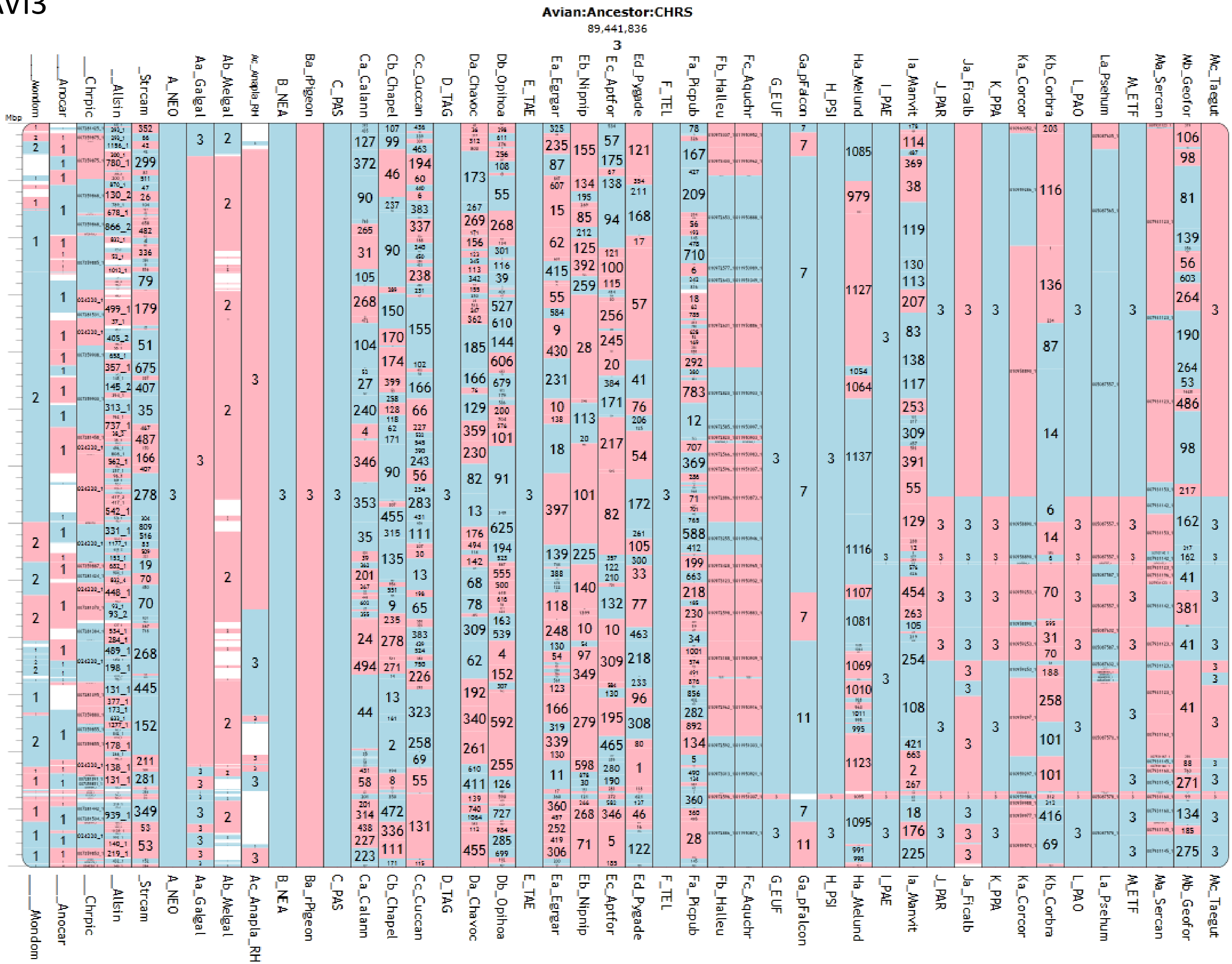
Avian:Ancestor:CHRS
89,441,836
3
Mbp
___Mondom
___Anocar
___Chrpic
__Allsin
_Strcam
A_NEO
Aa_Galgal
Ab_Melgal
Ac_Anapla_RH
B_NEA
Ba_Pigeon
C_PAS
Ca_Calann
Cb_Chapel
Cc_Cuccan
D_TAG
Da_Chavoc
Db_Opihoa
E_TAE
Ea_Egrgar
Eb_Nipnip
Ec_Aptfor
Ed_Pygade
F_TEL
Fa_Picpub
Fb_Halleu
Fc_Aquchr
G_EUF
Ga_pFalcon
H_PSI
Ha_Melund
I_PAE
Ia_Manvit
J_PAR
Ja_Ficalb
K_PPA
Ka_Corcor
Kb_Corbra
L_PAO
La_Psehum
M_ETF
Ma_Sercan
Mb_Geofor
Mc_Taegut

AVI4

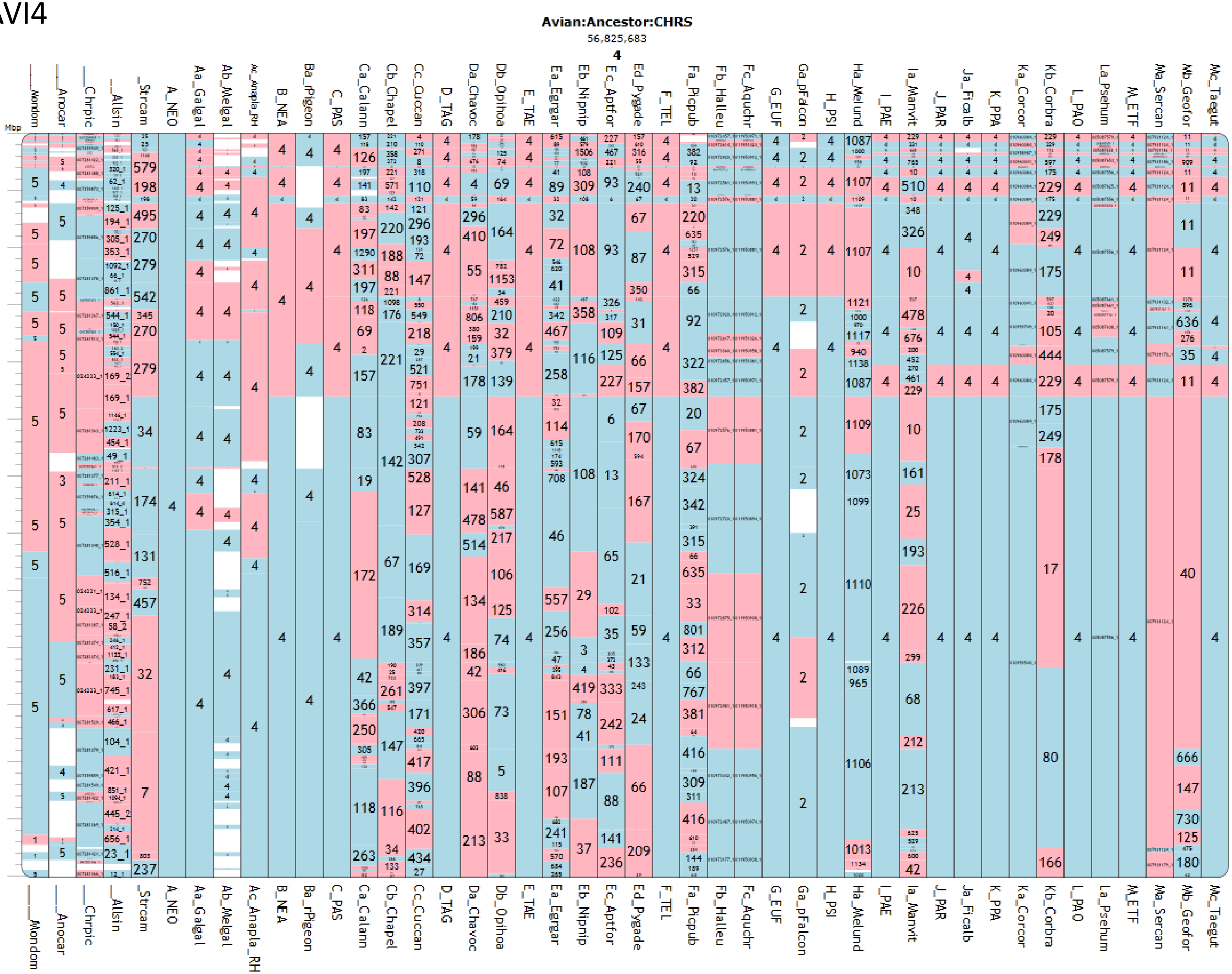
Avian:Ancestor:CHRS
56,825,683
4
Mbp
___Mondom
__Anocar
__Chrpic
__Allsin
_Strcam
A_NEO
Aa_Galgal
Ab_Melgal
Ac_Anapla_RH
B_NEA
Ba_rPigeon
C_PAS
Ca_Calann
Cb_Chapel
Cc_Cuccan
D_TAG
Da_Chavoc
Db_Opihoa
E_TAE
Ea_Egrgar
Eb_Nipnip
Ec_Aptfor
Ed_Pygade
F_TEL
Fa_Picpub
Fb_Halleu
Fc_Aquchr
G_EUF
Ga_pFalcon
H_PSI
Ha_Melund
I_PAE
Ia_Manvit
J_PAR
Ja_Ficalb
K_PPA
Ka_Corcor
Kb_Corbra
L_PAO
La_Psehum
M_ETF
Ma_Sercan
Mb_Geofor
Mc_Taegut

AVI4A

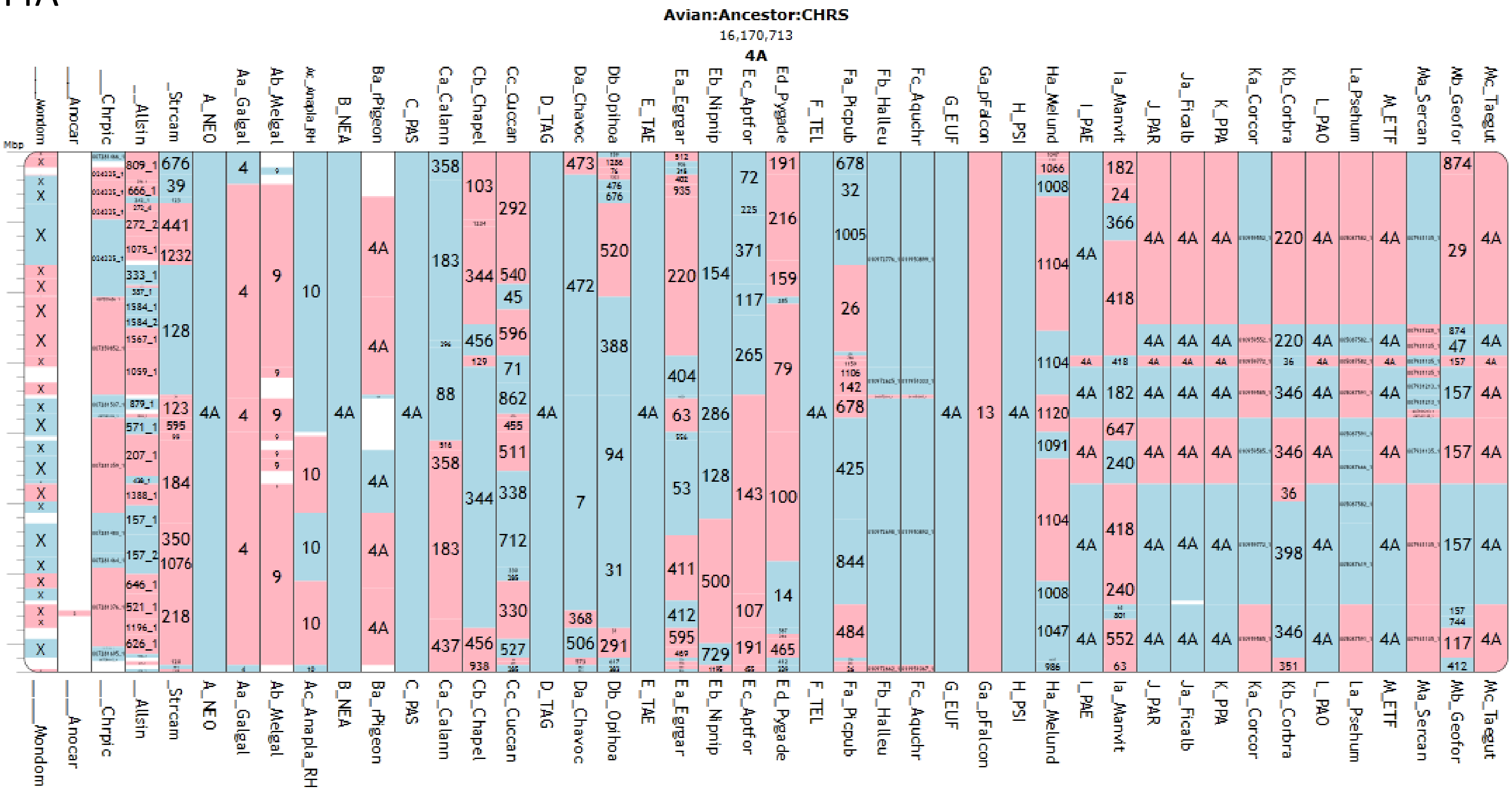
Avian:Ancestor:CHRS
16,170,713
4A
Mbp
___Mondom
___Anocar
__Chrpic
__Allsin
_Strcam
A_NEO
Aa_Galgal
Ab_Melgal
Ac_Anapla_RH
B_NEA
Ba_rPigeon
C_PAS
Ca_Calann
Cb_Chapel
Cc_Cuccan
D_TAG
Da_Chavoc
Db_Opihoa
E_TAE
Ea_Egrgar
Eb_Nipnip
Ec_Aptfor
Ed_Pygade
F_TEL
Fa_Picpub
Fb_Halleu
Fc_Aquchr
G_EUF
Ga_pFalcon
H_PSI
Ha_Melund
I_PAE
Ia_Manvit
J_PAR
Ja_Ficalb
K_PPA
Ka_Corcor
Kb_Corbra
L_PAO
La_Psehum
M_ETF
Ma_Sercan
Mb_Geofor
Mc_Taegut

AVI5

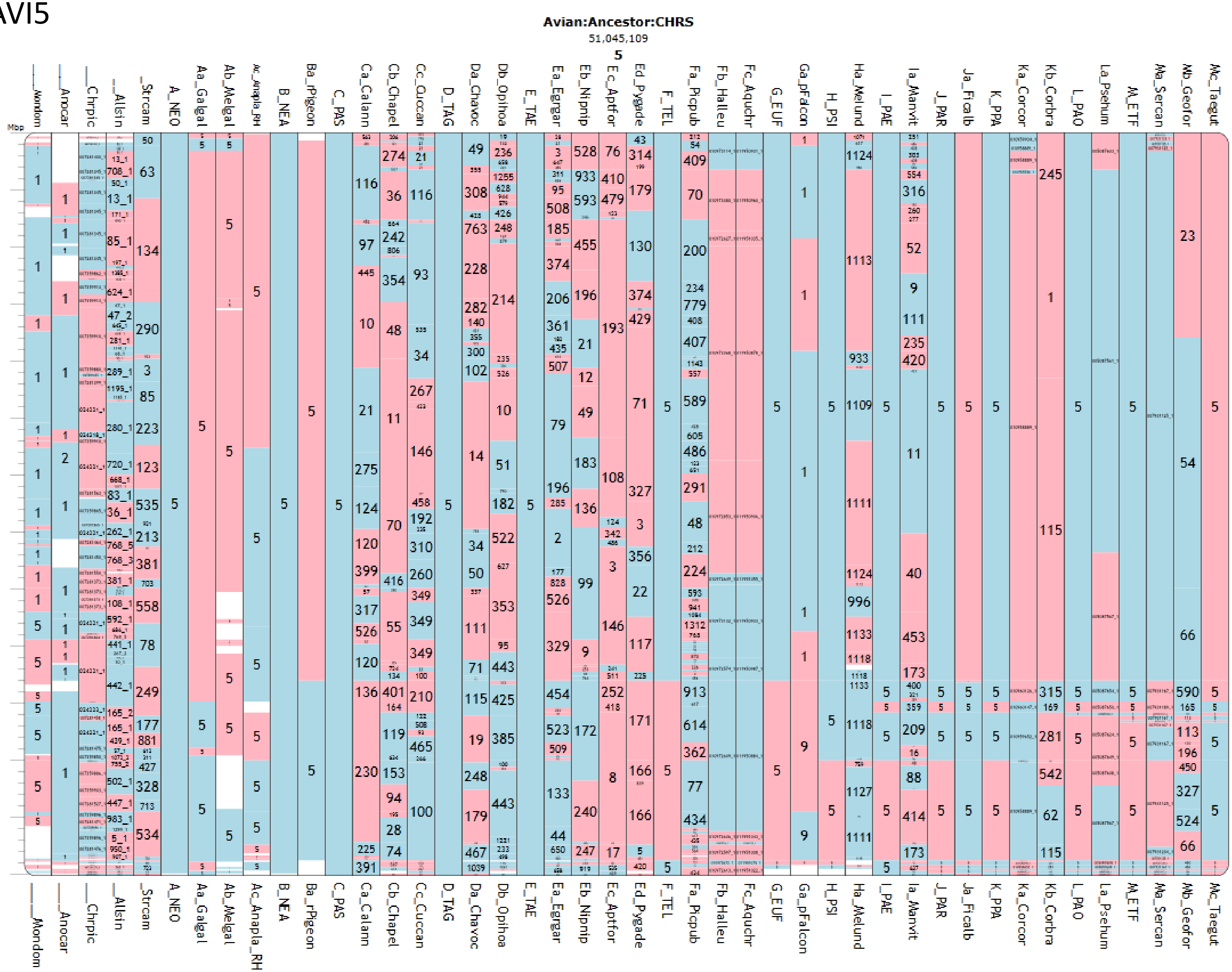
Avian:Ancestor:CHRS
51,045,109
5
Mbp
___Mondom
___Anocar
___Chrpic
__Allsin
_Strcam
A_NEO
Aa_Galgal
Ab_Melgal
Ac_Anapla_RH
B_NEA
Ba_rPigeon
C_PAS
Ca_Calann
Cb_Chapel
Cc_Cuccan
D_TAG
Da_Chavoc
Db_Opihoa
E_TAE
Ea_Egrgar
Eb_Nipnip
Ec_Aptfor
Ed_Pygade
F_TEL
Fa_Picpub
Fb_Halleu
Fc_Aquchr
G_EUF
Ga_pFalcon
H_PSI
Ha_Melund
I_PAE
Ia_Manvit
J_PAR
Ja_Ficalb
K_PPA
Ka_Corcor
Kb_Corbra
L_PAO
La_Psehum
M_ETF
Ma_Sercan
Mb_Geofor
Mc_Taegut

AVI6

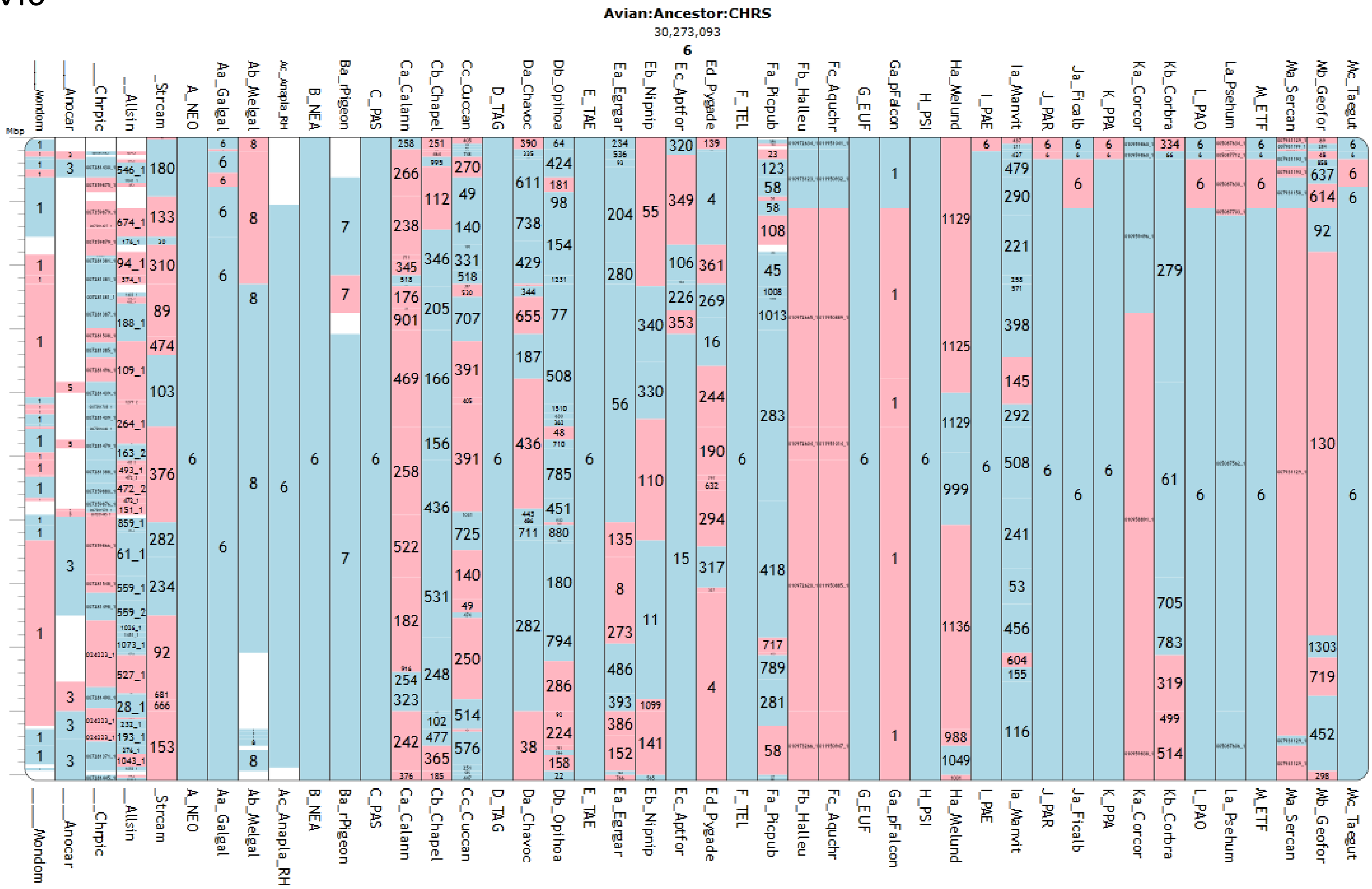

Avian:Ancestor:CHRS
30,273,093
6
Mbp
___Mondom
___Anocar
__Chrpic
__Allsin
_Strcam
A_NEO
Aa_Galgal
Ab_Melgal
Ac_Anapla_RH
B_NEA
Ba_rPigeon
C_PAS
Ca_Calann
Cb_Chapel
Cc_Cuccan
D_TAG
Da_Chavoc
Db_Opihoa
E_TAE
Ea_Egrgar
Eb_Nipnip
Ec_Aptfor
Ed_Pygade
F_TEL
Fa_Picpub
Fb_Halleu
Fc_Aquchr
G_EUF
Ga_pFalcon
H_PSI
Ha_Melund
I_PAE
Ia_Manvit
J_PAR
Ja_Ficalb
K_PPA
Ka_Corcor
Kb_Corbra
L_PAO
La_Psehum
M_ETF
Ma_Sercan
Mb_Geofor
Mc_Taegut

AVI7

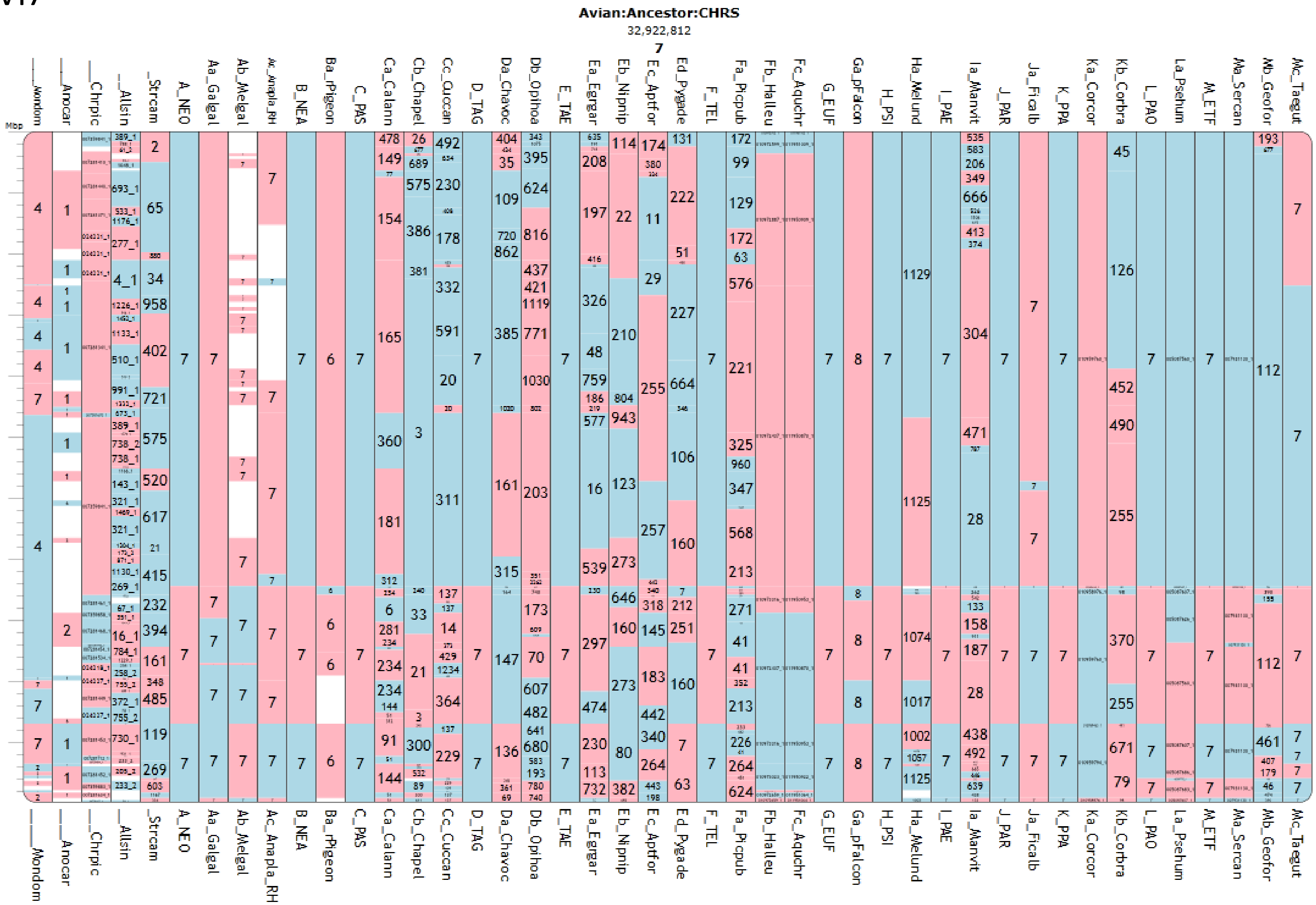

Avian:Ancestor:CHRS
32,922,812
7
Mbp
___Mondom
___Anocar
__Chrpic
__Allsin
_Strcam
A_NEO
Aa_Galgal
Ab_Melgal
Ac_Anapla_RH
B_NEA
Ba_rPigeon
C_PAS
Ca_Calann
Cb_Chapel
Cc_Cuccan
D_TAG
Da_Chavoc
Db_Ophoa
E_TAE
Ea_Egrgar
Eb_Nipnip
Ec_Aptfor
Ed_Pygade
F_TEL
Fa_Picpub
Fb_Halleu
Fc_Aquchr
G_EUF
Ga_pFalcon
H_PSI
Ha_Melund
I_PAE
Ia_Manvit
J_PAR
Ja_Ficalb
K_PPA
Ka_Corcor
Kb_Corbra
L_PAO
La_Psehum
M_ETF
Ma_Sercan
Mb_Geofor
Mc_Taegut

AVI8

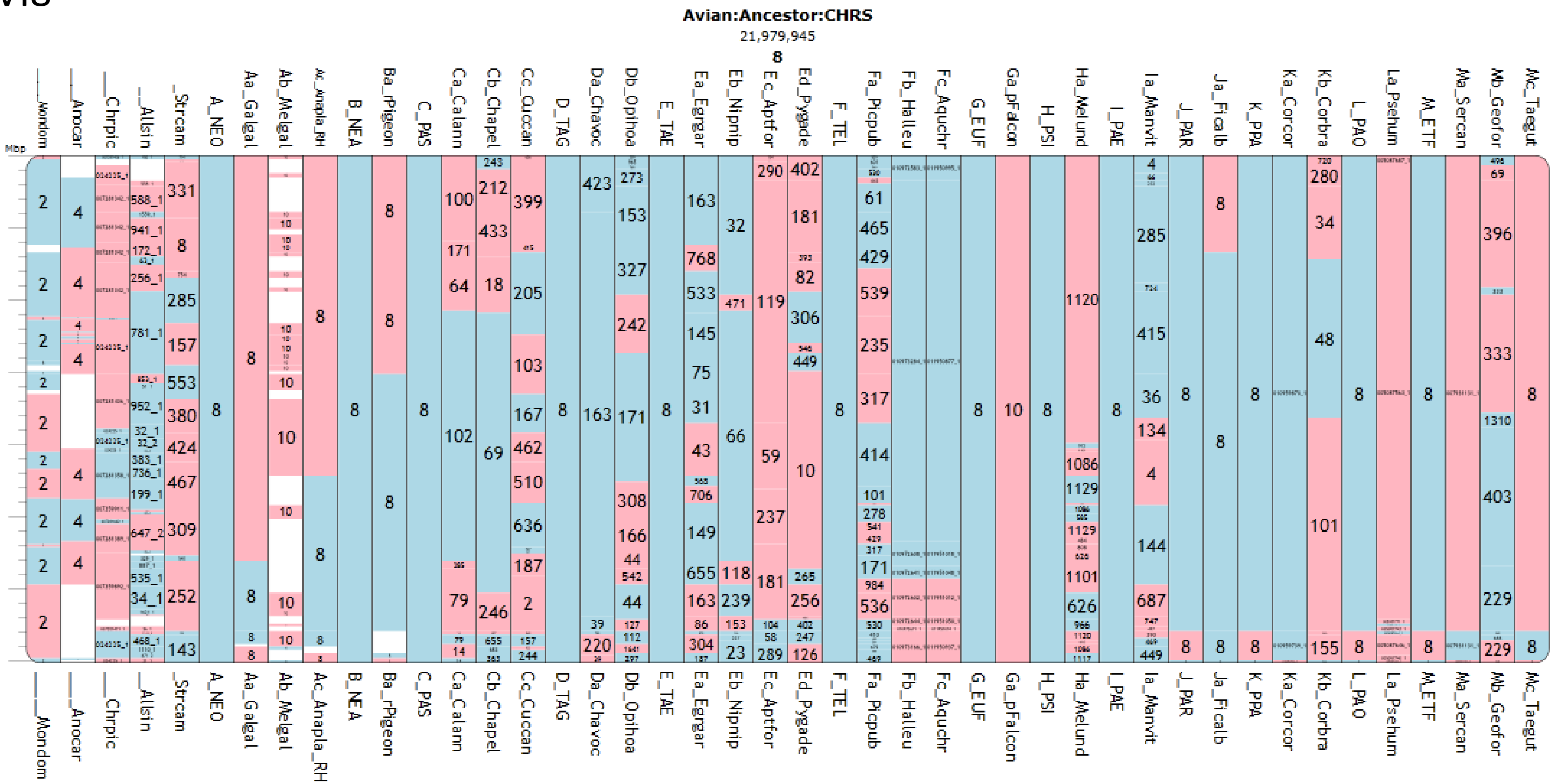
Avian:Ancestor:CHRS
21,979,945
8
Mbp
___Mondom
___Anocar
___Chrpic
__Allsin
_Strcam
A_NEO
Aa_Galgal
Ab_Melgal
Ac_Anapla_RH
B_NEA
Ba_rPigeon
C_PAS
Ca_Calann
Cb_Chapel
Cc_Cuccan
D_TAG
Da_Chavoc
Db_Opihoa
E_TAE
Ea_Egrgar
Eb_Nipnip
Ec_Aptfor
Ed_Pygade
F_TEL
Fa_Picpub
Fb_Halleu
Fc_Aquchr
G_EUF
Ga_pFalcon
H_PSI
Ha_Melund
I_PAE
Ia_Manvit
J_PAR
Ja_Ficalb
K_PPA
Ka_Corcor
Kb_Corbra
L_PAO
La_Psehum
M_ETF
Ma_Sercan
Mb_Geofor
Mc_Taegut

AVI9

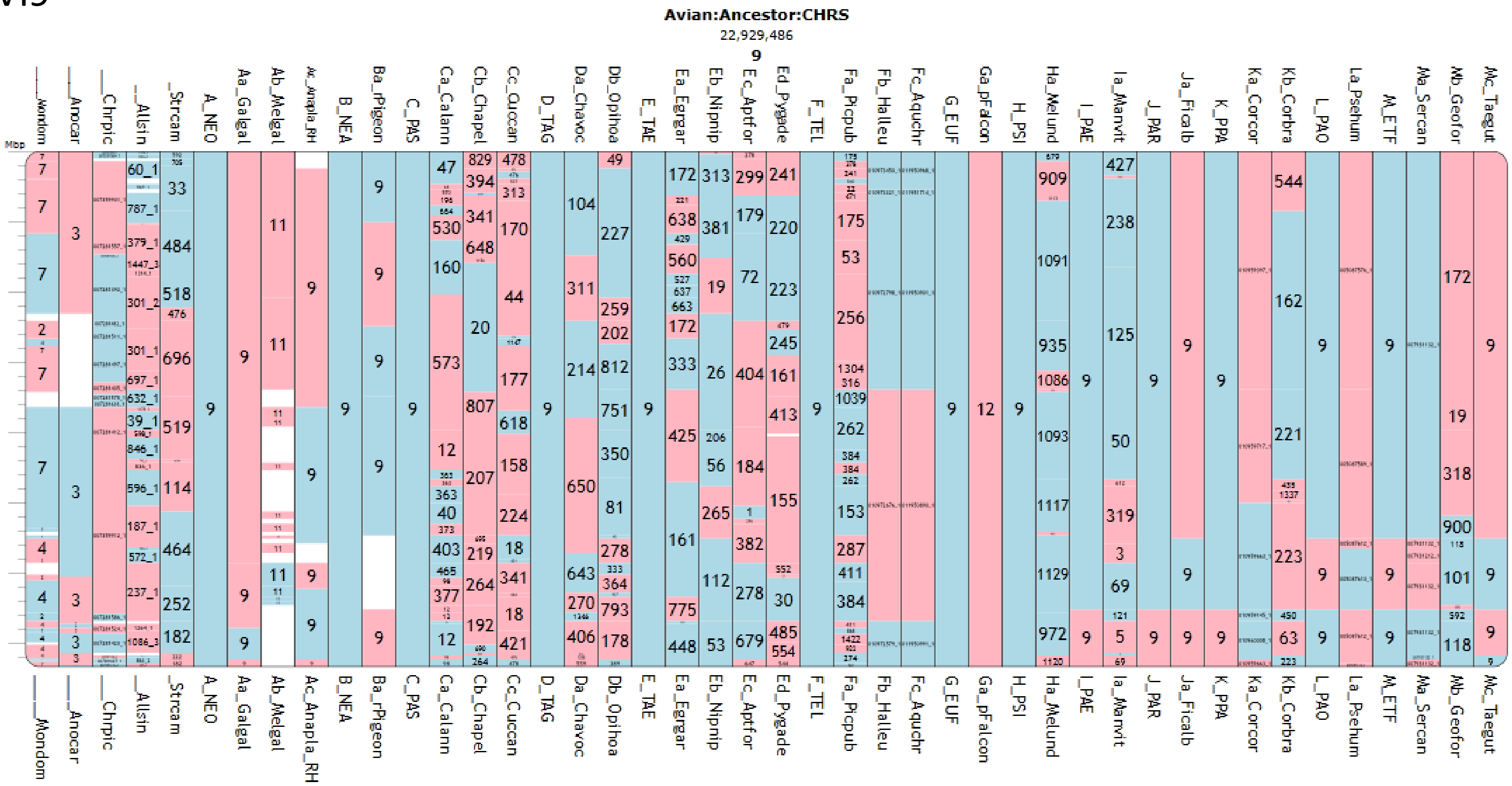

Avian:Ancestor:CHRS
22,929,486
9
Mbp
___Mondom
___Anocar
___Chrpic
__Allsin
_Strcam
A_NEO
Aa_Galgal
Ab_Melgal
Ac_Anapla_RH
B_NEA
Ba_rPigeon
C_PAS
Ca_Calann
Cb_Chapel
Cc_Cuccan
D_TAG
Da_Chavoc
Db_Opihoa
E_TAE
Ea_Egrgar
Eb_Nipnip
Ec_Aptfor
Ed_Pygade
F_TEL
Fa_Picpub
Fb_Halleu
Fc_Aquchr
G_EUF
Ga_pFalcon
H_PSI
Ha_Melund
I_PAE
Ia_Manvit
J_PAR
Ja_Ficalb
K_PPA
Ka_Corcor
Kb_Corbra
L_PAO
La_Psehum
M_ETF
Ma_Sercan
Mb_Geofor
Mc_Taegut

AVI10

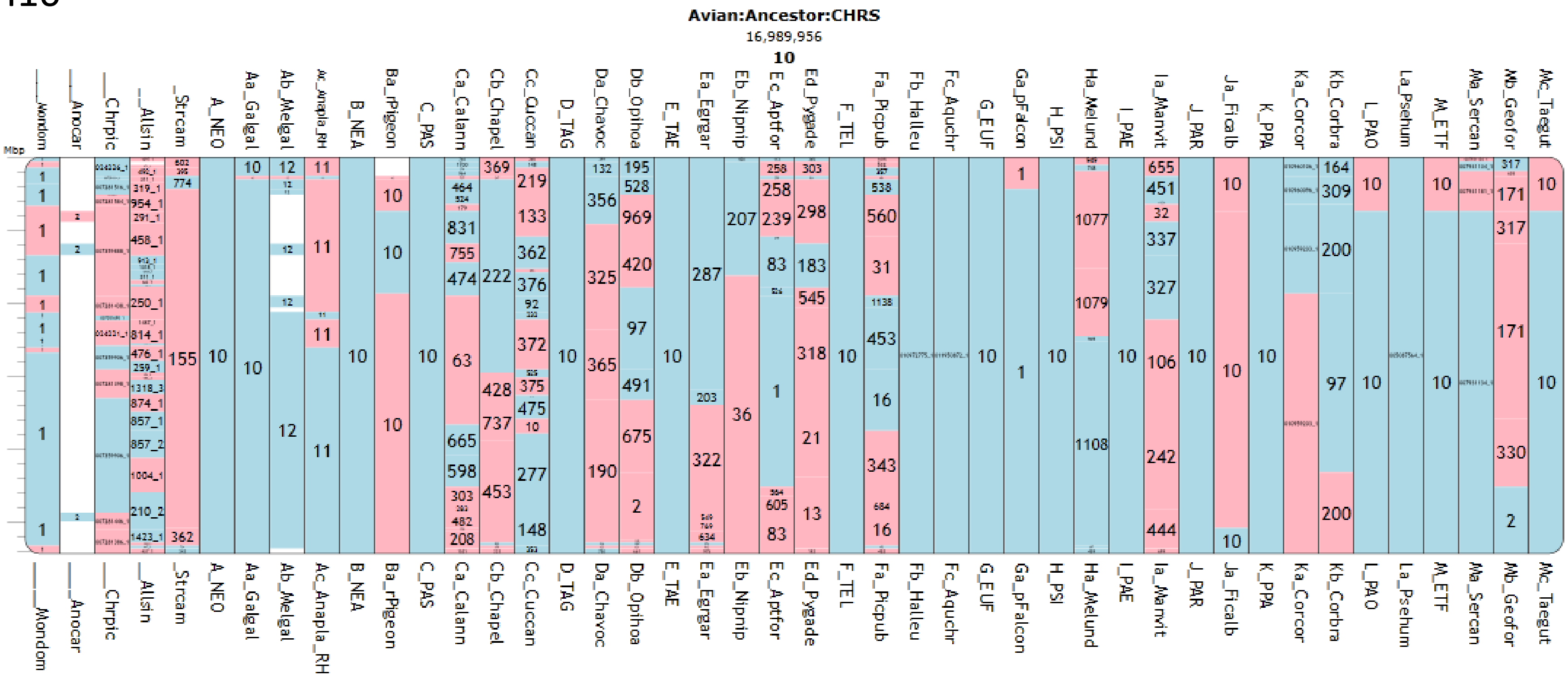
Avian:Ancestor:CHRS
16,989,956
10
Mbp
__Mondom
__Anocar
__Chrpic
__Allsin
_Strcam
A_NEO
Aa_Galgal
Ab_Melgal
Ac_Anapla_RH
B_NEA
Ba_rPigeon
C_PAS
Ca_Calann
Cb_Chapel
Cc_Cuccan
D_TAG
Da_Chavoc
Db_Opihoa
E_TAE
Ea_Egrgar
Eb_Nipnip
Ec_Aptfor
Ed_Pygade
F_TEL
Fa_Picpub
Fb_Halleu
Fc_Aquchr
G_EUF
Ga_pFalcon
H_PSI
Ha_Melund
I_PAE
Ia_Manvit
J_PAR
Ja_Ficalb
K_PPA
Ka_Corcor
Kb_Corbra
L_PAO
La_Psehum
M_ETF
Ma_Sercan
Mb_Geofor
Mc_Taegut

AVI11

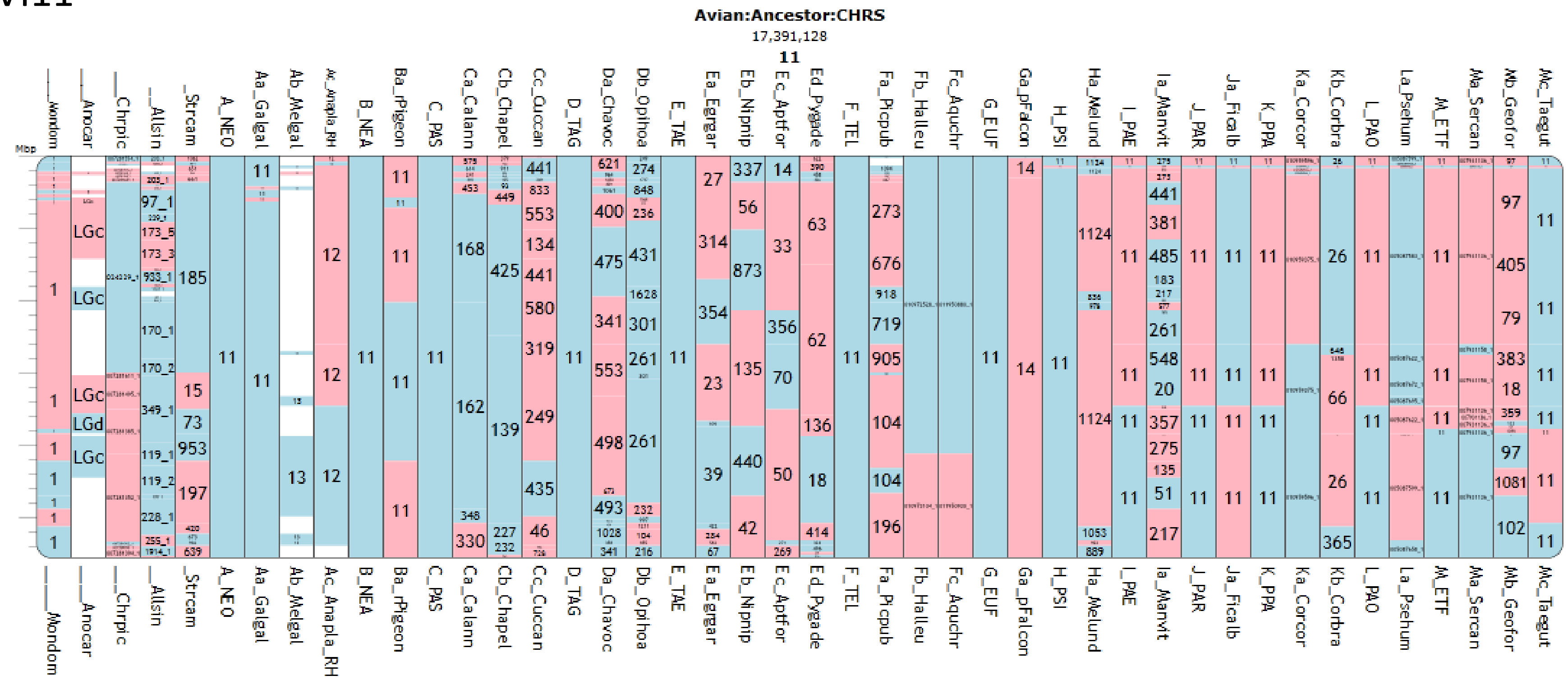
Avian:Ancestor:CHRS
17,391,128
11
Mbp
___Mondom
___Anocar
__Chrpic
__Allsin
_Strcam
A_NEO
Aa_Galgal
Ab_Melgal
Ac_Anapla_RH
B_NEA
Ba_rPigeon
C_PAS
Ca_Calann
Cb_Chapel
Cc_Cuccan
D_TAG
Da_Chavoc
Db_Opihoa
E_TAE
Ea_Egrgar
Eb_Nipnip
Ec_Aptfor
Ed_Pygade
F_TEL
Fa_Picpub
Fb_Halleu
Fc_Aquchr
G_EUF
Ga_pFalcon
H_PSI
Ha_Melund
I_PAE
Ia_Manvit
J_PAR
Ja_Ficalb
K_PPA
Ka_Corcor
Kb_Corbra
L_PAO
La_Psehum
M_ETF
Ma_Sercan
Mb_Geofor
Mc_Taegut

AVI12

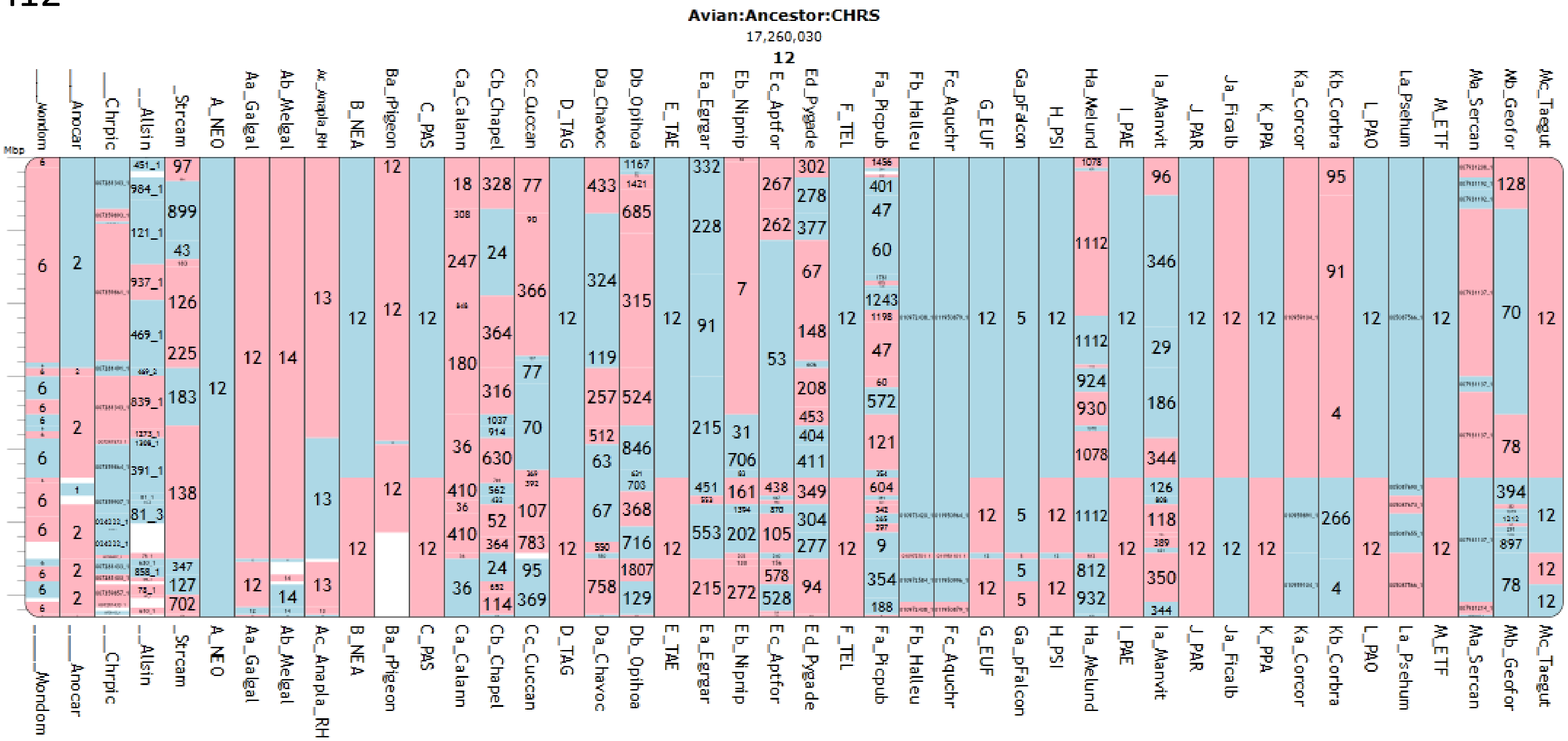

AVI13

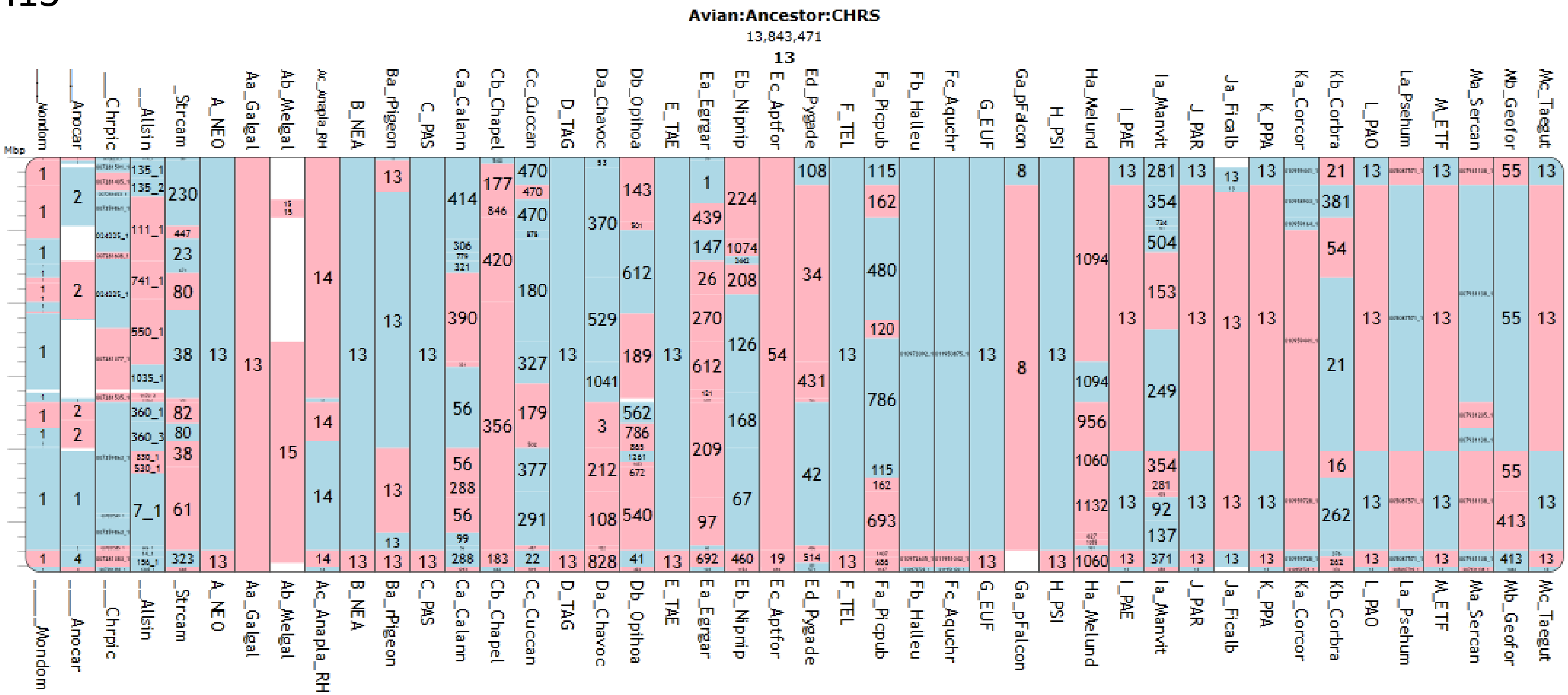

AVI14

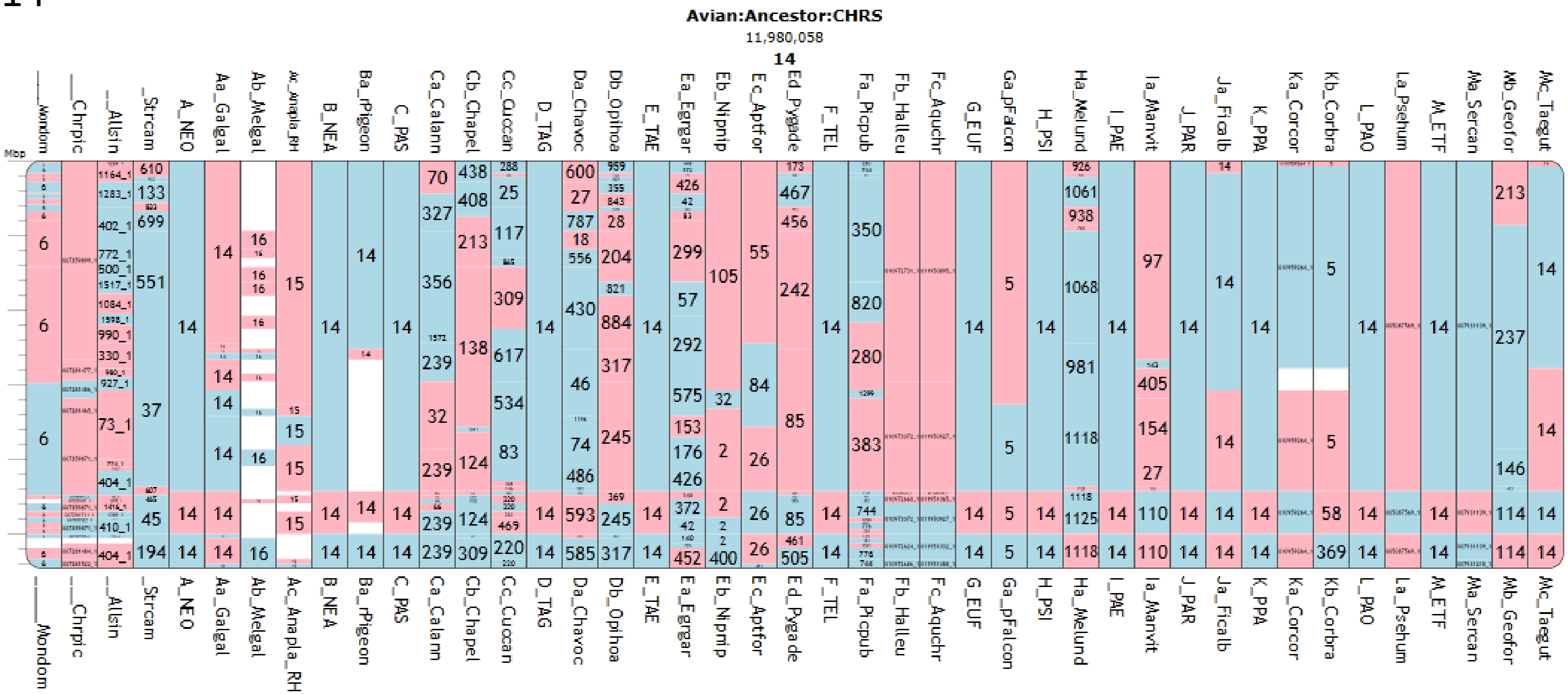

Avian:Ancestor:CHRS
11,980,058
14
Mbp
__Mondom
__Chrpic
__Allsin
_Strcam
A_NEO
Aa_Galgal
Ab_Melgal
Ac_Anapla_RH
B_NEA
Ba_Pigeon
C_PAS
Ca_Calann
Cb_Chapel
Cc_Cuccan
D_TAG
Da_Chavoc
Db_Opihoa
E_TAE
Ea_Egrgar
Eb_Nipnip
Ec_Aptfor
Ed_Pygade
F_TEL
Fa_Picpub
Fb_Halleu
Fc_Aquchr
G_EUF
Ga_pFalcon
H_PSI
Ha_Melund
I_PAE
Ia_Manvit
J_PAR
Ja_Ficalb
K_PPA
Ka_Corcor
Kb_Corbra
L_PAO
La_Psehum
M_ETF
Ma_Sercan
Mb_Geofor
Mc_Taegut

AVI15

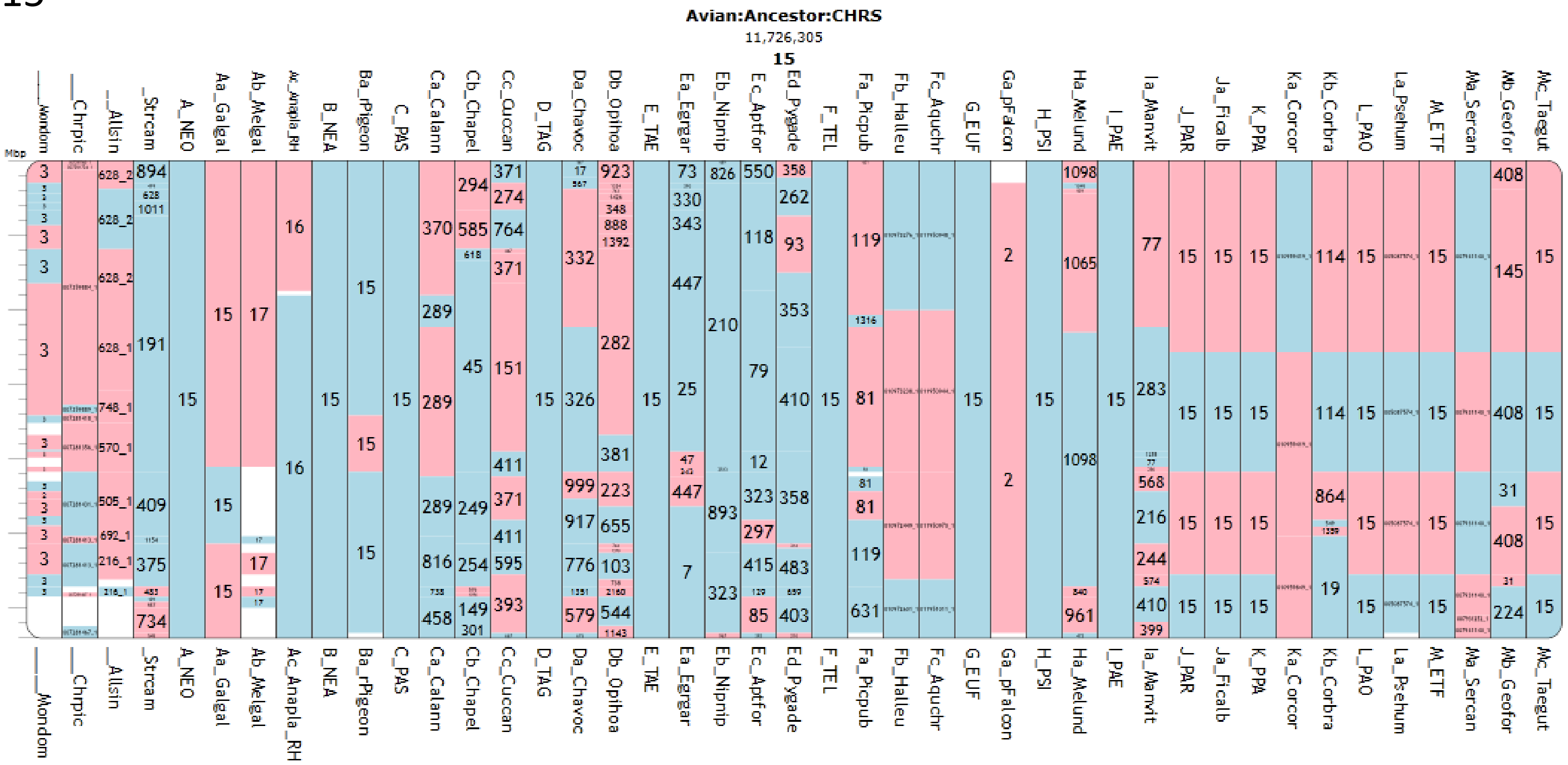

Avian:Ancestor:CHRS
11,726,305
15
Mbp
___Mondom
__Chrpic
__Allsin
_Strcam
A_NEO
Aa_Galgal
Ab_Melgal
Ac_Anapla_RH
B_NEA
Ba_Pigeon
C_PAS
Ca_Calann
Cb_Chapel
Cc_Cuccan
D_TAG
Da_Chavoc
Db_Opthoa
E_TAE
Ea_Egrgar
Eb_Nipnip
Ec_Aptfor
Ed_Pygade
F_TEL
Fa_Picpub
Fb_Halleu
Fc_Aquchr
G_EUF
Ga_pFalcon
H_PSI
Ha_Melund
I_PAE
Ia_Manvit
J_PAR
Ja_Ficalb
K_PPA
Ka_Corcor
Kb_Corbra
L_PAO
La_Psehum
M_ETF
Ma_Sercan
Mb_Geofor
Mc_Taegut

AVI17

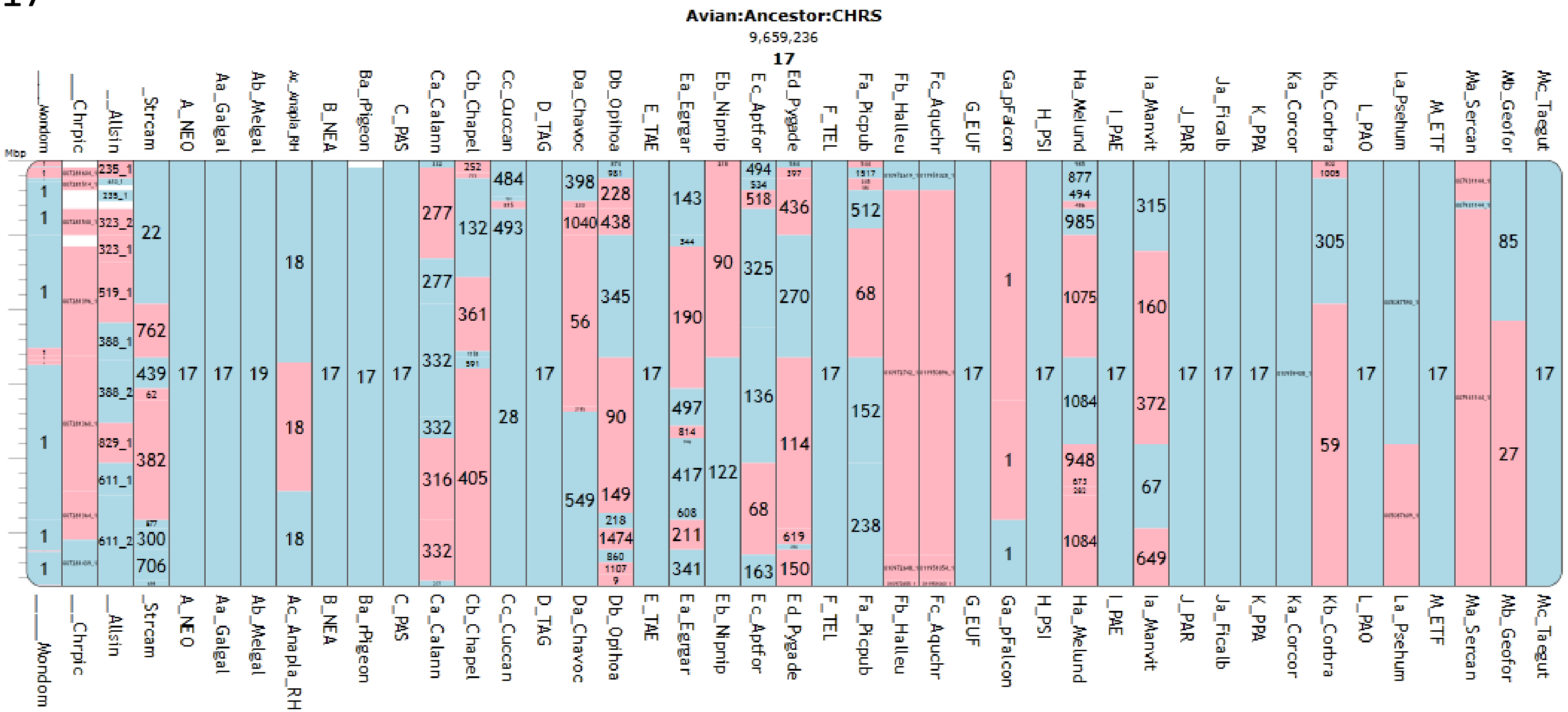

AVI18

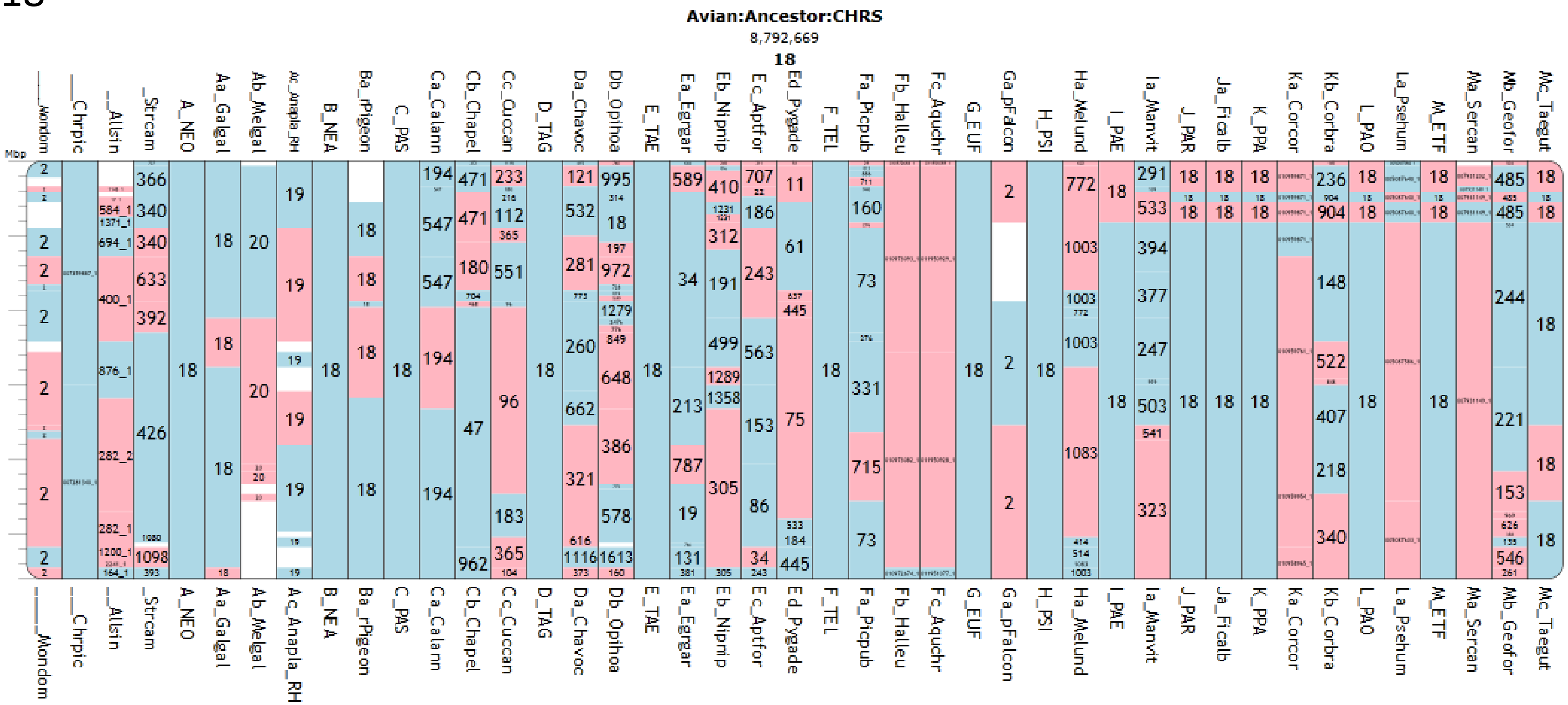

Avian:Ancestor:CHRS
8,792,669
18
Mbp
___Mondom
___Chrpic
__Allsin
_Strcam
A_NEO
Aa_Galgal
Ab_Melgal
Ac_Anapla_RH
B_NEA
Ba_Pigeon
C_PAS
Ca_Calann
Cb_Chapel
Cc_Cuccan
D_TAG
Da_Chavoc
Db_Opihoa
E_TAE
Ea_Egrgar
Eb_Nipnip
Ec_Aptfor
Ed_Pygade
F_TEL
Fa_Picpub
Fb_Halleu
Fc_Aquchr
G_EUF
Ga_pFalcon
H_PSI
Ha_Melund
I_PAE
Ia_Manvit
J_PAR
Ja_Ficalb
K_PPA
Ka_Corcor
Kb_Corbra
L_PAO
La_Psehum
M_ETF
Ma_Sercan
Mb_Geofor
Mc_Taegut

AVI19

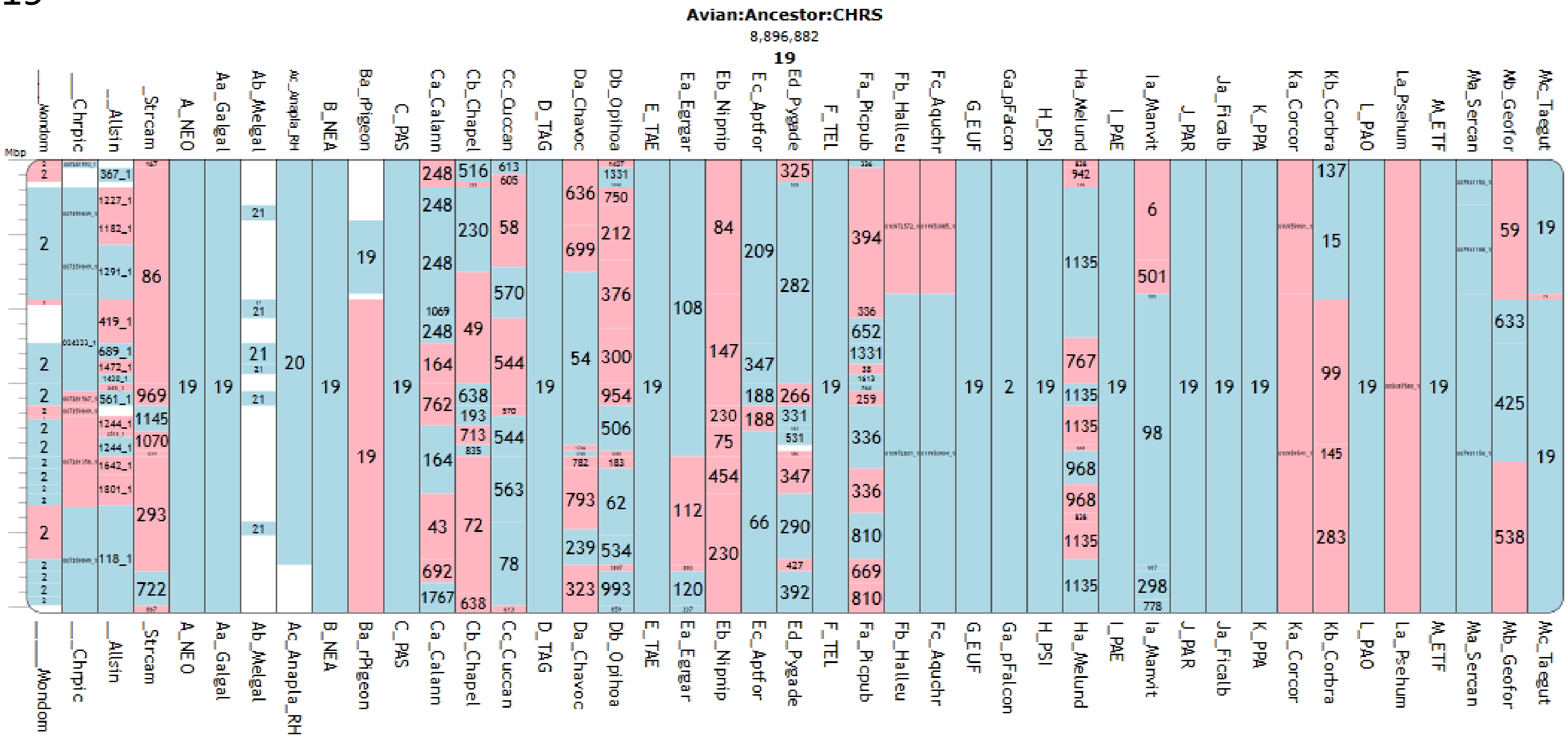
Avian:Ancestor:CHRS
8,896,882
19
Mbp
___Mondom
__Chrpic
__Allsin
_Strcam
A_NEO
Aa_Galgal
Ab_Melgal
Ac_Anapla_RH
B_NEA
Ba_rPigeon
C_PAS
Ca_Calann
Cb_Chapel
Cc_Cuccan
D_TAG
Da_Chavoc
Db_Opihoa
E_TAE
Ea_Egrgar
Eb_Nipnip
Ec_Aptfor
Ed_Pygade
F_TEL
Fa_Picpub
Fb_Halleu
Fc_Aquchr
G_EUF
Ga_pFalcon
H_PSI
Ha_Melund
I_PAE
Ia_Manvit
J_PAR
Ja_Ficalb
K_PPA
Ka_Corcor
Kb_Corbra
L_PAO
La_Psehum
M_ETF
Ma_Sercan
Mb_Geofor
Mc_Taegut

AVI20

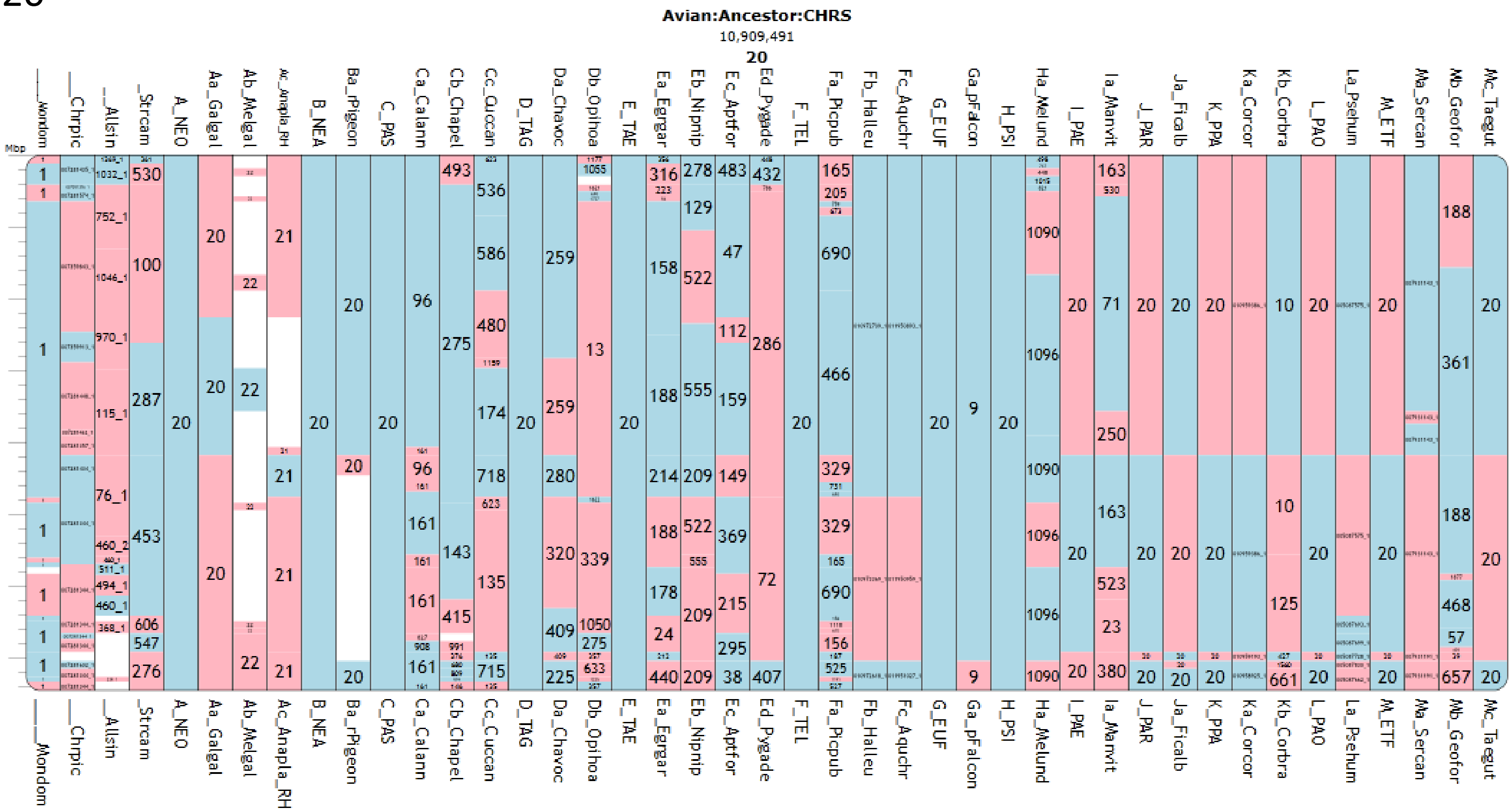
Avian:Ancestor:CHRS
10,909,491
20
Mbp
___Mondom
___Chrpic
__Allsin
_Strcam
A_NEO
Aa_Galgal
Ab_Melgal
Ac_Anapla_RH
B_NEA
Ba_rPigeon
C_PAS
Ca_Calann
Cb_Chapel
Cc_Cuccan
D_TAG
Da_Chavoc
Db_Ophoa
E_TAE
Ea_Egrgar
Eb_Nipnip
Ec_Aptfor
Ed_Pygade
F_TEL
Fa_Picpub
Fb_Halleu
Fc_Aquchr
G_EUF
Ga_pFalcon
H_PSI
Ha_Melund
I_PAE
Ia_Manvit
J_PAR
Ja_Ficalb
K_PPA
Ka_Corcor
Kb_Corbra
L_PAO
La_Psehum
M_ETF
Ma_Sercan
Mb_Geofor
Mc_Taegut

AVI21

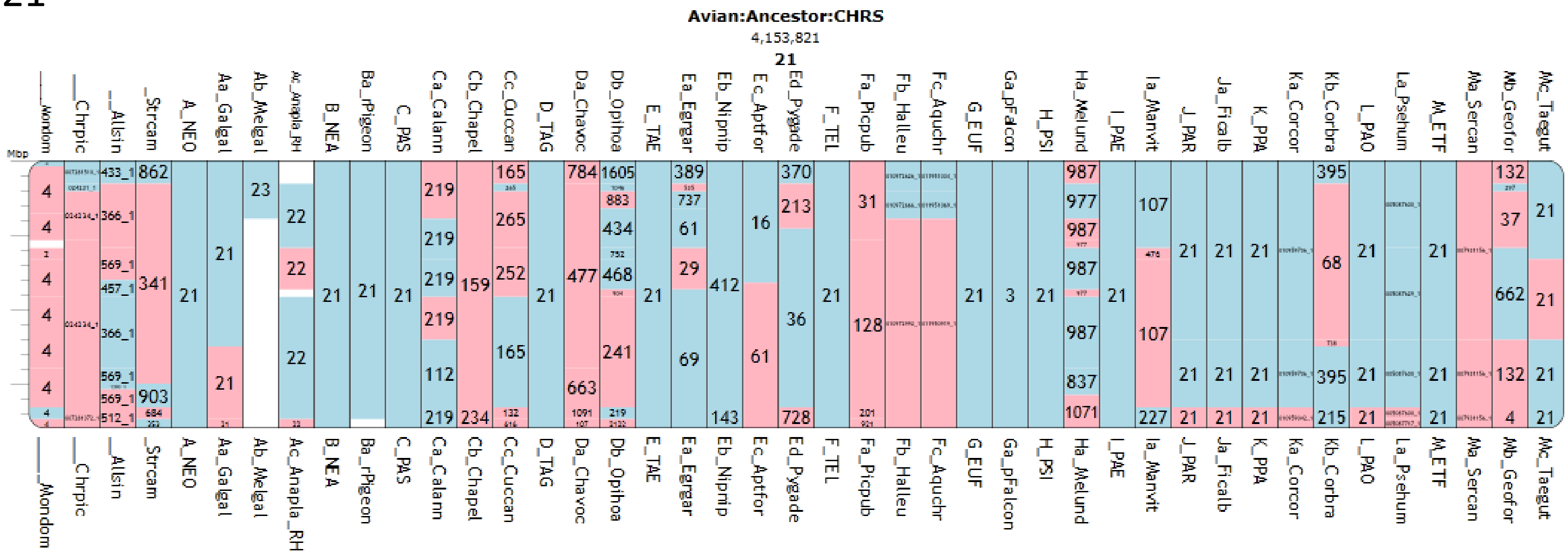

AVI22

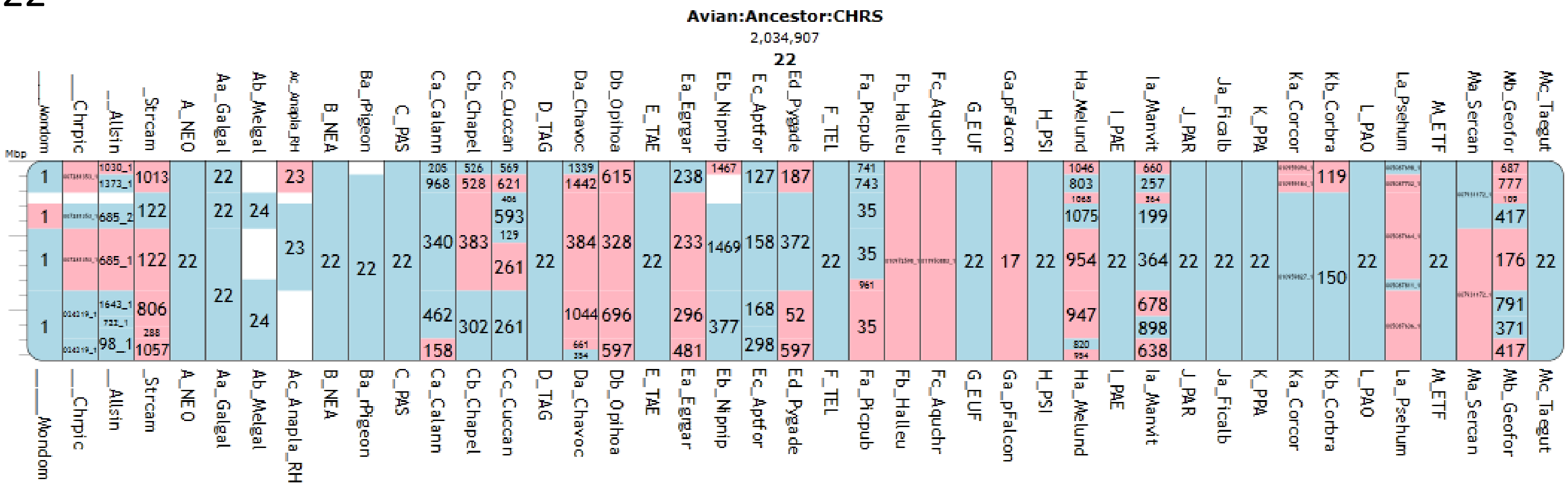
Avian:Ancestor:CHRS
2,034,907
22
Mbp
__Mondom
__Chrpic
__Allsin
_Strcam
A_NEO
Aa_Galgal
Ab_Melgal
Ac_Anapla_RH
B_NEA
Ba_rPigeon
C_PAS
Ca_Calann
Cb_Chapel
Cc_Cuccan
D_TAG
Da_Chavoc
Db_Opihoa
E_TAE
Ea_Egrgar
Eb_Nipnip
Ec_Aptfor
Ed_Pygade
F_TEL
Fa_Picpub
Fb_Halleu
Fc_Aquchr
G_EUF
Ga_pFalcon
H_PSI
Ha_Melund
I_PAE
Ia_Manvit
J_PAR
Ja_Ficalb
K_PPA
Ka_Corcor
Kb_Corbra
L_PAO
La_Psehum
M_ETF
Ma_Sercan
Mb_Geofor
Mc_Taegut

AVI23

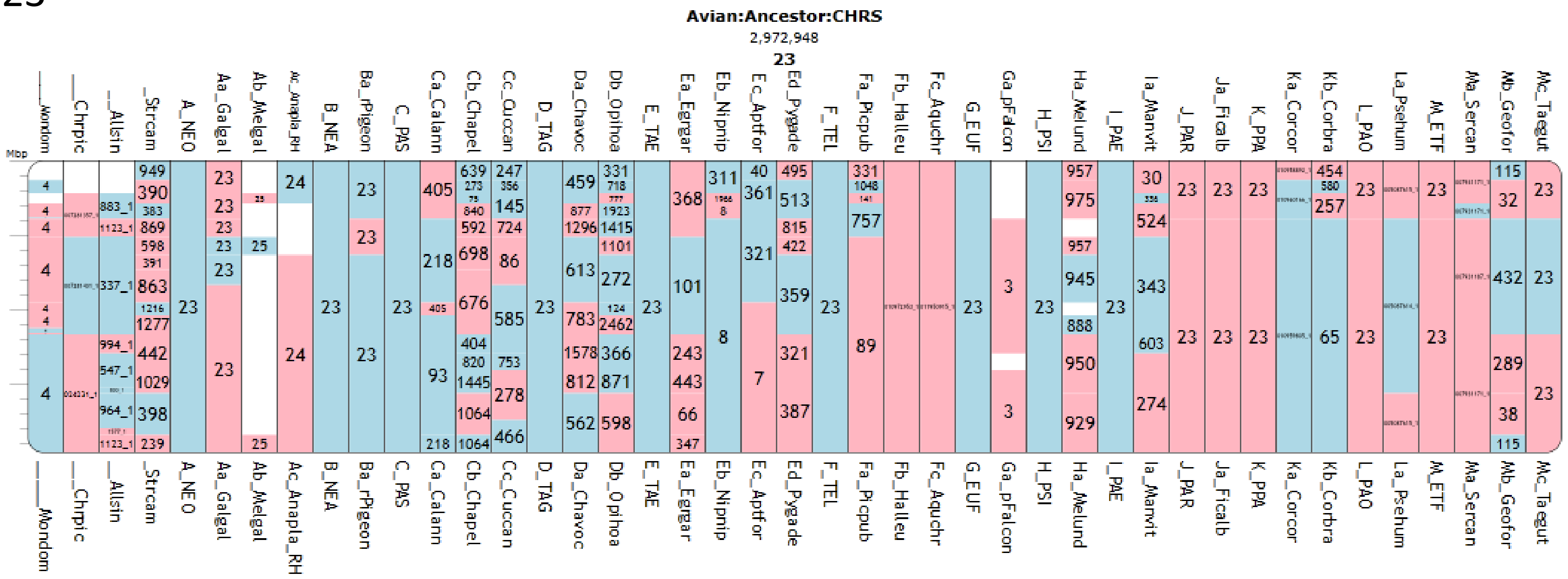

AVI24

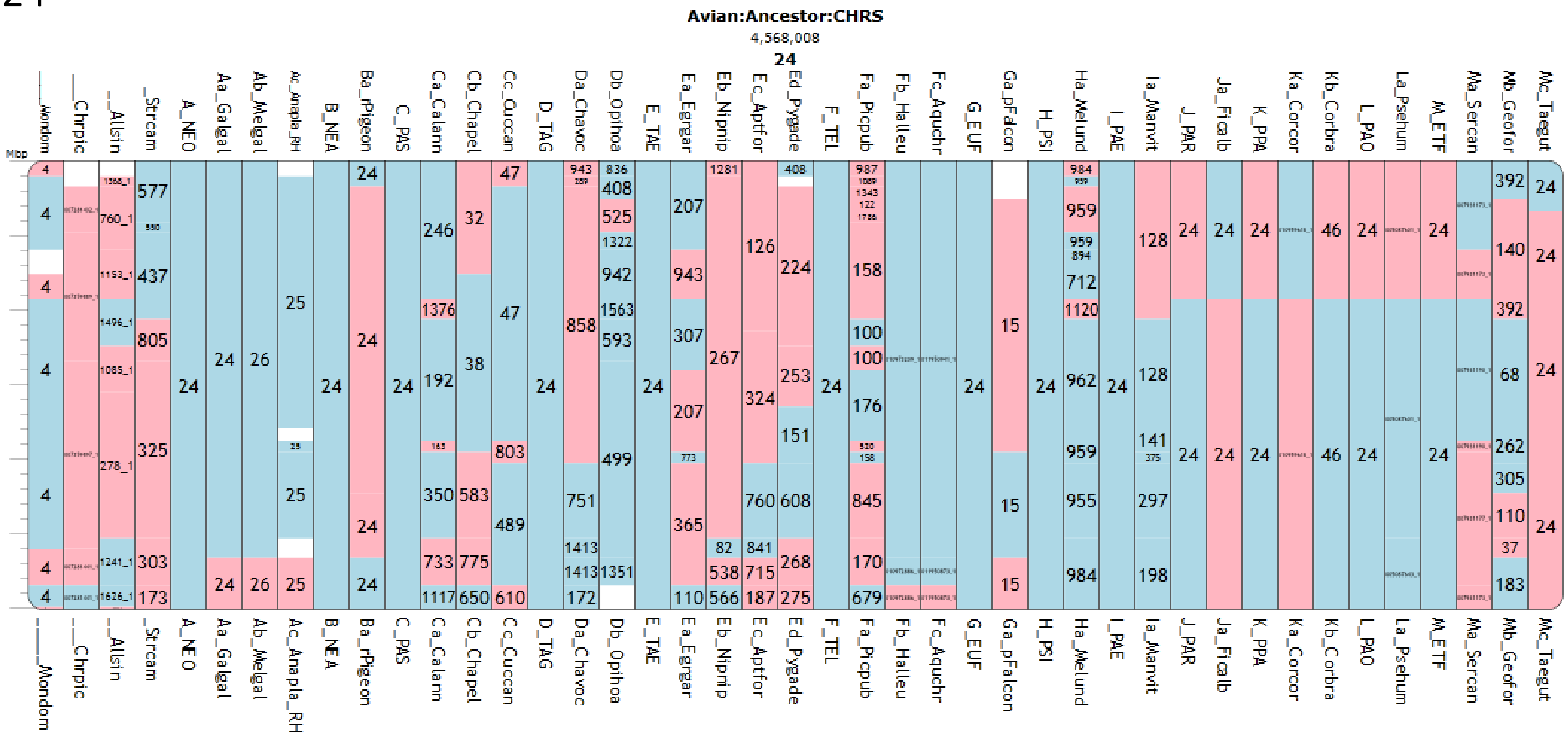
Avian:Ancestor:CHRS
4,568,008
24
Mbp
___Mondom
___Chrpic
__Allsin
_Strcam
A_NEO
Aa_Galgal
Ab_Melgal
Ac_Anapla_RH
B_NEA
Ba_rPigeon
C_PAS
Ca_Calann
Cb_Chapel
Cc_Cuccan
D_TAG
Da_Chavoc
Db_Opihoa
E_TAE
Ea_Egrgar
Eb_Nipnip
Ec_Aptfor
Ed_Pygade
F_TEL
Fa_Picpub
Fb_Halleu
Fc_Aquchr
G_EUF
Ga_pFalcon
H_PSI
Ha_Melund
I_PAE
Ia_Manvit
J_PAR
Ja_Ficalb
K_PPA
Ka_Corcor
Kb_Corbra
L_PAO
La_Psehum
M_ETF
Ma_Sercan
Mb_Geofor
Mc_Taegut

AVI26

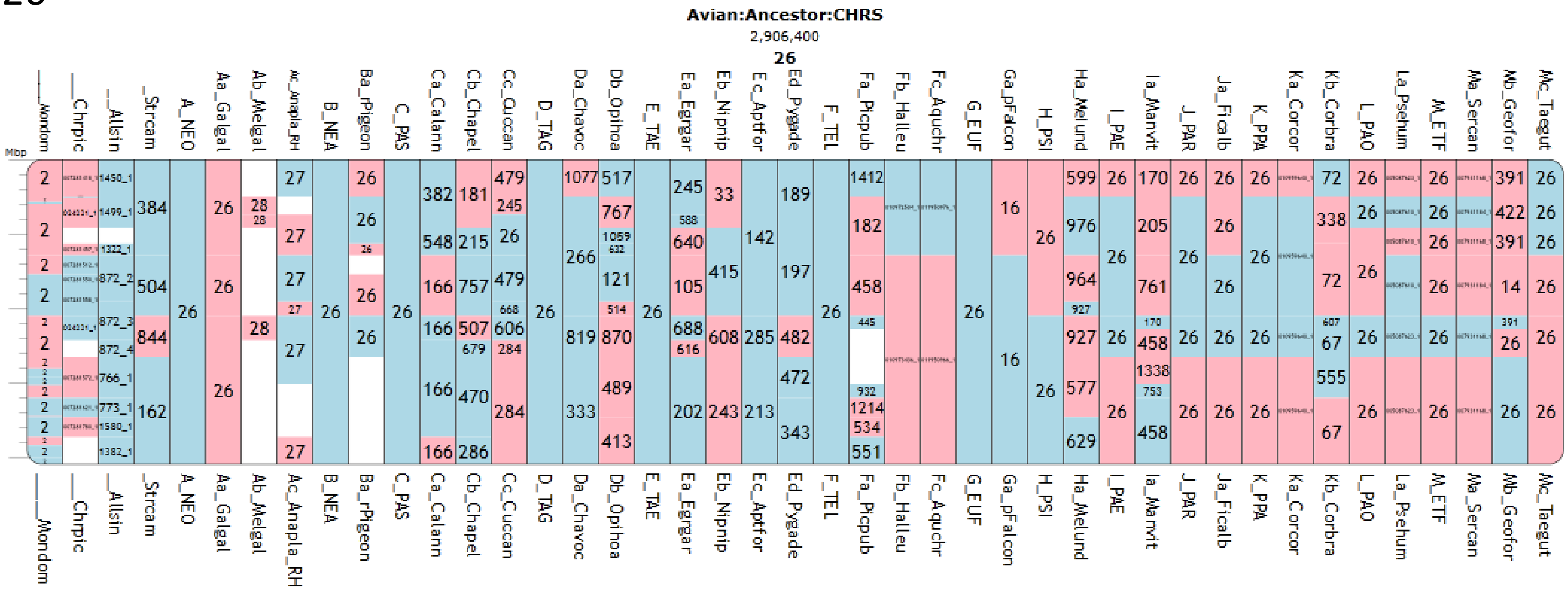
Avian:Ancestor:CHRS
2,906,400
26
Mbp
__Mondom
__Chrpic
__Allsin
_Strcam
A_NEO
Aa_Galgal
Ab_Melgal
Ac_Anapla_RH
B_NEA
Ba_rPigeon
C_PAS
Ca_Calann
Cb_Chapel
Cc_Cuccan
D_TAG
Da_Chavoc
Db_Opihoa
E_TAE
Ea_Egrgar
Eb_Nipnip
Ec_Aptfor
Ed_Pygade
F_TEL
Fa_Picpub
Fb_Halleu
Fc_Aquchr
G_EUF
Ga_pFalcon
H_PSI
Ha_Melund
I_PAE
Ia_Manvit
J_PAR
Ja_Ficalb
K_PPA
Ka_Corcor
Kb_Corbra
L_PAO
La_Psehum
M_ETF
Ma_Sercan
Mb_Geofor
Mc_Taegut

AVI27

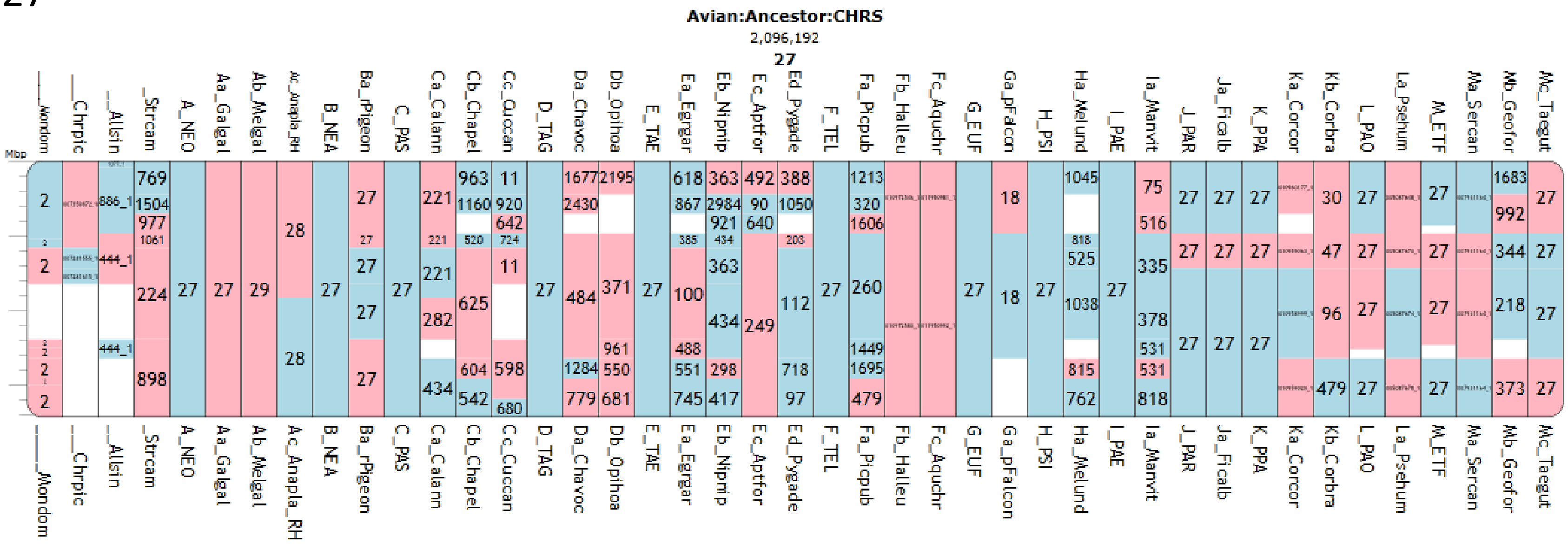

AVI28

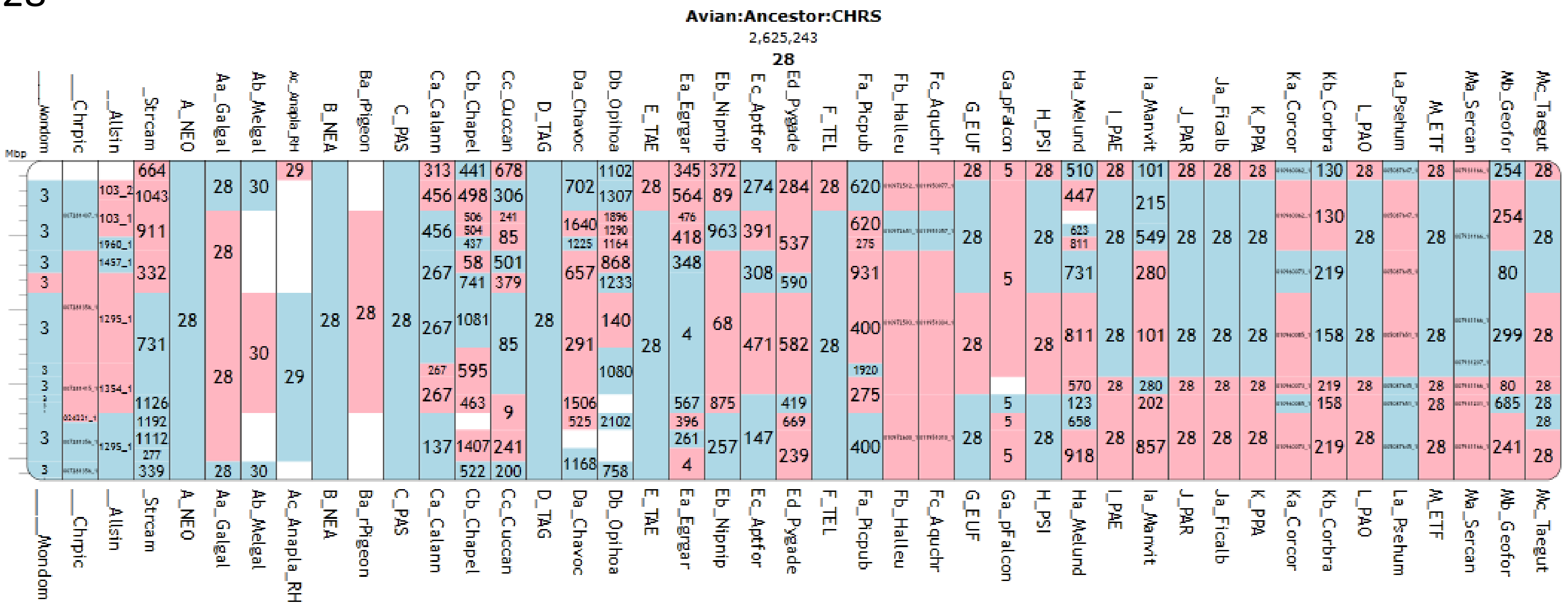

Avian:Ancestor:CHRS
2,625,243
28
Mbp
___Mondom
___Chrpic
__Allsin
_Strcam
A_NEO
Aa_Galgal
Ab_Melgal
Ac_Anapla_RH
B_NEA
Ba_rPigeon
C_PAS
Ca_Calann
Cb_Chapel
Cc_Cuccan
D_TAG
Da_Chavoc
Db_Opihoa
E_TAE
Ea_Egrgar
Eb_Nipnip
Ec_Aptfor
Ed_Pygade
F_TEL
Fa_Picpub
Fb_Halleu
Fc_Aquchr
G_EUF
Ga_pFalcon
H_PSI
Ha_Melund
I_PAE
Ia_Manvit
J_PAR
Ja_Ficalb
K_PPA
Ka_Corcor
Kb_Corbra
L_PAO
La_Psehum
M_ETF
Ma_Sercan
Mb_Geofor
Mc_Taegut

AVIZ

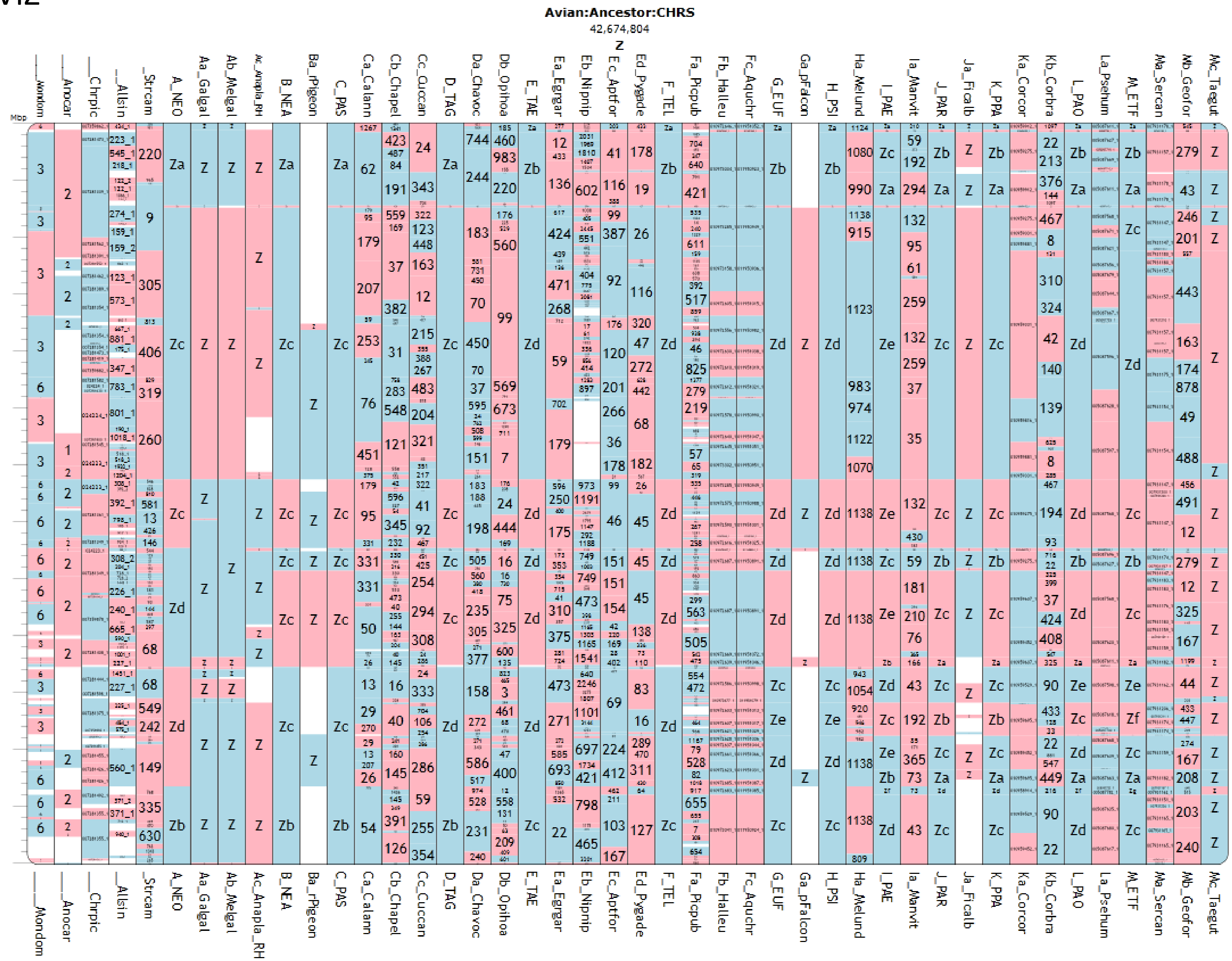
Avian:Ancestor:CHRS
42,674,804
Z
Mbp
__Mondom
__Anocar
__Chrpic
__Allsin
_Stream
A_NEO
Aa_Galgal
Ab_Melgal
Ac_Anapla_RH
B_NEA
Ba_Pigeon
C_PAS
Ca_Calann
Cb_Chapel
Cc_Cuccan
D_TAG
Da_Chavoc
Db_Opihoa
E_TAE
Ea_Egrgar
Eb_Nipnip
Ec_Aptfor
Ed_Pygade
F_TEL
Fa_Picpub
Fb_Halleu
Fc_Aquchr
G_EUF
Ga_pFalcon
H_PSI
Ha_Melund
I_PAE
Ia_Manvit
J_PAR
Ja_Ficalb
K_PPA
Ka_Corcor
Kb_Corbra
L_PAO
La_Psehum
M_ETF
Ma_Sercan
Mb_Geofor
Mc_Taegut
